# Lipocalins in Arthropod Chemical Communication

**DOI:** 10.1093/gbe/evab091

**Published:** 2021-04-30

**Authors:** Jiao Zhu, Alessio Iannucci, Francesca Romana Dani, Wolfgang Knoll, Paolo Pelosi

**Affiliations:** 1 Austrian Institute of Technology GmbH, Biosensor Technologies, Tulln, Austria; 2 Faculty of Biology, Institute of Molecular Physiology, Johannes Gutenberg-Universität Mainz, Mainz, Germany; 3 Departement of Biology, University of Firenze, Sesto Fiorentino, Italy

**Keywords:** lipocalins, odorant-binding proteins, chemical communication, insects, arthropods, phylogenesis

## Abstract

Lipocalins represent one of the most successful superfamilies of proteins. Most of them are extracellular carriers for hydrophobic ligands across aqueous media, but other functions have been reported. They are present in most living organisms including bacteria. In animals they have been identified in mammals, molluscs, and arthropods; sequences have also been reported for plants. A subgroup of lipocalins, referred to as odorant-binding proteins (OBPs), mediate chemical communication in mammals by ferrying specific pheromones to the vomeronasal organ. So far, these proteins have not been reported as carriers of semiochemicals in other living organisms; instead chemical communication in arthropods is mediated by other protein families structurally unrelated to lipocalins. A search in the databases has revealed extensive duplication and differentiation of lipocalin genes in some species of insects, crustaceans, and chelicerates. Their large numbers, ranging from a handful to few dozens in the same species, their wide divergence, both within and between species, and their expression in chemosensory organs suggest that such expansion may have occurred under environmental pressure, thus supporting the hypothesis that lipocalins may be involved in chemical communication in arthropods.

Significance

It has been known for many years that mammalian olfaction is mediated by carrier proteins, named Odorant-binding protein (OBP) belonging to the lipocalin family. In insects, instead, odorant and pheromones are transported by proteins defined with the same name of OBPs, but of completely different structure. Other classes of soluble proteins also act as semiochemical carriers in arthropods, such as chemosensory proteins and Niemann–Pick class C2 proteins.

In this work, we report a large duplication and differentiation of lipocalins in arthropods, often expressed in antennae and other chemosensory organs. These findings extend the number of semiochemical carriers in arthropods, opening a new field to investigate.

## Introduction

Chemical communication in mammals and in arthropods utilizes small carrier proteins to ferry hydrophobic pheromones and odorants in both directions: release of chemical messages from specialized glands into the environment and detection of the same chemicals through chemoreception structures (Pelosi et al. 2018).

Until recently, such tasks were known to be performed by some lipocalins, referred to as odorant-binding proteins (OBPs) in mammals (Pelosi et al. 1982), and by two unrelated classes of soluble proteins in insects, the insect OBPs (Vogt and Riddiford 1981), structurally different from vertebrate OBPs, and the chemosensory proteins (CSPs), again unrelated to the other two classes (McKenna et al. 1994; Pikielny et al. 1994; Zhu et al. 2019). Later, another family of soluble proteins has been also recognized as semiochemical carriers, Niemann–Pick C2 (NPC2), present in insects together with the other two above-mentioned families (Ishida et al. 2014; Pelosi et al. 2014; Zhu et al. 2018). More interestingly, NPC2s seem to represent the main carrier proteins of chemical communications in most of noninsect arthropods (Iovinella et al. 2016, 2018; Zheng et al. 2018). In fact, only one or two genes encoding CSPs are present in the genomes of some crustaceans, whereas they are likely absent in Chelicerata (Pelosi et al. 2018).

On the other hand, although insect OBPs, as characterized by their typical 6-cysteine pattern and three-dimensional scaffolding, have not been described in other arthropods, proteins with some sequence similarity have been reported with only few members (2–5) in Chelicerata (Eliash et al. 2017, 2019; Renthal et al. 2017; Vizueta et al. 2017), but much better represented (more than 20 sequences) in myriapods (Vizueta et al. 2018). These proteins are currently referred to as “OBP-like,” and may have evolved in parallel with insect OBPs from a common precursor. Another family of putative carrier proteins, named candidate carrier proteins, have been recently discovered in spiders and suggested to be involved in chemical communication. They present different characteristics from all the sequences mentioned so far, and their expression seems to be limited to spiders; further characterization is needed to suggest a role in chemical sensing (Vizueta et al. 2017, 2018).

Thus, in recent years, several families of proteins have been recognized or proposed as carriers of semiochemicals in arthropods, whereas in mammals a single class of odorant and pheromone-binding proteins has been described, the lipocalin OBPs. Lipocalins represent a large superfamily of proteins sharing a conserved scaffolding, the so-called β-barrel, but amino acid sequences can be highly divergent with identities as low as 10% (Flower et al. 2000; Ganfornina et al. 2000). They represent a large group of highly successful proteins, as witnessed by the fact that they have been adopted with several different functions in organisms all across the phylogenetic tree. Given their very different amino acid sequences, attempts have been made to identify conserved residues that could represent a reliable signature for lipocalins. Three small segments have been proposed, although with some variability in amino acid sequences. However, the most strict requirement is the signature -G-X-W-, which is close to the N-terminus and has to be present in all lipocalins (Flower 1996). We asked therefore whether lipocalins similar to the OBPs of vertebrates might also be involved in binding and carrying semiochemicals in invertebrates, in particular in insects and other arthropods.

### The OBPs of Vertebrates

A group of lipocalins, interesting for their role in chemical communication, comprises soluble polypeptides named OBPs for their function of complexing semiochemicals both in the nose, where they ferry them to olfactory receptors, and in pheromone glands, where they assist solubilizing pheromones and releasing them in the environment (Pelosi 1994; Tegoni et al. 2000).

The first member of vertebrate OBPs was discovered in the bovine nose (Pelosi et al. 1982; Bignetti et al. 1985; Pevsner et al. 1985; Bianchet et al. 1996; Tegoni et al. 1996) and soon after orthologs have been isolated from the nasal tissues of rat, pig, mouse, and other mammals (Pevsner et al. 1988; Dal Monte et al. 1991; Pes and Pelosi 1995; Lobel et al. 1998; Pes et al. 1998; Tegoni et al. 2000; Vincent et al. 2000). The wide genome information currently available allow us to identify genes encoding OBPs in many species of mammals and other vertebrates. However, experimental projects which have identified OBPs at the protein level and studied their structure and functions are very limited when compared with the more active research in the field of insect OBPs. The great majority of the work has been focused on mammals, with only a couple of reports in amphibians (Lee et al. 1987; Millery et al. 2005). Although genes encoding lipocalins can be identified in the genomes of other vertebrates and chordates, it is not easy to recognize those belonging to the OBP group in the absence of expression and functional information, when analyzing such genes in nonmammalian species. Even in mammals, gene annotation is still incomplete, thus making it difficult to establish with confidence the number of genes encoding OBPs in each species. Based on current information, the number of active genes encoding OBPs in each mammalian species is quite low (3–5 in most cases), compared with those of insects, that range from a minimum of 12 to more than 100 (Tegoni et al. 2000; Pelosi et al. 2018).

On the basis of sequence similarity, OBPs of mammals can be grouped into two sub-classes, those originally classified as OBPs and comprising the first bovine, rat and pig members, and those first identified in the urine of rodents and referred to as MUPs (major urinary protein) in the mouse, a2-u in the rat, in the saliva of the pig SAL (salivary lipocalin), and in the seminal fluid of the rabbit (Marchese et al. 1998; Mastrogiacomo et al. 2014; Zhu et al. 2017). These proteins have been shown to be associated with pheromones, usually produced in the same gland, when excreted (Bacchini et al. 1992; Robertson et al. 1993; Marchese et al. 1998; Cavaggioni et al. 2003). They are likely involved in solubilizing hydrophobic pheromones and helping their release in the environment through the deposition of urine, saliva, or other secretions. The same proteins have been also detected in the nose (Marchese et al. 1998; Mastrogiacomo et al. 2014; Zhu et al. 2017), showing thus a dual role in delivering and detecting chemical messages.

At the protein level, several works have detected OBPs in the nasal region, but not in the olfactory area of mice and other mammals. In fact, glands expressing OBPs in the nose are located in the vomeronasal organ, in the nasal septum or in the respiratory region of the nasal epithelium (Avanzini et al. 1987; Pevsner et al. 1988; Ohno et al. 1996; Utsumi et al. 1999). This fact, together with the small number of OBPs found in mammals and with the lack of functional OBPs in humans (the only OBP is expressed at very low levels) strongly suggested that OBPs in mammals are tuned to specific pheromones rather than being carriers for general odors (Pelosi 2001).

### Lipocalins in Arthropods

The presence of lipocalins in insects and in crustaceans has been known for many years, but only a few sequences were reported with different functions, and are classified under the following groups:

- Lazarillo lipocalins are similar to vertebrates Apolipoprotein D and have been reported in grasshoppers (Sánchez et al. 2000), *Drosophila* (Ruiz et al. 2014), termites (Yaguchi et al. 2018), and other arthropods. Diverse functions have been attributed to these proteins, from axonal guidance to carriers for small ligands, including pheromones;
- bilin-binding proteins, biliverdin-binding protein, and insecticyanin represent another group of lipocalins with the function of binding pigments (porphirinic bilin) and are responsible for the blue color of the hemolymph or of the wings in some Lepidoptera (Holden et al. 1987; Schmidt and Skerra 1994);
- crustacyanin is present in the shell of shrimps and lobsters (Keen et al. 1991; Dellisanti et al. 2003). This protein is similar in amino acid sequence to retinol-binding protein of vertebrates, and is bound to astaxanthin, a blue pigment probably helping camouflage. During cooking, crustacyanin is denatured and releases the ligand, which then undergoes a conformational change, thus changing its color to red (Cianci et al. 2002). We expect that proteins binding the same ligand in different species, as is the case of insecticyanin, bilin-binding proteins, and crustacyanin, should be well conserved across evolution, thus enabling us to hypothesize a function based on sequence identity. Unfortunately, the scattered information available in the literature for arthropod lipocalins does not allow us to establish reliable comparisons. With lipocalins of vertebrates, instead, we can easily recognize such proteins with well-defined functions, as in the case of retinol-binding protein (Newcomer et al. 1984) and apoliproprotein D (Eichinger et al. 2007), as their sequences are highly conserved between mammals and also between vertebrates.

In addition to the few (2–4) proteins of the above groups found in each species of arthropods, a massive gene expansion is reported in the hemipteran *Rhodnius prolixus*, vector of the Chagas disease, and other blood-sucking arthropods (Andersen and Montfort 2000; Montfort et al. 2000; Ribeiro et al. 2004; Andersen et al. 2005; Ganfornina et al. 2013). Such proteins are secreted in the saliva and include subgroups with different functions. Nitrophorins contain a heme group and transport NO, others carry amines, such as serotonin and norepinephrine, others act as blood coagulation inhibitors. A large expansion was also observed in the mite *Tetranichus urticae*, in whose genome 58 genes encoding lipocalins are reported. It has been suggested that such proteins might be involved in detoxification and resistance to xenobiotics (Van Leeuwen and Dermauw 2016).

In this work we have searched for lipocalin families in selected species of insects, Chelicerata and Crustacea, and found extensive duplication and differentiation of these genes. Based also on the observation that such genes often occur in clusters in the genome, as well as on the expression of some sequences in pheromone glands and antennae, we hypothesize that lipocalins might represent an additional class of semiochemical-binding proteins in arthropods.

## Materials and Methods

### First Set of Data

As a first step, we searched the Protein database at NCBI, using the word “lipocalin” associated with the name of the species. For Hexapoda, Crustacea, and Chelicerata, we searched selected species, representative of different Classes, for which a genome project was listed in the NCBI Genome database.

### Second Set of Data

For each species, we blasted all the sequences downloaded in our preliminary search against the Protein database, limiting the search to the same species. This second list was edited by discarding sequences that were too short, exceptionally long or did not contain the typical lipocalin signature (-G-X-W-). Moreover, where we noticed a distance particularly long between the predicted starting methionine and the lipocalin signature, we searched for another putative starting methionine using the SignalP-5.0 software with default parameters. In most cases, we identified a shorter signal peptide that was in better agreement with the prediction. Some of the sequences presented longer C-terminal segments, but we were not able to decide whether these were due to errors in the stop codons.

### Alignments and Final Set of Data

Alignments were performed using the online software ClustalW (https://www.genome.jp/tools-bin/clustalw) and the default parameters (gap open penalty: 10; gap extension penalty: 0.05; selected weight matrix: BLOSUM). Identical sequences were discarded, as well as those differing by a single amino acid substitution. We decided to consider such small differences more likely due to errors in sequencing, rather than to the existence of isoforms. These could be present, but more careful examination and repeated sequencing would be needed before drawing any conclusion. This procedure provided a final set of sequences for each species that were used for building phylogenetic trees.

### Phylogenetic Trees

Alignment of all the selected sequences of Hexapoda, Crustacea, and Chelicerata, performed with ClustalW and default parameters, was used to generate a tree with Neighbor-Joining method using the Kimura distance correction for the three subphyla. Visualization of the trees was done with the program FigTree, version 1.4.2 (https://github.com/rambaut/figtree/releases).

### Genome Organization

To investigate whether lipocalin genes are located in clusters within chromosomes we analyzed the location of lipocalin genes in the genomes of two lepidopterans, *Helicoverpa armigera* and *Bombyx mori*, as examples. The gene location was visually identified onto the target species reference genomes (*H. armigera*: PRJNA378437, *B. mori* PRJDB4947) using the Genome Data Viewer tool from NCBI.

### Expression of Lipocalin Genes

To search for the expression of lipocalin genes in organs of selected species, we blasted (TBLASTn) each protein sequence against the SRA databases in NCBI, using the accession numbers listed in supplementary table S1, Supplementary Material online. We only collected fragments that were 100% identical with our queries and mapped them on the whole sequence, eventually calculating the percent of sequence coverage for each lipocalin.

To evaluate the expression of *Helicoverpa armigera* sequences in different organs (antennae, pheromone glands, salivary glands and abdomen), we calculated the coverage of each protein on the basis of the number of hits (limited to those with 100% identity) found in the SRAs of each organ. Values were normalized with reference to the housekeeping gene NADH dehydrogenase (acc. no. AHJ91280.1), considered highly reliable (Yang et al. 2014).

## Results

Prompted by the large duplication and differentiation of lipocalins in *R. prolixus* and other blood-sucking arthropods, we asked whether similar phenomena could have occurred in other species of arthropods to verify the hypothesis that lipocalins could have also been adopted as carriers of semiochemicals in insects and other arthropods. We reasoned that such hypothesis might be only supported if all of the following three criteria were verified:

1. Should belong to a multigene family (a handful genes or more in each species).
2. Their sequences should be divergent not only between species, but also within the same species, suggesting that duplication and differentiation have occurred under environmental pressure.
3. Most of such genes should be expressed in chemosensory organs and pheromone glands.

### Search for Lipocalins in Arthropods

**Fig. 1.**
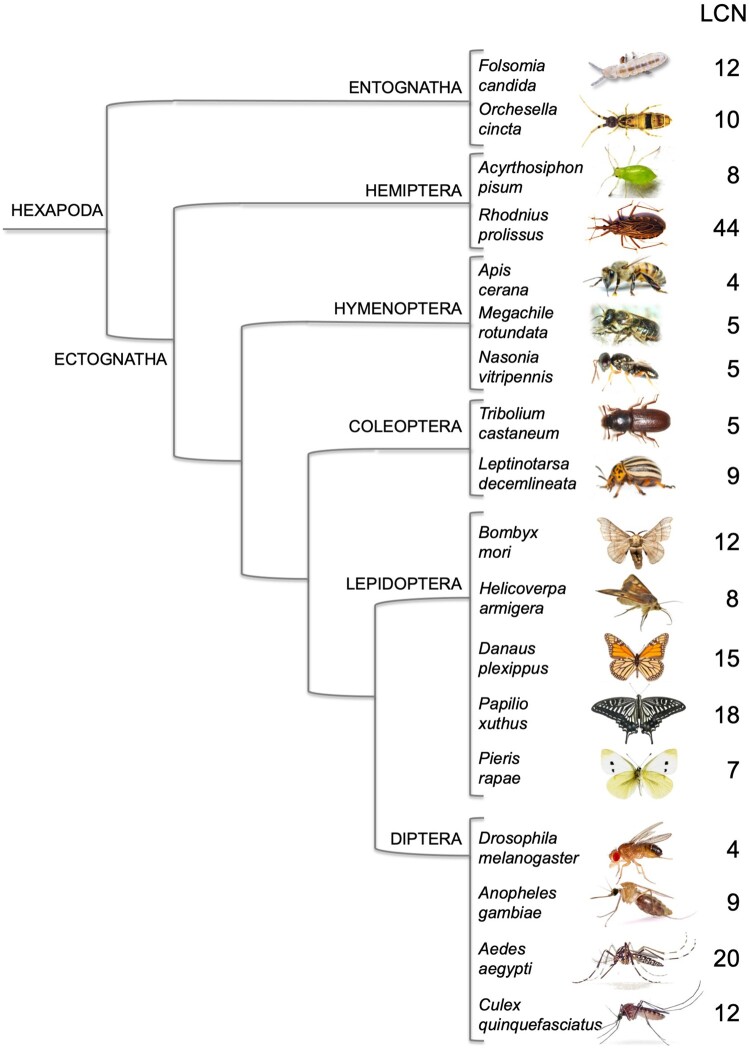
Number of genes encoding lipocalins (LCN) in selected species of insects. Orders and Classes are reported, but the tree does not reflect phylogenetic distances. Adapted from Giribet and Edgecombe (2012).

**Fig. 2.**
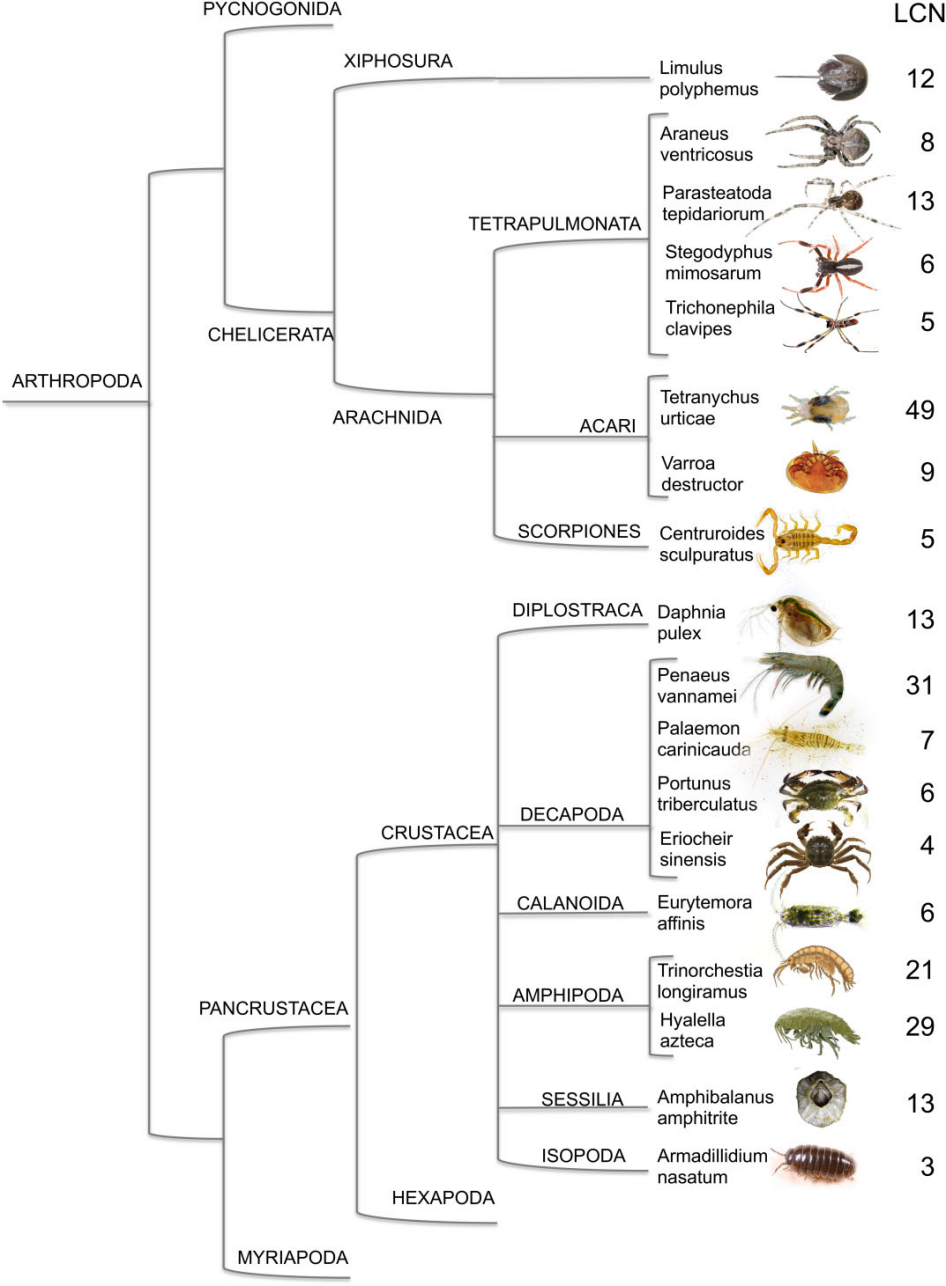
Number of genes encoding lipocalins (LCN) in selected species of Crustacea and Chelicerata. Orders and Classes are reported, but the tree does not reflect phylogenetic distances. Adapted from Giribet and Edgecombe (2012). In addition to those reported, we only found one or two LCN genes in the NCBI databases relative to the following Chelicerata: *Dinothrombium tinctorium*; *Euroglyphus maynei*; *Galendromus occidentalis*; *Ixodes scapularis*; *Leptotrombidium delicense*; *Rhipicephalus microplus*; *Sarcoptes scabiei*; *Tropilaelaps mercedesae*. We could not find any LCN genes in the following species. Crustacea: *Triops cancriformis*; *Procambarus virginalis*; *Lepidurus apus*; *Cherax destructor*; *Pandalus platyceros*; *Ligia exotica*; *Tisbe* sp.; *Pandalus platyceros*; *Apocyclops* sp.; *Semibalanus balanoides*; *Acartia tonsa*; *Eulimnadia texana*; *Calanus glacialis*; *Caridina multidentate*; *Oithona nana*; *Caligus rogercresseyi*; *Cherax destructor*; *Parhyale hawaiensis*; *Tigriopus japonicus*; *Calanus finmarchicus*; *Lepeophtheirus salmonis*. Chelicerata: *Acanthoscurria geniculate*; *Achipteria coleoptrata*; *Androctonus mauritanicus*; *Anelosimus studiosus*; *Brevipalpus yothersi*; *Cordylochernes scorpioides*; *Dermanyssus gallinae*; *Dermatophagoides farina*; *Dysdera sylvatica*; *Haemaphysalis longicornis*; *Hypochthonius rufulus*; *Ixodes ricinus*; *Latrodectus hesperus*; *Loxosceles reclusa*; *Mesobuthus martensii*; *Pardosa pseudoannulata*; *Platynothrus peltifer*; *Psoroptes ovis*; *Steganacarus magnus*; *Tachypleus tridentatus.* Finally, no LCN genes were identified for Pycnogonida or Myriapoda.

We searched the NCBI protein database along with the procedure reported in the “Materials and Methods” section, and obtained a set of sequences as reported in figures 1 and 2 classified by species, order, and class. In our work we have only considered Hexapoda, Crustacea, and Chelicerata. Nevertheless, based on a preliminary search, we could not find lipocalins in Pycnogonida, nor in Myriapoda, but at the same time we cannot exclude their presence in these organisms, due to still incomplete annotation.

In many of the species examined and cited in the legend of figure 2, we found only one or two genes encoding lipocalins or even none. In the others, that are reported in figures 1 and 2, numbers of lipocalins vary across a broad range from 3 to 49. The available data are still too scattered and limited to suggest whether the number of lipocalins in each species is related to phylogenetic position, to life cycle and habits or just to incomplete annotation, this last hypothesis being the most likely for Crustacea and Chelicerata. In any case, the fact that a number of lipocalin genes in the order of a dozen or higher is present in several species of arthropods represents an interesting starting point for considering at least some members of this protein family as putative semiochemical carriers.

### Sequence Comparison of Arthropod Lipocalins

**Fig. 3.**
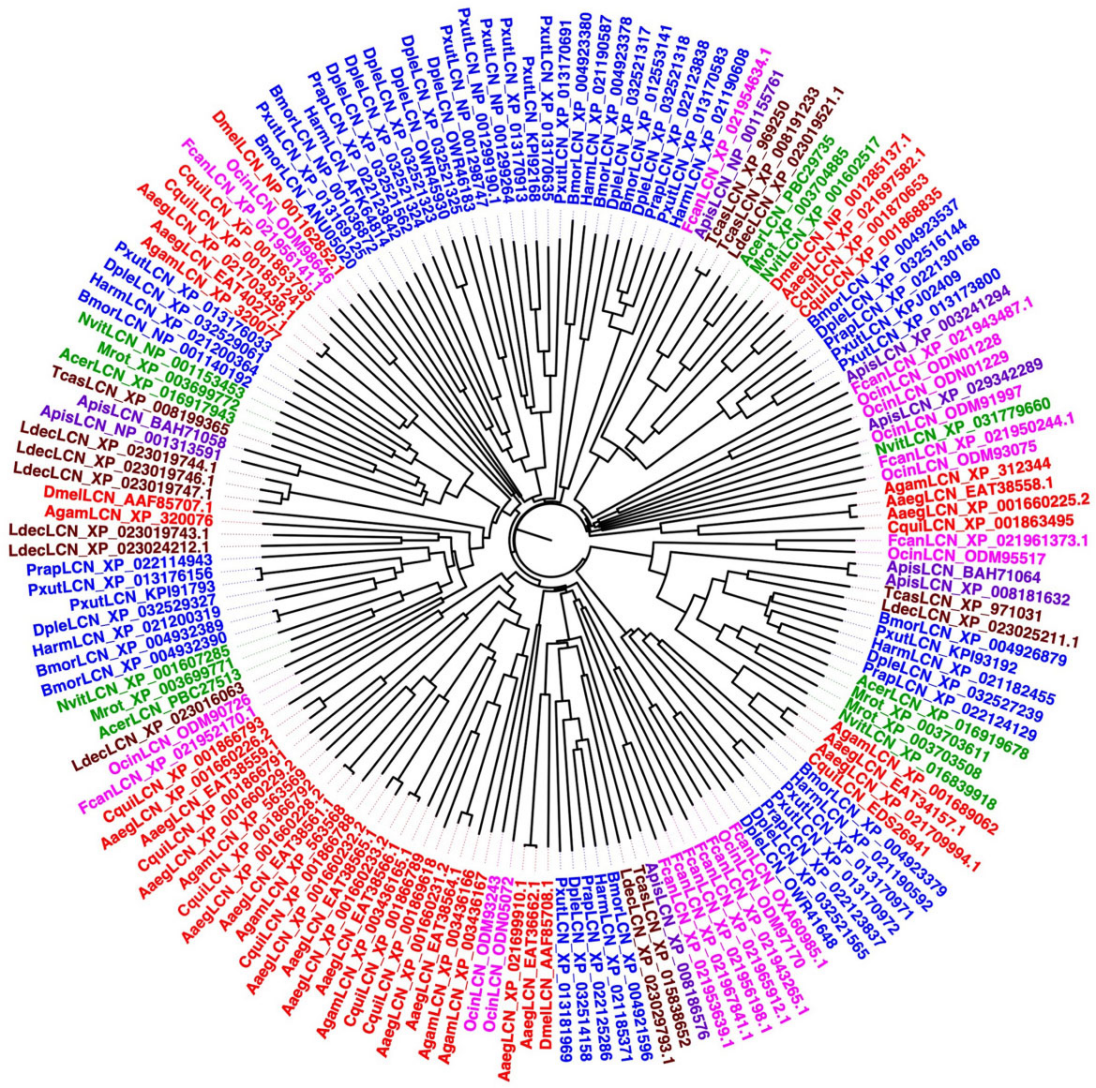
Neighbour-Joining tree of lipocalins identified in the genomes of selected species of Hexapoda. Apis: *Acyrthosiphon pisum*; Aaeg: *Aedes aegypti*; Agam: *Anopheles gambiae*; Acer: *Apis cerana*; Bmor: *Bombyx mori*; Cqui: *Culex quinquefasciatus*; Dple: *Danaus plexippus*; Dmel: *Drosophila melanogaster*; Fcan: *Folsomia candida*; Harm: *Helicoverpa armigera*; Ldec: *Leptinotarsa decemlineata*; Mrot: *Megachile rotundata*; Nvit: *Nasonia vitripennis*; Ocin: *Orchesella cincta*; Pxut: *Papilio Xuthus*; Prap: *Pieris rapae*; Tcas: *Tribolium castaneum*. The 44 sequences of *Rhodnius prolixus* are not included, because most of them segregate into a separate clade (see supplementary fig. S1, Supplementary Material online). Color code: Entognatha: magenta; Hemiptera: purple; Hymenoptera: green; Coleoptera: brown; Lepidoptera: blue; Diptera: red.

**Fig. 4.**
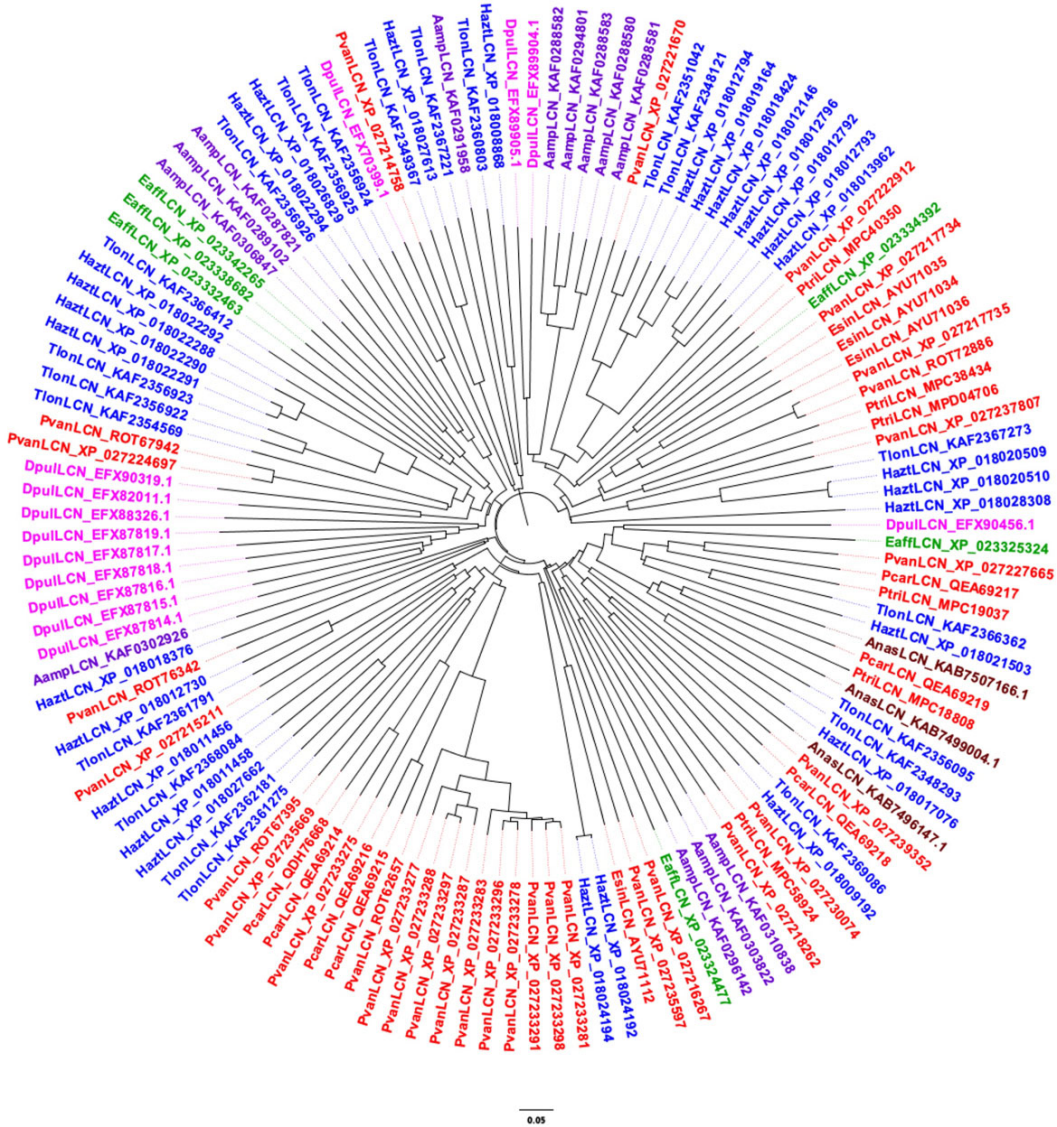
Neighbour-Joining tree of lipocalins identified in the genomes of selected species of Crustacea. Aamp: *Amphibalanus amphitrite*; Anas: *Armadillidium nasatum*; Dpul: *Daphnia pulex*; Esin: *Eriocheir sinensis*; Eaff: *Eurytemora affinis*; Hazt: *Hyalella azteca*; Pcar: *Palaemon carinicauda*; Pvan: *Penaeus vannamei*; Ptri: *Portunus trituberculatus*; Tlon: *Trinorchestia longiramus*. Color code: Diplostraca: magenta; Decapoda: red; Calanoida: green; Amphipoda: blue; Sessilia: purple; Isopoda: brown.

**Fig. 5.**
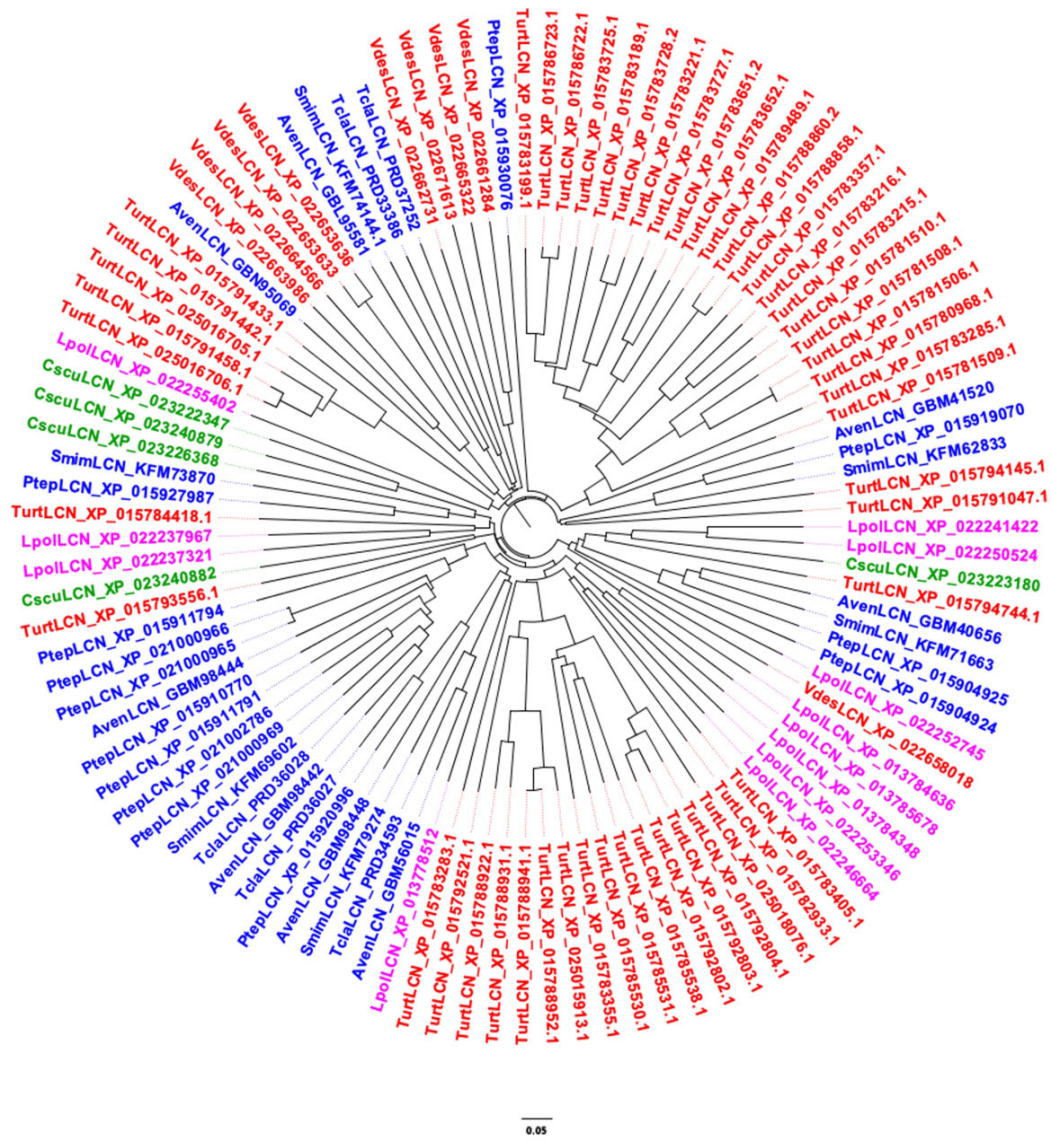
Neighbour-Joining tree of lipocalins identified in the genomes of selected species of Chelicerata. Aven: *Araneus ventricosus*; Cscu: *Centruroides sculpturatus*; Dtin: *Dinothrombium tinctorium*; Emay: *Euroglyphus maynei*; Gocc: *Galendromus occidentalis*; Isca: *Ixodes scapularis*; Ldel: *Leptotrombidium delicense*; Lpol: *Limulus polyphemus*; Ptep: *Parasteatoda tepidariorum*; Rmic: *Rhipicephalus microplus*; Ssca: *Sarcoptes scabiei*; Smim: *Stegodyphus mimosarum*; Turt: *Tetranychus urticae*; Tcla: *Trichonephila clavipes*; Tmer: *Tropilaelaps mercedesae*; Vdes: *Varroa destructor*. Color code: Xiphosura: magenta; Tetrapulmonata: blue; Acari: red; Scorpiones: green.

To verify the second of our three criteria, a marked divergence between and within species, we aligned the lipocalins of each species, finding identity values at the amino acid level around 20–30% for most pairs, but also occasional values as low as 10% or as high as 80%. Between different species we also detected similar values of identity. Such large divergence both between species and within the same species can be appreciated from the phylogenetic trees of figures 3–5 relative to Hexapoda, Crustacea, and Chelicerata, respectively. The tree reporting insect lipocalins (fig. 3) does not include those of *R. prolixus*. The reason is that most of the 44 proteins of this species, with only few exceptions, segregate in a clade clearly separated from the lipocalins of other insects (supplementary fig. S1, Supplementary Material online).

Looking at the phylogenetic trees of figures 3–5, instead, we can observe large divergence between species, but also marked differences between sequences of the same species. This can be easily appreciated by noticing that branches of the tree often contain members belonging to different species and different orders. The only exception is provided by the 49 lipocalins of *Tetranychus urticae*, most of which segregate into two clades of 22 and 17 members, probably indicating specific functions.

### Genome Localization

The genes encoding OBPs in insects have been reported to occur often in genomic clusters, being this a sign that they originated from gene duplication (Xu et al. 2003; Librado and Rozas 2013). Therefore, we wanted to verify whether this was the case also for at least some of the lipocalins reported in this study. Accordingly, we performed genome analysis for the 12 sequences of *B. mori* and the 8 of *H. armigera*, as representative examples. Indeed, we found that most of the genes (9 in *B. mori* and 5 in *H. armigera*) were located in two clusters in each species. The relative information is summarized in table 1, whereas a graphical representation is reported in supplementary figure S2, Supplementary Material online.

**Table 1.** Genome Location of *Bombyx mori* (Bmor) and *Helicoverpa armigera* (Harm) Lipocalin Genes

Protein	Location	Locus Tag
**Bmor_XP_004932390**	Chr 5	LOC101742419
**Bmor_XP_004932389**	Chr 5	LOC101742419
**Bmor_NP_001140192**	Chr 5	LOC100286767
**Bmor_ANU05020**	Chr 25	Chbp
**Bmor_NP_001036872**	Chr 25	LOC692416
**Bmor_XP_004923379**	Chr 25	LOC101745459
**Bmor_XP_004923378**	Chr 25	LOC101745319
**Bmor_XP_012553141**	Chr 25	LOC101745175
**Bmor_XP_004923380**	Chr 25	LOC101745601
Bmor_XP_004921596	Chr 10	LOC101739203
Bmor_XP_004926879	Chr 16	LOC101742345
Bmor_XP_004923537	Chr 6	LOC101739113
**Harm_XP_021200319.1**	NW_018395398.1	B5X24_HaOG215900
**Harm_XP_021200364.1**	NW_018395398.1	B5X24_HaOG215901
**Harm_XP_021190587.1**	NW_018395510.1	B5X24_HaOG202918
**Harm_XP_021190592.1**	NW_018395510.1	B5X24_HaOG202919
**Harm_XP_021190608.1**	NW_018395510.1	B5X24_HaOG202921
Harm_XP_021182455.1	NW_018395414.1	B5X24_HaOG207595
Harm_XP_021185371.1	NW_018395436.1	B5X24_HaOG212519
Harm_AFK64814.1	Unannotated	Unannotated

Note.—Genes in bold font are clustered (supplementary Fig. S2, Supplementary Material online). *H. armigera* genome is currently not chromosome anchored, however, genes are present in proximity on the same scaffolds. In particular, Harm_XP_021200319.1 and Harm_XP_021200364.1 are found in close proximity on the scaffold NW_018395398.1, whereas Harm_XP_021190587.1, Harm_XP_021190592.1, and Harm_XP_021190608.1 are found in close proximity on the scaffold NW_018395510.1 (supplementary fig. S2, Supplementary Material online). For this analysis, we used the reference genomes Harm1.0 (acc. no. PRJNA378437) and Bmor_2016v1.0 (acc. no. PRJDB4947).

### Expression of Lipocalins in Sensory Organs of Arthropods

Having verified the first two criteria for putatively assigning a role in chemical communication to lipocalins in arthropods, we decided to investigate whether at least some of the sequences found in the genomes were expressed in the antennae. To this task, we took advantage of several transcriptome projects, mainly focused on insect species, whose results are available in the databases. Accordingly, for each species we blasted the set of lipocalins against the SRA database, using the specific SRX files, relative to antennae transcripts, as well as to other organs, reported in the NCBI database. For each sequence, we could find a variable number of short reads, that we compared with the entire sequence to support the expression of each sequence in the target organ. Given the wide divergence of amino acid sequences within the same species, we assumed that even a limited coverage was enough to confidently assume that the relative gene was expressed in the antennae. Supplementary table S1, Supplementary Material online summarizes the data obtained, also reporting for each sequence the percentage that was verified in the SRX files relative to each organ.

We can first observe that most of the lipocalins encoded in the genome are expressed in the antennae, with the only notable exception of *R. prolixus*, in which only 9 of the 44 genes are transcripted in the antennae. This fact indicates that the set of lipocalins of *R. prolixus* represent a separate group, not only different in sequence from those of other insects, but also in function. Regarding the nine lipocalins of *Varroa destructor*, the only noninsect arthropod for which we could perform a search in different organs, we found transcripts for all of them in the forelegs (where the main olfactory structure, referred to as pit organ, is located) of phoretic mites, for eight in the forelegs of reproductive mites, and for seven in the rear legs.

It is perhaps worth emphasizing a couple of observations further supporting a role of lipocalin in chemoreception. One of the *A. gambiae* lipocalins (Agam-XP_320076) and two of *A. aegypti* (AaegLCN_EAT38564 and AaegLCN_XP_001660231) are female antennae specific. Similarly, two of the sequences of *V. destructor* (VdesLCN_XP_022653633 and VdesLCN_XP_022653636) were detected in the first pair of legs transcripts, but could not be found in the rear legs. In the same mite, revisiting the data obtained from a proteomic study (Iovinella et al. 2018), we found that two lipocalins (acc. no. XP_022665322.1, XP_022661284.1) are expressed in the legs and in the capitulum at the protein level with one (XP_022661284.1) being more abundant in the first pair of legs, whereas two more (XP_022664566.1, XP_022658018.1) were found at low abundance in only a few samples.

**Fig. 6.**
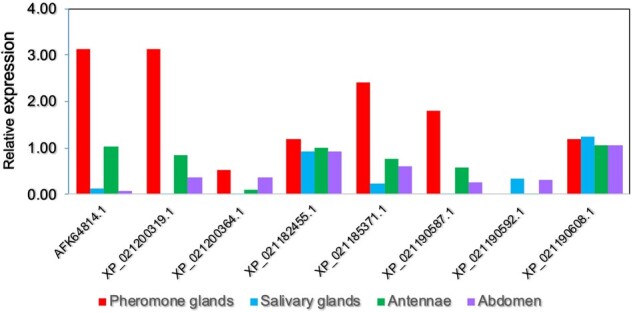
Expression of lipocalins in different tissues of *H. armigera*. The relative expression was evaluated based on the number of SRA found for each sequence in the transcriptomes of pheromone glands, antennae, salivary glands, and abdomen. Values were normalized with reference to the housekeeping gene NADH dehydrogenase (acc. no. AHJ91280.1) (Yang et al. 2014). Only SRAs with 100% identity to the query sequence were taken into account.

We next investigated the relative expression of LCN genes in pheromone glands, antennae, salivary glands, and abdomen of the moth *H. armigera*. This analysis was based on transcriptomic information evaluating the number of times that the same sequence occurred in each tissue. The results illustrated in figure 6 show that out of the eight LCN genes of *H. armigera*, four are predominantly expressed in pheromone glands and antennae, whereas two are present at similar levels in all four tissues examined. The remaining two were only found at very low levels in the four tissues examined, and could be expressed elsewhere.

Overall, the large number of lipocalins expressed in antennae (supplementary table S1, Supplementary Material online), together with the high levels of some transcripts of these genes in pheromone glands (fig. 6), seem to meet the third requirement exemplified above.

Thus, all three criteria for tentatively assigning a role of semiochemical carriers to lipocalins have been verified and we can suggest that these binding proteins might represent an additional class of carriers for odorants and pheromones in arthropods. Experimental evidence will be required to further support these preliminary data, such as localization of the proteins and their genes in chemosensilla, ligand-binding assays, and behavior studies associated with silencing of selected genes.

## Discussion

Lipocalins represent one of the most successful families of proteins in terms of stability and have been adopted for a large number of roles, mostly being carriers for small hydrophobic compounds, but also performing other unrelated functions. Members of this superfamily have been identified in eukaryotic organisms and bacteria. In animals, within a context of chemical communication, lipocalins have been reported so far only in vertebrates, mainly in mammals, but it is reasonable to suspect that such successful proteins could have been adopted as carriers for semiochemicals also in other groups of metazoans.

In this work, we have reported the presence of genes encoding lipocalins in several species of arthropods, namely Hexapoda, Crustacea, and Chelicerata. We also suggest that the relatively large numbers of these genes found in some species may be the result of extensive duplication and differentiation generated under environmental pressure. This observation, together with the expression of transcription products of most of such genes in the antennae of insects and the forelegs of the mite *V. destructor*, as well as in the pheromone glands of *H. armigera*, may suggest a role of some insect lipocalins in chemical communication as semiochemical carriers. On the other hand, the expression of semiochemical carrier proteins in parts of the body different from the classical chemoreception organs and pheromone glands is not surprising. In fact, as an example, legs usually house chemosensilla, whereas carrier proteins are also produced in salivary glands and reproductive organs, where they can perform functions not necessarily related to chemical communication (Pelosi et al. 2018). Certainly, experimental evidence should be provided with ligand-binding assays and localization of these proteins and their encoding genes in chemosensilla before a role of these lipocalins in chemical communication could be established.

One of the questions arising is why insects use such a variety of carrier proteins. Related to such question is also the numbers of expressed genes for each family, which vary over large extents according to the species. For example, OBPs range from just a dozen in aphids to around 20 in honeybees and locusts, 40–50 in Lepidoptera and Coleoptera, reaching more than 100 in flies and mosquitoes (Pelosi et al. 2018; Zhu et al. 2019). On the other hand, CSPs are generally less represented, with numbers lower than 20, except for locusts, where at least 70 genes encoding CSPs have been detected (Zhou et al. 2013). The third class of semiochemical carriers, NPC2 proteins, are also present with few genes in insects, from just a couple to about a dozen, but are much better represented in some Crustacea and Chelicerata (Pelosi et al. 2014). Such differences in the repertoire of carrier proteins seem not to be related to what we would imagine should be the requirements of the species. For example, we would predict that honeybees needed a complex and rich repertoire of chemosensors to recognize the large variety of floral scents and environmental odors, as well all the different pheromones regulating their social life. On the contrary, a coleopteran, like *Tribolium castaneum*, spending most of its life within stored grains, might survive with a less sophisticated olfactory system. However, honeybees possess only 21 OBPs and 6 CSPs (Forêt and Maleszka 2006), as compared with the 50 OBPs and 20 CSPs of *T. castaneum* (Dippel et al. 2014). These large differences are not compensated by NPC2 proteins, that are present with five and nine genes in the two species, respectively (Pelosi et al. 2014). Similarly, when we look at chemoreceptor genes, the honeybee is less equipped with 170 ORs and only 10 GRs (Robertson and Wanner 2006) than *T. castaneum*, which is endowed with 259 ORs and 220 GRs (Engsontia et al. 2008).

These large differences in the number of chemoreception genes between species have been suggested to be related to different ecology. In particular, it has been suggested that the small number of GRs in the honeybee might reflect the fact that these insects locate plants based on their smell (Robertson and Wanner 2006), whereas the large number of ORs in *T. castaneum* was required by this species before its diet became specialized and limited to stored grain (Engsontia et al. 2008).

Looking from a wider perspective, although we have witnessed a sort of proliferation of soluble carrier proteins involved in chemical communication of insects and other arthropods, only lipocalins have been associated so far with chemoreception in vertebrates. As mammals OBPs are specifically tuned to sex pheromones and act within the vomeronasal organ, the problem remains how environmental odors are detected. We might wonder whether other families of carrier proteins for general odorants are still waiting to be discovered in mammals and other vertebrates.

But another perhaps more reasonable hypothesis is that mammals and other vertebrates do not use OBPs to solubilize odorants and carry them to the endings of olfactory neurons, but these volatile molecules could directly diffuse through the olfactory mucus and reach their target receptors. This mechanism requires much longer times from the arrival of the odorant to the activation of the receptors, and in fact this is exactly what we experience with our own nose: we need times of the order of 1 s to perceive an odor, whereas insects monitor the environment and take decisions within few milliseconds, as observed with honeybees (Szyszka et al. 2014).

Perhaps the long sought and still not clarified function of OBPs and other odor-binding proteins is related to a higher sensitivity and a faster delivery of the chemical signal, as suggested by comparing olfaction in insects with pheromone and odor detection in mammals.

## Supplementary Material

Supplementary data are available at *Genome Biology and Evolution* online.

## Supplementary Material

evab091_Supplementary_Data
