# Supplementary material for: Lipocalins in Arthropod Chemical Communication
Source: Genome Biol Evol. 2021 Apr 30;13(6):evab091. doi: 10.1093/gbe/evab091 (PMC8214410; doi:10.1093/gbe/evab091)
Supplement: evab091_Supplementary_Data [file evab091_supplementary_data.pdf]

Lipocalins in arthropods chemical communication

Jiao Zhu, Alessio Iannucci, Francesca Romana Dani, Wolfgang Knoll, Paolo Pelosi

Supplementary material

Sequences of lipocalins used for the alignments and the trees of Figures 3-5

HEXAPODA

>FcanLCN_XP_021961373.1 apolipoprotein D-like [Folsomia candida]
MHRREVLVVTFLVSTLFAFSDAHTMGLGSCPRVEPLKDFNMDKFLGEWYVIQKFATSSSCMKYNFTQGTDGKLRLVQTRQ
HFLLDTIGVDHIYTYTGVLTIPDNDRSARMRVKFPLNIAGEADFLVFMTDYSSYAGIFTCQKILFGHTKSATILSRKPTL
DKAVVNQVRQKLEEEGVDPDDFSVVDQDTCRTKEDSSLNVKIDDKTFSAQNIGGVVKKVGQAVGTGIDKAGEVLGSGISK
ASDVTGDIVESFADKEDKKTAPIKSLNELLDKAKVQENEAEWLP
>FcanLCN_OXA60985.1 Apolipoprotein D [Folsomia candida]
MNLQMVKEVVLLILVGISTTIMGQVLMEGSCPPIPVMKNFSPDKFLGTWYEIERYYSDYQVNTKCNKIDFTYNPDGRIGI
LTSAIQEKSGMPKVSYGLANFEYGESARMTLSMTPTPGSPTHLEFGYWILDTDYESYAVVYSCDDFCRGRQHSKSIWVLG
RTALLPQQVIHRIYDKIKVLGLDPKRLIKVDQHQCVNVAVSVRSENSVVSNSFDRNSARKFLASLTNIFGRKKYNYF
>FcanLCN_XP_021952170.1 apolipoprotein D-like [Folsomia candida]
MESFILILSLVSIAKAQIPTFGSCPDIPVVPNFDTKFYSGKWFEIEKYFTVFEIGAICITSEYSDLGNGTLGVRNQQVQT
ISGQVRSIEGMARITDADGGAKLGVSFPTVAIAGTAPYWVVDTDYKNYSIVWSCTGFGFMSFRIVWFMSRERKPSEEIRS
RVYAKLKQLQIPTDPLQKTQQDGCTG
>FcanLCN_XP_021943265.1 apolipoprotein D-like [Folsomia candida]
MNAYIFALLLLWGEIVVSQIPLPGSCPDIPPFKNLNLEKYPGNWYEVEKYFFALEASGTCISVNYTANHDGTLNTQIRFT
ESVSGNKIAFPGKTIFLDPDTAGSGKTGNMQLTLSIINPYYLQTEVQKTYIYKLIDTDYVNYAIKWSCLPLMGVHFESVW
ILARDVTYPPEVKKLVRDSLAKSKIDTSFLQPSTC
>FcanLCN_XP_021967841.1 apolipoprotein D-like [Folsomia candida]
MFAKFYSKFILILLLGDVESQLFFHGPCPTPAPLPNINITRYSGTWYLFQTYTSSVPLFFSCQLGEYFPPTPNGTWNGVT
RMTERLTGYIISVPGTVQFLSTDGSAVFTSDNHLYSKHLENVRLNSTLSQRNFTVRIDYMVLATDYTSYAVEWTCHDFGI
SNFQFLWIFSRGGTISSNTLQNIRQVLNLQGINTAWLEDQDLSGCRFV
>FcanLCN_XP_021954634.1 uncharacterized protein LOC110851243 [Folsomia candida]
MLHFIEIFLFSCLVVRGGWGKRDNCPDVKPMKDFNIFQVLGPWFVVQYYSSAEIAFTYHCMKTEFGLSNGNMDMKMRLTY
KYAADPNEEMQTGNITWQIPDPMRPEHWRHTEDHYKGVYNTDVIDTDHSSWLILLHCSDPESEKKFLSTFILSRTPYMDK
LQMDYLREKVAAYGVNLEYLFPVNQTFCNNGEQFYAQPEETTTTTTTSSPIDEEEDQDLYFAEDSDSNNSTDGFDGEDLV
PISEDNTQGDNPSDDKLQIYPE
>FcanLCN_XP_021965912.1 apolipoprotein D-like [Folsomia candida]
MSTPLILWCLLIITIKLTEGQHPVPGRCPKVEPFKYIDVQRYGGLWYEVERFFFLPEVTGSCITVNYDPQKDGSLATTIR
LKESVTGKKLAFPGNTIFLDSTLNKNFTIGNMQLTLELFNPILPQPKFQYTYSYKLADTDYDNYIIKFGCVSLLAVHFES
VWILSRYPSYPARVERLVHNSLDRLGIDRSLLVRSKCDEIQETPKVFFPTEYHHHH
>FcanLCN_XP_021956198.1 lopap-like [Folsomia candida]
MAQAKLNNISSFISFTVLVVFGVIQLSDAQYITLGLCPRIGGMRDFNISRYEGLWYEVEKYWFAVEATASCTNVRYVANP
DGTFGVEINMLEEITRTKMSMPGVTWYRSRDGSADFMLDLLVTQSPIPFHFKYPYLVLDTDYDNYTIKYLCKQLGPFSAQ
TMWVLSREKSLSPESREHIYNFLRRVGINVQKLRRSNTSTCGDIGK
>FcanLCN_XP_021953639.1 uncharacterized protein LOC110850510 [Folsomia candida]
MCPQLVQYSGTWYLYQTYASTVQLFFSCQLGEYYPPRADGTWDGVTRMTERMTGNIISVPGTVKFFSTDGSAVFTSDNHL
FSGNLPNIGINSPAGVTLTINYIVLATDYSSYAVEWTCYDFGISNFQFLWIFSRGATISQSNLQNIRKALNSQRINSAWL
RDQDLSGCHFL
>FcanLCN_XP_021950244.1 uncharacterized protein LOC110847584 [Folsomia candida]
MRFQKFSEFVILVQLLIYALVQGQQGETCASKIEKLWPRQQLDMERFTGQWMVIQLARMNDSLVEDSFAKCVSQTISRQP
DSDNLNIRIELTLIADGTQIARFGQVNTTSDQPAIWKWSYFDNLAQFWEGVVIATDYEKYWIHYECEEVDGFRIESVVLR
YRERTVGPGQLAIYASLMEGLGFPVGNLVDIPQPADCA
>FcanLCN_XP_021956141.1 apolipoprotein D-like [Folsomia candida]
MEPKVTVGVTLLVVMVVAFSGVEGAGKTAVCNKYPPLVGFEMDRYLGRWWQMEKSPNWAELPGKCWSSFYYRDASNPEKV
KLRMDYVTRLTNAPNTFFSNLVIESPSHDPSNFMYTAPAMPWRKKHFRVLATDYDNFAIEYMCQVNMMNNVEETVWLLTR
DRRPSLEVMQQARHTLQVLGLDKVKMYEADQSCEEKPYKPNSMMDWLSPST
>FcanLCN_XP_021943487.1 uncharacterized protein LOC110842083 [Folsomia candida]
MKLIAFAIFALVLSPCFAHGEFQLSSEELDPADVEEPGCPRVTHLKNIDLRHFYGEWHLPYLSNHWIRYMAKRMHGKFMK
KSSMGRVCVKMSLKPIPDEQMMQNDNASIWIHSKCPRTGVELNLACTPSASNNAKWYCRSKDSEDHEGDSWVYVVDSDDK
TWALVVRCFPNNGMNWAIFSKVKKLDDKLVKSLLLEVEEMGFQLWNVVEIPYEKCVTKM

>OcinLCN_ODM95517 .1 Apolipoprotein D [Orchesella cincta]
MNTQASFVVVALLAAIGWNAVNAHTMGLGSCPKVEPLKDFDMDKFMGEWYVIQKFATSSSCMKYNFTKGSDDKMRLVQTR
QHFILDTIGVDHLYTYTGVLTVPDTDKASRMRVKFPLNIAGEADFVVFMTDYETYAGIFTCQKILFGHTKSASILSRQPT
LDKVIVNQIRQKLEEEGVDPDDFSVVDQANCRNKEDTSLNVKIDDKTFSAKNVAGVVKKIGGAVGSGLEKAGDVLGSGIS
KAADVTGDIVETFGDKQNQQVAPNAPTSTKDLNELLKNAKVEENDAEWLP
>OcinLCN_ODM97170 .1 Apolipoprotein D [Orchesella cincta]
MERFWFKMVSFAALIATATGQVLMEGSCPLVPVFKSFKPEKFLGTWYEIERYYSDFQVNSKCNKIDFTSNHDGRIAIVTT
ALQEKSGMPKVAYGLANFEYGDSAKMTISLTPSGASSKHRQYGYWVLDTDYESYAVVYSCEDFCRGRQHAESMWVLGRTA
VLPSQVTNRIYDRIKIMGLDPKRLIKVDQSKCVHVAVSVRSEHQGFSRNTFDRNSARKFLDSLTNVFGRSRFNFI
>OcinLCN_ODM90726 .1 Apolipoprotein D [Orchesella cincta]

MQFQAKLVIAVLAFCAVALCVEAQVPFFGGCPTKRVVEDFDASAYLGRWYEIEKYYAVFEADGKCIQAYYSDAGNGTIGV
MNTQINRKTRRMKSIQGVARFTGSESTAKFGVRFPTVPFVADAPYWVLDTDYKNYSIVWSCNDFAVANFQIVWVLARERE
PSPEIRDTIREKMDQFGIRRDLLQKTDQTNCDA
>OcinLCN_ODN05072 .1 Apolipoprotein D [Orchesella cincta]
MISIFGLIIFSIHISQNNGQILIPGSCPNLGGMQDFDLNRYVGLWYEVEKYMFLPEIAGNCVYVRYKDHTNDTFRADIVQ
VEAITRKHVVSPSRGSIISNDGSAHIDLVVDVRVPLTFVTVKIDYPYYILDTDYENYVIKWSCRPGALGHNIQSMWVLSR
SRNYLQETRNIVHDKLRELGLRPEYLLLLDNYNCPSPRESENELEEANPVMDSKTNEDMQNI
>OcinLCN_ODM91997 .1 hypothetical protein Ocin01_14686 [Orchesella cincta]
MQFLKAVILLSCSVGFIFATCPAFEYMPAINLPLHAGDWFIHGVISDSKVNSACIKVGLTSNAWGSFPVNARGTVSGKNL
SDLQPYTEVYDARISGISLPGRWTVDVDGEQYEFAILHTDYVEYSLTATCPLSANSTDTIQIQFASRTNERPMPMAEIEI
LMAKIRDSYGVTGTFQRVLQSAISCE
>OcinLCN_ODM93243 .1 Apolipoprotein D [Orchesella cincta]
MSPSSTLYLAAFAAFLSTVNGQVLLPGSCANHSVVQNFSVGDYLGAWYEIQNTYADNQIGGICTIANYSDFGNGTVRVYN
TQVNAETGELQSALGSARPLDPTKNEAKLLVNFPFMGPMESPYWVLDTDYTGYSIVWSCRPGPDNNSYQTYWFLSRTRNP
SDITRGYVQGFLTALKFDLSYLEETTQTDCPN
>OcinLCN_ODM98646 .1 Apolipoprotein D [Orchesella cincta]
MELLKSVAVLLSFVAFASASKAALCNKYPPLIGFEIDKYLGRWWQMEKTPTVSELPGKCWSSFYYRDAANPEKVKLRMDY
VTRLTNAPNTFFSQLIIDNPVNDPSNFVYSVPAMPWRKKHYRVLATDYDNFAIEYMCGVNMMGTSEETVWLLTRDRRPSL
EVMQQARHTLQVLGLDKVKMYEADQSCEEKPYKRASMMDWLVPTRR
>OcinLCN_ODM93075 .1 Apolipoprotein D [Orchesella cincta]
MRSNTLACLVVVVGLAAFGSSASLPLKLGQTRSPYGSCKSFQQYSPVHFFDVEKFSGKWYVQEQYYLNNEDNVEPQNLAC
YTYNIEKTSVDKILKLDYRYDIPNLGQGVAGVVNYTTENGVFPLESIWTMVAGDEPQVPVEPYTPLIWVQLDYESWAIAY
DCRNIETPEQSFSLETMVLYTRNATFDDQKTLDILHIVIGNYGFSSQNMGDVDNSNCDTA
>OcinLCN_ODN01228 .1 hypothetical protein Ocin01_05448 [Orchesella cincta]
MAKFSLFCVYSLLYFLCVAGQYDDDVMGNYDDPELNPADVEEPGCPKVPQLNNINLREMLGQWHLPYMSKHWIRYLAKSM
HGKLMRRSSLGRVCVKVNLKPLPEGGTPNNTDATIWVQSKCPRTGAEWNLACAPQDKLNSKWYCNDLHGRTNDGDSFVYI
ADTDGKSWALVVRCFPNNGMNWAIFSKVKELDSGLVKSLLEKVEGMGFKLSNVLEIPYEKCMTKI
>OcinLCN_ODN01229 .1 hypothetical protein Ocin01_05449 [Orchesella cincta]
MACFFQNSSFFLLMITIACFILFSPVSSEETVDQNLEGELEIDPFSVREEGCPIIIERNGLDLNRLNGSWKMPYSSLNWI
ADIGMALYNHSVKMSHIMTICPTIQIQRRASTKEDNSTWILVWKCPKLHGRF

>ApisLCN_BAH71064 .1 ACYPI003166 [Acyrthosiphon pisum]
MGLLKITLVLMVSCSSLYSVNCHSYHFGQCPTLEPMTDFSMDKLLGKWYAIQKTSTSSRCLEYNFEKTGEPNAYKLDQMS
ENSIINVAKSNNYHYVGNLKADPSLPSRMIANFPLSVAGKASFVVFSTDYTNYAGIFSCQKIPLGNRHSVTILSRSKTLD
KQYVDKVRNRIASSNVNPFDLTIVDQNNCAHLNTTMRVEINDQTFSSHNIGQAVRKVGSKIGDGVEYVIEGGKKNIQISE
>ApisLCN_XP_008181632 .1 apolipoprotein D isoform X1 [Acyrthosiphon pisum]
MSSRVFLGTFLGFTNLLDKFEDRSTLLYPRLKYLRFPIMGLLKITLVLMVSCSSLYSVNCHSYHFGQCPTLEPMTDFSMD
KLLGKWYAIQKTSTSSRCLEYNFEKTGEPNAYKLDQMSENSIINVAKSNNYHYVGNLKADPSLPSRMIANFPLSVAGKAS
FVVFSTDYTNYAGIFSCQKIPLGNRHSVTILSRSKTLDKQYVDKVRNRIASSNVNPFDLTIVDQNNCAHLNTTMRVEIND
QTFSSHNIGQAVRKVGSKIGDGVEYVIEGGKKIYKSVSSSETKDNPNSEFELMP
>ApisLCN_NP_001313591 .2 apolipoprotein D precursor [Acyrthosiphon pisum]
MVFNKKLVLLMVCMSAAMVSCQVPAIGGCPEFDSQPDFDMNRYLGTWYEAEHYVNIFEIGTRCVKTNYTKAVDGRYLVSN
EIMNRFTGVKRVLDGEIRLIVKGSDSKMNVKYTTLPISYSTEQMILETDYDSFAVVWSCSSYGVANTQNAWIMTREKLAP
GTVMQKAYGVLDKYKLSKSYFRKTDQNSCAIAEAAPNEPTSDNKWRRRRSLN
>ApisLCN_XP_008186576 .1 apolipoprotein D [Acyrthosiphon pisum]
MSVFVIFLLSLSVFVSGQSLGKCPKKETMKTFDPKEFEGQWYEVEKTFYMMEMTASCTSFNFTLNPDGTSYKVHIGTKNR
ITGNPNIFSGMATLVSKSKSVLNYQAESRLPGLFSRMIPTTGLYYIMYTDYHNYTILWSCTSFGLFHTDIIWILGRERDL
TATSRAELYNLLYELNINPDRLILAKHEKCEEFF
>ApisLCN_BAH71058 .1 ACYPI008952 [Acyrthosiphon pisum]
MVFNKKLVLLMVCMSAAMVSCQVPAIGGCPEFDSQPDFDMNRYLGTWYEAEHYVNIFEIGTRCVKTNYTKAVDGRYLVSN
EIMNRFTGVKRVLDGEIRLIVKGSDSKMNVKYTTLPISYSTEQMILETDYRFLCRRLVLLVLWEWANTQNAWIMTREKLA
PGTVMQKAYGVLDKYKLSKSYFRKTDQNSCALPEAAPNEPPRDNKGGKRRSLN
>ApisLCN_NP_001155761 .1 protein karl precursor [Acyrthosiphon pisum]
MACFKVVKVLFCIIFGFSHSTSCYKLPKNDEKCPEVRARSNCDLDAIKGPWNVIEYYASSEEVEIYRCMRSTFNLSPDTP
EISMDFTYSYADDPDNEMLTGNITWNIPSFELPGHWVHMETPYEGVYNTFVLDCKDTWAVLLHCAEKPSSPRYLSSILLS
RNRTVPLNVRSYVHDKLPKYGVQLEYTFPMVQDDCSPAVIPLYYGTKTVAAGDKKTGAGNNNFKHPMLHNDRGNKQQTDI
DTNFNI
>ApisLCN_XP_003241294 .1 uncharacterized protein LOC100568977 [Acyrthosiphon pisum]
MANSPDTVLRFKHSFLCSQVKMESLHLLILQLIIYACTAYGGPIKDEQELKPCPKIKPFNKVNIDQLLGKWFLAIMVIEA
EHEEFVEDGVCIHGELLRYNKTTLRQVWNIDNPMFIDDHSAVIELPTIENEAGIWTIQSPLGGDITATIIDADPDTHLIL
AFCGKRGSASLHMWTVVVTRQNGISAPETLRLSAILVKHGYNPMASKIISWKNCPIYTMIP
>ApisLCN_XP_029342289 .1 uncharacterized protein LOC115033041 [Acyrthosiphon pisum]
MMKKCNTLFTYLFSICLVYTYGGWKILPVDTARILAVETFGGKSHWNYVSAILRVLSNNGHHVTVFTPFPDGERDNYKEV
DTSNDFQIFQEMNLTDLLRSYTSPISIVESTRTNRMFICDSVYKSVELNKIMEEKENSNFDVLIIETLGYDCELYLASKL
NLPLIYLVSTPVIFEERFISGDIPNPAIISHLCANHAIPKKFVQIFINTALLAYSMILFSQGSKPFQILTVTVLVLENGF
LNFLVTVQERFSNYSMVSL

>TcasLCN_XP_008199365 .1 PREDICTED: apolipoprotein D [Tribolium castaneum]
MKTVHIILFVCFVAAARAQIPNLGFCPDYLPMPDFDIERFLGKWYEAERYFQFSEVATRCVVTDYAKAPSGRIYVSNEVT
NRLTGVKRVIDGSLELSGKAGEGKLNVKYSTTPIASETALTVLDTDYDSYAVIWSCSGFGPIHAQSAWVMTRERLPSGVV
LQQAYGVLDKFKISRTFFVKTDQEGCAIAASDINAANGITATSTIAEATGSEQKNANKPEEENQPVEVQN
>TcasLCN_XP_015838652 .1 PREDICTED: LOW QUALITY PROTEIN: apolipoprotein D-like [Tribolium castaneum]
MVMFDEHRTSVLFFVILFEQILGSGLGKCPKLAYMNNFNVTRFTGRWYEIERSFYLMQVVSSCVSVDLTQNSKGQLQVDV
QMKSRWSDSLTVSEGLASPSKRDPSVLVYKVVTKLPRVVGRYLPGAGAYQVIETDYDNYAVLWSCTSYGLAHTDLIFVWG
RRTEIDTRXRAHVYTVLDDLRLDSERLILPKNGNCSGNEK
>TcasLCN_XP_971031 .1 PREDICTED: apolipoprotein D [Tribolium castaneum]
MFELRVFLAGLALLAGFWGCDGHTYHLGGCPNIEPMPAFSMKKMLGIWYVVQKTSTASTCITYNFTETEEPGEYLLEQTS
QHFVLGLTPLKHEYHYTGRLSIPDDAVPGRMKVRFPLSVAGSASYTVFMTDYDTYAGIFTCQKLGFAHRQSATILSREKT
LEQIYIDKIRSKLAAANIDPFDLSIISQKNCPKGENGTNINIDDETFSAHSVAGVIKKAGEKIGDGVEYVAGGAKKVYNK

VAENIGDDKDEKSKLVTINPNAEWLP
>TcasLCN_XP_969250 .1 PREDICTED: uncharacterized protein LOC657715 isoform X1 [Tribolium castaneum]
MLRPILLLLLIASCRPYRKRPEDKTKCPSVKAIRNFNLEQMMGRWYVIQYYASSEEALSYRCMRAEFSMSPFLADVTMNF
TYSFTDDPLNELLVGNITWTVPNPGVPSHWTHSEDTYEGVYNTFVLDSDYTSWALLLHCAEKSKVPRYLSSFIMSREPVL
GVNVISYLRDKLPRYDIDLSYMFEMSQNDCNSTVIASDLPPALVAQRLPPSLRRHPMKHHH
>TcasLCN_XP_008191233 .1 PREDICTED: uncharacterized protein LOC657715 isoform X2 [Tribolium castaneum]
MLRPILLLLLIASCRPYRKRPEDKTKCPSVKAIRNFNLEQMMGRWYVIQYYASSEEALSYSFTDDPLNELLVGNITWTVP
NPGVPSHWTHSEDTYEGVYNTFVLDSDYTSWALLLHCAEKSKVPRYLSSFIMSREPVLGVNVISYLRDKLPRYDIDLSYM
FEMSQNDCNSTVIASDLPPALVAQRLPPSLRRHPMKHHH

>LdecLCN_XP_023016063 .1 This is the second part of the above protein (in red)
MFDKLVIISTVLTGIASQVPFFGRCPDIKTVQSFDVERYLGKWYEAERYFAVFEFGGKCVTADY
EVSPNGAVNVVNQQISTFTGIHSSIEGNANQISRTEEAKLTVNFPSLPVNFDAPYWIIGTDYDNYSVVWS
CNDFGIFSTLVIYGSCTYYFKPISHIECGVPEGLSI
>LdecLCN_XP_023025211.1 uncharacterized protein LOC111513262 [Leptinotarsa decemlineata]
MLGIWYVIEKTSTASSCIVYNITKTDEPEEYSIEEISQHFLLSLTPLRHGYHYRGTLTVPDSSVPAKMKV
KFPLSVAGSSSFTVFTTDYDTYAGIFTCQKITFSNRQSATILSRTRSLDKMYIDKVS
>LdecLCN_XP_023024212.1 apolipoprotein D-like [Leptinotarsa decemlineata]
MMNQIYVLAILTFTGTYAQTLLDGKCPSVNVVQNFDVEKYMGKWFEQEKYPFFHETGGKCISAEYSLNPN
GTVKVLNSKIDISSGNSSSAEGNARLASDTGEAKLLVQFPSNPVKREAPYWVLSTDYNTYSIVWSCGEMK
SSSIRFLWILTRERNPKKEIIEKAYAILDEQNISKTYLQKTNRENCPEER
>LdecLCN_XP_023019747.1 apolipoprotein D-like isoform X3 [Leptinotarsa decemlineata]
MMAMIFVLVLPILTINIISCQQIYPFLGECPKVEVMKNFNTEKFLGKWYEQEKYPIFLEMEGKCTTYQYT
TNANSTIKVAMHQVSTITKKPNNFEGDIKLDNKTGEAKLIMGIPSMNIDVPYWVLDTDYKEYAVVWSCIQ
EGKFSEWHLFSLVINIHPVK
>LdecLCN_XP_023019746.1 apolipoprotein D-like isoform X2 [Leptinotarsa decemlineata]
MMAMIFVLVLPILTINIISCQQIYPFLGECPKVEVMKNFNTEKFLGKWYEQEKYPIFLEMEGKCTTYQYT
TNANSTIKVAMHQVSTITKKPNNFEGDIKLDNKTGEAKLIMGIPSMNIDVPYWVLDTDYKEYAVVWSCIQ
EGKFSGSVGFSPVKILLGKK
>LdecLCN_XP_023019744.1 apolipoprotein D-like isoform X1 [Leptinotarsa decemlineata]
MMAMIFVLVLPILTINIISCQQIYPFLGECPKVEVMKNFNTEKFLGKWYEQEKYPIFLEMEGKCTTYQYT
TNANSTIKVAMHQVSTITKKPNNFEGDIKLDNKTGEAKLIMGIPSMNIDVPYWVLDTDYKEYAVVWSCIQ
EGKFSVRFCWILTRKNTSRKEIMERAYDVLKKQNISRRPIEKTSQKNCKAN
>LdecLCN_XP_023019743.1 apolipoprotein D-like [Leptinotarsa decemlineata]
MTSIIFSFVLAVINITYASAQIPSLGKCPPVEVVQHFNVEKYLGKWYEQEKYPTIFEIGGKCVTAEYSAN
PNGTVKVFNKQINILTGNPSSIIGNARLDSDSGEAKLLVRFPSVPFQLDAPYWVLDTDYQKYSVVWSCFE
LGPFSARVVWILTRDRQPSRDIMEKAYGVLKRQNVSKAFLMKNDQKDCPAE
>LdecLCN_XP_023029793.1 apolipoprotein D-like [Leptinotarsa decemlineata]
MLWLEEHCKVFVVIFLICLEWTSATGLGPCPKVKSMDKLNLTKFSGHWHEIERTFYLMELIRSCVTVDIS
ELSKDRLGIVVNTKSTWTGTFSISEGVASPTRRDPNIFQYKVSSSFPRIVNRYLPGAGFYQVLDTDYDTY
AVVYSCSNWQVVHSDLIWIWGRKREIDVELRSELYRKLNNMQLDPERLVLSKTTNCTDSEDFLNDDILY
>LdecLCN_XP_023019521.1 apolipoprotein D [Leptinotarsa decemlineata]
MGKYSIFHVCIIILCICESIAKRNRPEDKTKCPKVKAIRNFDLQEMLGNWFIIEYYASSEEALSYRCMRAEFSMPTQENV
NMNFTYSFTDDPMNELLIGNISWVIPNPAVPAHWTHSEDTYEGVYNTYVLDSDYTSWALLMHCAEKSKVPRYLSSFVMSR
EPSLGKNVVSYLRDKLPRYDIDLSYMFDMQQNDCNSTTDLPPSLLLNRPSLTSRRHPMKHIH

>AcerLCN_PBC29735 .1 Apolipoprotein D [Apis cerana cerana]
MQPGTKRARFCSTIIALLFLIGYNVDATWKRREDKTKCPKVKGIRNFDISEFLGSWYIVQYYASSEEALA
YRCMRAELSISPESTEVTMNFTYSFTDDPINEQLVGNITWKIPSPELPAHWVHAEYPYEGVYNTYVLDSD
YKSWALLMHCAEQSKTPRYLSSFIMSREPSLGTNVISYLREKLPRYDIDLEYMFPMDQNQCNKTEISEMD
MLIPPSVIARRNNAARKHPLKRKHRRV
>AcerLCN_PBC27513 .1 Apolipoprotein D [Apis cerana cerana]
MLRIYLILIIAASAAMAQVPFLGSCPVVETIPNFDIKKYVGKWYEIEKYFAFFEFGGKCVTAIYSEGENS
AINILNKQISALTGVSSSIEGVGKPVVKIEEAKLIVTFPTLPLPVDAPYWILDTDYTSYAVVWSCSNFGV
FSMRNVWILAREPKPPVSVLEKAYQVLDKNNISRAYFIRTDQKNCPLEN
>AcerLCN_XP_016919678 .1 apolipoprotein D-like [Apis cerana]
MKILSLAFLLGCFVLVKTHTYHVGPCPIVEPMQGFQINKFLGIWYVIQKTSTASKCITYNYTRGEEPGEY
ILKQDSDHPVLSLTSLKHEYHYTGELTIPNPSTPALMKVRFPLSVAGSASHVVFATDYDNYAGVFTCQKL
TFAHRQSATILSRNRELDKTSIDNLRQKLSDFGVNPFDLSIISQIKCSHGNNSLDITVDPSTFTSQNIGN
LVRKAGEKVGDGVEWVANMGSKVYHKLTGSEEKITSKPEENTEKNLMRHYEETNEVEWIP
>AcerLCN_XP_016917943 .1 apolipoprotein D-like [Apis cerana]
MFGKIVLFLSALALAGAQIPSLGFCPEYVPMANFDMTKFLGVWYEAERYFQLTEVVSRCVMANYTLGADG
RFRVSNEVTNRFTGIKRVLEGEIKKAASKAEEGKLVVKYTIPLTPETKYSVLETDYESYAVLWSCSGIGP
FHTQNAWVMTRERLAPGTVLQKAYAVLDKYKISKTFFVKTNQEDCALLDAKPTQEPQVEGGGEKPEDVRS
AVAPDAPQAILNSKKNGNNEERPAHPPENPSEKKPAANTVPERIMEVADAVKGEEGKEATTKGEEGRVEG
NVERADEEKKKKKL

>Mrot_XP_003704885 .1 PREDICTED: apolipoprotein D [Megachile rotundata]
MQPGTSCSRFCSLIVPLLLLACLIDDTAGTWKRREDKTKCPKVKGIRNFDISEFLGSWYIVQYYASSEEALAYRCMRAEL
SLSSDSTEVTMNFTYSFTDDPINEQLVGNITWKIPSTELPAHWVHAEYPYEGVYNTYVLDSDYKSWALLMHCAEQSKSPR
YLSSFIMSREPSLGVNVISYLREKLPRYDIDLEYMFPMDQNQCNQTDTSEMDMLIPPSVLARKNNSGRKHPLKRKHRHV
>Mrot_XP_003703611 .1 PREDICTED: apolipoprotein D-like isoform X1 [Megachile rotundata]
MGTMKCLIFLAGCFVFAKAHTYHMGGCPIVEPMQGFQMSRFLGVWYVIQKTSTASKCITYNYTRGEEPGEYVITQDSDHP
MLGLTPLKHEYHYTGELSVPEPSTPGRMQVRFPLSVAGSASHVVFLTDYDNYAGIFTCQKLAFAHRQSATILSRHRELSK
VYVDQIRERLSSFGVDPFDLSIVPQTGCPRNNDTFDINVNPNTFTSESLGNVVRKAGEKLGDGVQWVANTGSKVYHKLAG
DEQNTTKPEDKAGVGTYKEVDTNEVGWIP
>Mrot_XP_003699771 .1 PREDICTED: apolipoprotein D-like isoform X2 [Megachile rotundata]
MLYLSLILMMVSVTMAQVPFLGVCPNVETMPNFDVNKYAGKWYEVERYFAVFEFGGKCVTATYTMNDNGTIGILNKQISA
ITGVSSSIEGTAKPIGKSDDPKLVVTFPSIPLPLDAPYWVLDTDYETYSVVWSCTNFGVFSVRNAWILTREPKPPVPVLE

KAYQVLDKNNISRAYFIRTDQKNCPKGN
>Mrot_XP_003699772 .1 PREDICTED: apolipoprotein D-like [Megachile rotundata]
MIGKIVFILSALGLATAQIPSLGFCPDYVPMANFDMNKFLGVWYEAERYFQLTEVVSRCVMTNYTVGPDGKFKVSNQVTN
RFTGIKRVLEGEIKKAASKAEEGKLTVKYTIPLTPETKYSVLETDYDTYAVLWSCSGIGPFHTQNAWVMTRERLAPGTVL
QKAYAVLDKYKISKTFFVKTDQEDCVFLETPVKPVPAAAAPSTETKPQESPETNENVRSAIVPDAPQIIIDTQSDESSKP
QVETAQKKLVSVPERIVEVAESAKENLEKKEEKEATKEKADEKVKDAVKTA
>Mrot_XP_003703508 .1 PREDICTED: apolipoprotein D isoform X2 [Megachile rotundata]
MKILYCLVFLAGCVIFAKAHTYHMGACPIVEPMQGFQMNKFLGIWYVIQKTTTASKCITYNYTRGEEPGEYIITQDSDHP
VLGLTPLKHEYHYTGKLTVLDPSTAARMQVHFPLSIAGSASHVVFLTDYENYAGIFTCQKLAFAHRQSATILSRRRELDK
SYVDKIRERLSYYGVDPFDLSIISQTGCPRGNNTLDINIDPNTFTAENFGNAVRKAGEKLGDGVQWVASAGSKVYHKLAG
SEEKSTTKPEENTRAAINKDYETNEVEWIP

>NvitLCN_XP_001607285 .1 apolipoprotein D [Nasonia vitripennis]
MIWLYLGFFLVGSAVAQVPFLGGCPKVETMPNFNTQKYLGEWYEAERYFAFFEFGGKCVTATYQANDNGTVSVVNRQISS
LSGVASSIKGFAAQKSPERSKLSVVFPSLPVHFDAPYWILDTDYTSYAVVWSCNDFGLFSTRNAWILTREQHPAVATVEK
AYQVLDKNQISRAFFVRTDQKNCPQQSQSTTESSESNQIS
>NvitLCN_NP_001153453 .1 apolipoprotein D-like precursor [Nasonia vitripennis]
MKSIAKLLLIAALAITARAQIPSLGFCPEYLPMTGFDMERFSGIWYEAERYFQLTEVASRCVMANYTKGPDGKFHVVNEV
TSRFTGIKRVLEGEIRKAPSKAEEGKLHVKYTTVPLIPLETQYNVLETDYDTYAVLWSCSGVGPVHTQNAWIMTRQRLAP
GEVLQKAYGVLDKYKISKVFFVKTNQDDCAYLDSLQAEQAAKPTEPPKKNQTPQVAEAEEEQEEEDQPKPLAGKSADFRS
EHITPDAPEAIIKTRKAEIAAKEKLQEITEPIVEAVVEAAEPAVAAPEENAVKSVPEVILKIADQAKELAKEPAQEPSIA
TQEAAITEPIKEEPAAAIADEPAAPVIAEEPKEAVPAILEEEKKPAPAAIIEEEKKIEPEPEKIIEPIIEEKIEIVKA
>NvitLCN_XP_016839918 .1 apolipoprotein D isoform X2 [Nasonia vitripennis]
MRSLTLCLVLAGCVAAALAHSYHLGACPVVEPLAGFQMSRFLGIWYVVQKTSTASKCITYNYTRGDEPGEYSITQDSDIP
ILGLTSLKHEYHYTGALSVPEPSVPARMTVRFPLSVAGSASHVVFATDYENYAGIFTCQKLAFAHRQSATILSRTRDLDK
AYIDKVRAKLSSFGVDPFDLSIITQTNCPRGNNSLDVNVDPHTFSAENIGSVVRKAGEKIGDGVEWIGHQGSKVYHKIAG
TEDKSSERPATVVPVNSQQQPPKDAAGKYENNEV
>NvitLCN_XP_001602517 .1 apolipoprotein D [Nasonia vitripennis]
MRGCNLVLLLAIAIVTVCSLADALAVGGWKRREDKTKCPKVKGIRNFDISQFLGVWYIVQYYASSEEAISYRCM
RAEMSVLPENDEVTMNFTYSFTDDPLNEQLVGNITWKIPSPELPAHWMHAELPYEGVYNTYVLDSDYKSWALLMHCAEKS
KSPRYLSSFIMSREASLGNNVISYLREKLPRYDIDLEYMFPMDQTNCTMIDPYANSFVPPTIVAAKRNYMQRRHPLKRKH
KRG
>NvitLCN_XP_031779660 .1 uncharacterized protein LOC103317102 [Nasonia vitripennis]
MSRCRRITLVALAALATCSIVCEAQTANFEGTKIVDLNQFVGDWYIVASTPASGTSVNKCGHFVVKKLSENTFAMKYTAV
SHKRNSPVVFNANGTVGDDVTETWQLEGSTKFIGPIKQIIIEGNYDSYLATVLSGEKTANFANHRIAMIWSRDKRLSSFV
VDKLKRKLSQYANKKDIFTIDQSSC

>DmelLCN_NP_001285137.1 karl, isoform C [Drosophila melanogaster]
MERIMGRNGSLLLTAVLIIILDVLLAPVGANLWTRHNSNAYQTRRRSGPSNRCPKVGAIKNFDLERMMGCWHVVQYYAST
EELPEYACMRSHFSFSKEDQHITMNFSYIFAEDPLREKLVGNITWMIPKFQEPGHWQHTEDIYEGIYNTYVLDTDYDTWG
LVMHCAEKKKQPRYLSALLLSRKTSLADNEISFLRGKLPQDIDTSFMFNIGQESCDNLMESSRDDPLAYVVNGRKRAKEI
FKIINKPDGRVRASSEARSYEQSEKL
>DmelLCN_AAF85707.1 neural Lazarillo [Drosophila melanogaster]
MNHHSSSHLLLLISVVFGAVWVAHAQVPFPGKCPDVKLLDTFDAEAYMGVWYEYAAYPFAFEIGKKCIYANYSLIDNSTV
SVVNAAINRFTGQPSNVTGQAKVLGPGQLAVAFYPTQPLTKANYLVLGTDYESYAVVYSCTSVTPLANFKIVWILTRQRE
PSAEAVDAARKILEDNDVSQAFLIDTVQKNCPRLDGNGTGLTGEDGLDVDDFVSTTVPNAIEKA
>DmelLCN_AAF85708.1 glial Lazarillo [Drosophila melanogaster]
MMSGQPLGSRVWLLSGVLLVTSAGTDAYGFGRCPNYPSMPKFNMSRVLGHWYEVERSFYLPEIASGCTTFQFEPYNKGEQ
SKFSNSKLAVAIKNINRITGNPNVNIGYATPENSRSSIMDFKFTTRFPDVIARLLPGSGKYQVLYTDYENFAILWSCGSI
GSLGHSDQIWILGRDRDFEVDIRSKVYDVLKRLSLDPERLIISKNKQCPEAL
>DmelLCN_NP_001162852.1 uncharacterized protein Dmel_CG31659, isoform B [Drosophila melanogaster]
MAMRAFHGACPSNMTAVGDLDMDRFKGKWYTHSIYPHLSLRVEKCQSTDFIEKEENKFSVVARELNTQTGTVKMRKADIL
NVEPEFGRYVLGTTSTAFPEGVLMYVLDTDYVNFAIRFMCFDASKIFSFHWAVIQTRKRLPSTQVIHMAQYFGKSAGLVI
GDMSKVPQESCPYDT

>AgamLCN_XP_320076 .4 AGAP009281-PA, partial [Anopheles gambiae str. PEST]
MATIRIPRWAGIGVGLLAVLLLLGVEAQVPGFGKCPKVPVVENFDTYAYLGRWYEQEKYPFFFELGGKCITADYSLNPDGTIGVLNTQKNSITGNEN
SIVGSARIVQSARLAVRFPSAPFNVEAPYWVVGTDYKTFAVVYACSDLRGFINAKVAWILTRKRHPDIETMKKAYSVLDSAKISRAYLTRTDQKNC
>AgamLCN_XP_320077 .4 AGAP009282-PA, partial [Anopheles gambiae str. PEST]
MNGAPGWACRVVGVLALACLVTVVHTQIPGFGTCPDYSPILRFNRTRFLGTWYEIERYFTVTEVATKCVSVTYEQRADGK
IYVRNAYTNRFNGVERIISGVMDKGGKSKEGRYQIEYTSFPYNYNATVMVLDTDYDSFAVLYSCSSFGPVGHAVSAWMMA
RERLPAGPVLQRAYGVLDKYKISRTFFIRTQQEDCVTLPPPEPAIDPTEPSTQAARNEGVIVQSEEDLQQLRSDIFAV
>AgamLCN_XP_003436167 .1 AGAP013229-PA [Anopheles gambiae str. PEST]
MLKQLVCGALFLATLVQGTIFERPCRTDVAVVQDFQVDQYLGLWYDLEHYEASFEQNTDCVTAEYSRYADGSIRVFNSAV
RLTDGLLYAVDGLALLSYPEAEVLEAKLNVSFYGAPNDESNYWILDTDYENYSIVWSCEPIGEERSLEYYWLLSRTPALP
EDEELREKIEILKEQNGIIDDELIITEHFAEG
>AgamLCN_XP_563568 .1 AGAP002593-PA [Anopheles gambiae str. PEST]
MSFVRCLFVVLVAAGSIALAQRIVSQPCPDPANRPVVQYFDLQRYVDGRWYEISRYDQHFEKDCDCGYATYTPLADGSVR
VQNCCERLPNTTVKCSIGKAVVSYPDVFPLEGRFNVTFGGPPKTSNYWILDTDYDNYALIYYCKNLSESKSAEAAWVLSK
QRTIHPSVQDTVNGLVDKYFVRQDMRITEQSQAKCKYDTNDV
>AgamLCN_XP_003436166 .1 AGAP013451-PA [Anopheles gambiae str. PEST]
MQTVALSVAIVCLLAGAVYGGLMFDRPCPTNVPVKQYFEVDSYLGKWYEMQRYESEFEENFDCVQVRYTLNEDDSVQVSN
SAYNLFNGSSINALGRAVLSFPDEEVVQGKLNVSFFGAPNDLSNYWVLDTDYETFSVVWNCQPRGEEQSEESFWVLSRTP
TLPADTDVLFRIHYIMRRYIDRSLVRFTSQLDERYVVNVKVVFGFWIGF
>AgamLCN_XP_563569 .2 AGAP002592-PA [Anopheles gambiae str. PEST]
MISKTVNMLALAVLIVSSLQVAVGFVVRDGNCTLATANLPFVKDFQLEQYLGKWYELERYEQDYERNMECVSIVYRWQQP
LETLDVNYRGYLPHNGTVNTFTGSGVFSQEPAQETANSTAPTTAAKLLVSFGRVYNATNYWVVDTDYVNYAIVYSCVTFA
EMGQAVEGYWLLARTPNLPDNQTVIERVKYLRSTYFQVSHMRFTNHTEELCPEEEKLPTEPSLVILPPL
>AgamLCN_XP_001689062 .1 AGAP011478-PA [Anopheles gambiae str. PEST]

MAKVPRVLAMTATVVALACLAGVVQSHTYKTGDCPSVEPMSGFSMKQFLGIWYVIQKTGTASTCVIYNITKNPDTPDEYF
IEQISQKAPLSIAPIKHEYSYTGKLTVTDRDVPARMTVRFPLSVAGSAKFVIFMTDYDTYAGVFSCQKIPFGHRQSATLL
SRTRDLDKIYVDKIRTRLASYSVDPFDLSIVNQTGCPKEGEAGWNIHIDPDTFSTRNIANAFRKAGEAIGDGFEAAVNAG
KKIYNKYRDGDSDEMEAEVVETGTHRSERLINAAEWLP
>AgamLCN_XP_312344 .3 AGAP002594-PA [Anopheles gambiae str. PEST]
MKTFPCLMIAMAYLWCAVDGSQGGLLTLSCMTFEEDYNFKEDKFDGTWYEVRRLSDPSATQHEDCVVMNYKLGEQGSFEI
RQSYQVGDESEPIYRSGRAEPKVFQDARIPKFFERFNTTDPADPDISIDIVATDYSSYAVVYSCTSINSTHHLESAWVLS
RQPALAKNVVELVNLFLESRFTRPDHKWRATIHTADYCKPTSVEELPMYAGAGTRTALSMPAVLLGMLLAVLLH
>AgamLCN_XP_003436165.1 AGAP013318-PA [Anopheles gambiae str. PEST]
MNSIQLLAAAFVLGLVGLTSGQ
LVSPGVCQDFPVHVNFDVPRYLGLWYEIRRYEQVFQRGGECVTAEYSLNDDGSVRVFNSMLVPPGQVRQSDVGRAVVAFPDESPLEAKLNVTFDATA
TDISANYWVLGTDYDSYAVVWGCFGVGTTLRAESAWILSRTPTLTPQAEAEVQRYVDLYLSEEDLRPTVQNLDFCCTIDPEVTAYPQCPTQ

>AaegLCN_EAT40277.1 AAEL007972-PA [Aedes aegypti]
MVEDFKCKVLLFVVVALCGFAGLVNSQIPGLGGCPDYVPITKFDRNRFLGTWYEVERYFTVSEVAAKCVS
ATYELMPDGKVYVRNALVNRFNNVERIISGVMQPAGKSKIGQYDVLYQSFPYNYNASFMVLDTDYDNFAV
IYSCSTIGPVGHTVSAWVLARERLPPGPTLQRAYGVLDKYRINRTFFVKTIQEDCVVRPPPQPAIDPTEP
SVSRNRNHAPSDQIETEEQLIQLRKDLFAVPSLNYQDIQIEPVDNDDNE
>AaegLCN_EAT38566.1 AAEL009567-PA [Aedes aegypti]
MRFIYRLELLIAAVLCLAFVIEALIISSGTCPKRPVVRNFNVSRYTGLWYEISRYEQPFQLGGECVTAQY
SLNKDGTVRVFNSMLIPPNDVRSSIVGRAVVSYPNKDPVPAKLLVTFNGVPVASNYWVLDTDYDHFSVVW
SCFQIGGIIHTQGAWILSREPELSDSIKHRVQEAIDRYLEESHLRKTNHNYELCCSSQPDIQSYPEC
>AaegLCN_EAT38565.1 AAEL009559-PA [Aedes aegypti]
MKSVIAVVFCLSIGSSLAQIISIGQCSSPAVVQDFDVEAYLGLWYEVSRYEQTFQRNGECVTAEYSLNAD
GSVRVQNRMLVPPSGQFDEDIGRAVISFPQEDPLQAKLNVSFGGMPPIKSNYWVLDTDYTSFAFVWSCFP
VSDNIKGESYWLLSRTVELPASVQARVDELTDAHLIRRHIRQTRHDLFYCNGPEETPEVAESPKSQAKAE
DLRSKRIIL
>AaegLCN_EAT38564.1 AAEL009566-PA [Aedes aegypti]
MKSSVLLQIVSLVTLLAVSSVSAEVYYDRPCRTDVETVQGFCLDRYLGKWFEIERYEQEFETNLDCTQAQ
YGLIDPSTVSVMNSAYSLMNDTAIVAIGTAKLSFPEDELVPAKLNVSFFGSPNDRSNYWVLDTDYENFSV
VWSCESLPENRSRESYWLLSRTRQLTQDEQVMERVNSLVEKYIDPEEIRVTNQSDDRCPVF
>AaegLCN_EAT38561.1 AAEL009560-PA [Aedes aegypti]
MSVLMTLLSATLLLMTSTGIVCQRIVQQPCPDPETRPIVKNFDLERYISGRWYEILRYDQYFEKDCDCGY
ATYTIKRLNTLKVENCCERLPNTSTHCSIGKAVVSFPNAVPLEAKLNVTFGGPPNNSNYWIMDTDYDNYA
IIYSCKNLSDNKSAEAAWVLSKQRTIKADVRSKVDQLVDQYLVRADMRVTEQSQSICKYEEMPAKN
>AaegLCN_EAT38559.1 AAEL009561-PA [Aedes aegypti]
MRSLLVFLATFTVFLSKTLSINVQGPCRDLPVENQFNITQYMGTWYEIKRYENEYQPNGDCVTAQYTLNS
TTMEVTVENTMKKLPDQKPSVARGRAVLAHPTSGEAKLLVRFESTPEAAPSSIYWVLKTDYQKYAVVWSC
HAVGENSTESAWILSRSPVMEQASEVVVENLIKQHLKPESFRNTKQGDEFCSGAPAAKISCVLLILFVLL
ICKKS
>AaegLCN_EAT38558.1 AAEL009569-PA [Aedes aegypti]
MKSLAIVAFLLFGTILSINAVIYEKHCLIFEESYNFDESRYAGKWYEIRRLYDPNDVELEDCVQEQYTQA
EDDKLNFDILRAVQEGPTGEVIYSTGTATPKVFRNSKVPQFIVRYNTTDPADPDTAMDIVQTDYLNYAIV
YSCNPVNTTTVSEFAWIISREPVLKKHTADLINKFVDAHFNHPEHKWRTTEQSDKTCKPNTLPPSSAAIR
TTLYSSLVQITVALLVAKILL
>AaegLCN_EAT36662.1 AAEL011278-PC [Aedes aegypti]
MKLRPTILICTVLLIVQHLKMVHSSGFGKCPNYPTMPKFNMTRFLGKWYEVERSFYLPEIASGCTTLTFE
DTTIRDSDGRPQLEIAIKTVNRWTGNPSISIGEAITENEKSSIMSVQLKSRLPTAVARFLPGSGKYQVLY
TNYDDFAILWSCSSFVAVHADQMWLLGRERDYSAEIRKKIYSALKQLSLDPERLFISKNTNCPNTL
>AaegLCN_EAT34157.1 AAEL013574-PA [Aedes aegypti]
MAKFLAMWAICAIVCGLITLGSGHTYKTGECPTVEPMSGFNMQQFLGVWYVIQKTGTASSCVIYNVTKTD
EPGEYDIEQVSQRNPLSVGPLKHEYSYTGKLTVTDKDVPARMTVRFPLSVAGSAKFVVFMSDYNTFAGVY
SCQKIPFGHRQSATILSRTRELDRIYVDKIRNRLSSFSVDPFDLSIIDQTGCPKEGEAGFNIHIDSNTFS
TKNIANVFRKAGEKIGDGFEYAVNAGKKVTHLF
>AaegLCN_XP_001660231.2 apolipoprotein D [Aedes aegypti]
MESLTLVQIVSLVTLLAVSSVSAEVYYDRPCRTDVETVQGFCLDRYLGKWFEIERYEQEFETNLDCTQAQ
YGLIDPSTVSVMNSAYSLMNDTAIVAIGTAKLSFPEDELVPAKLNVSFFGAPNDRSNYWVLDTDYENFSV
VWSCETLPENRSRESYWLLSRTRQLTQDEQVMERVNSLVEKYIDPEEIRVTNQSDDRCLVF
>AaegLCN_XP_001660226.2 lazarillo protein [Aedes aegypti]
MRSLLVFLATFTVFLSKTLSINVQGPCRDLPVENQFNITQYMGTWYEIKRYENEFQPNGDCVTAQYTLNT
TNMEVTVENTMKKLPDQKPSVARGRAVLANPTSAEAKLLVRFESTPETVPSSIYWVLKTDYQKYAVVWSC
HAAGENSTESAWILSRSPVMEQASELVVENLIKQHLKPESFRNTKQGDEFCSGATAAKISSVLLFLFVLL
ICKKY
>AaegLCN_XP_001660228.2 apolipoprotein D isoform X2 [Aedes aegypti]
MSVLMTLLSATLLLMTSTGIVCQRIVQQPCPDPETRPIVKNFDLERYISGRWYEILRYDQYFEKDCDCGY
ATYTIKRLNTLKVENCCERLPNTSIHCSIGKAVVSFPNAVPLEAKLNVTFGGPPNNSNYWIMDTDYDNYA
IIYSCKNLSDNKSAEAAWVLSKQRTIKADVRSKVDQLVDQYLVRADMRVTEQSQSICKYEEMPAKN
>AaegLCN_XP_001660229.2 outer membrane lipoprotein Blc isoform X1 [Aedes aegypti]
MLRYSLLMVAMVILSASNTCGYIVKDGNCSVVSSSMPVVTNFEIEKYLGKWYEIERYEQDFQRNLECVTA
EYTQSKLDESLDVKSKGFLASKDAYASFAGIAVMSDPLSNPAVGKLNVTYGIKANGISNYWIVDTDYEKY
AVVYSCTPIEDSESVIEGYWLLSRTPALTDEPQIMDKLQYLQSNYFVPSHVRPTNQSESLCRKEPEIPPV
PVVDVLPPLP
>AaegLCN_XP_001660232.2 apolipoprotein D [Aedes aegypti]
MKSVIVVSVAVVFYLSIGSSLAQIISIGQCSSPAVVQDFDVEAYLGLWYEVSRYEQTFQRNGECVTAEYS
LNDDGSVRVQNRMLVPPSGQFDEDIGRAVLSFPQEDPLQAKLNVSFGGMPPIKSNYWVLDTDYTSFAFVW
SCFPVSDNIKGESYWLLSRTVELPTSVQARVDELTDAYLIRRHIRQTRHDLFYCNAPEETPEVAESPKSQ
AKAEDLRSKRIIL
>AaegLCN_XP_021709994.1 apolipoprotein D [Aedes aegypti]
MWAICAIVCGLITLGSGHTYKTGECPTVEPMSGFNMQQFLGVWYVIQKTGTASSCVIYNVTKTDEPGEYD

IEQVSQRNPLSVGPLKHEYSYTGKLTVTDKDVPARMTVRFPLSVAGSAKFVVFMSDYNTFAGVYSCQKIP
FGHRQSATILSRTRELDRIYVDKIRNRLSSFSVDPFDLSIIDQTGCPKEGEAGFNIHIDSNTFSTKNIAN
VFRKAGEKIGDGFEYAVNAGKKIYHQYTDSGSTEQGSPTASPSSGHRVEKLVMPQNPDAEWLP
>AaegLCN_XP_001660225.2 apolipoprotein D [Aedes aegypti]
MKSLAIVAFLLFGTIHSINAVIYEKHCLIFEESYNFDESRYAGKWYEIRRLYDPNDVELEDCVQEQYTQA
EDDKLNFDILRAVQEGPTGEVIYSTGTATPKVFRNSKVPQFIVRYNTTDPADPDTSMDVVQTDYLNYAIV
YSCNPVNTTTVSEFAWIISREPVLKKHTADLINKFVDVHFNHPEHKWRTTEQSDKTCKPNTLPPSSAAIR
TTLYSSLVQITVALLVAKMLL
>AaegLCN_XP_001660233.2 apolipoprotein D [Aedes aegypti]
MRFIYRLELLIAAVLCLAFVIEALIISSGTCPKRPVVRNFNVSRYTGLWYEISRYEQPFQLGGECVTAQY
SLNKDGTVRVFNSMLIPPNDVRSSIVGRAVVSYPNKDPVPAKLIVTFNGVPVASNYWVLDTDYDNFSVVW
SCFQIGGIIHTQGAWILSREPELSDSIKHRVQEAIDRYLEESHLRKTNHNYELCCSSQPDIQSYPEC
>AaegLCN_XP_021703438.1 apolipoprotein D [Aedes aegypti]
MVENFKYKVLLFTVVSLCGFAGLVDSQIPGLGGCPDYVPITKFDRNRFLGTWYEVERYFTVSEVAAKCVS
ATYELMPDGKVYVRNALVNRFNNVERIISGVMQPAGKSKIGQYDVLYQSFPYNYNASFMVLDTDYDNFAV
IYSCSTIGPVGHTVSAWVLARERLPPGPTLQRAYGVLDKYRINRTFFVKTIQEDCVVRPPPQPAIDPTEP
SVSRNRNHAPSDQIETEEQLIQLRKDLFAVPSLNYQDIQIEPVDNDDNE
>AaegLCN_XP_021699910.1 apolipoprotein D [Aedes aegypti]
MKLLPTILICTVLLIVQHLKMVHSSGFGKCPNYPTMPKFNMTRFLGKWYEVERSFYLPEIASGCTTLTFE
DTTIRDSDGRPQLEIAIKTVNRWTGNPSISIGEAITENEKSSIMSVQLKSRLPTAVARFLPGSGKYQVLY
TNYDDFAILWSCSSFVAVHADQMWLLGRERDYSAEIRKKIYSALKQLSLDPERLFISKNTNCPNTL
>AaegLCN_XP_021697582.1 uncharacterized protein LOC5574999 isoform X1 [Aedes aegypti]
MLILIVGQCDSQMGRRRNRNERCPRVRAMRNFD
LPSMMGYWYVIQYFASSETLPEYSCMQSSFITTDGLITMNFTYFFSDDPLRNFQQGNITWVIPNFAQPAH
WIHAEWTYEEIYNTYVIDTDYQSWGLIMHCAEKTKSQKYLSALMLSRTPTLTQNVINFLREKLPRYDIDL
SCMFPISQVNCSHREVDPKAYFKYESV

>CquiLCN_XP_001863795 .1 conserved hypothetical protein [Culex quinquefasciatus]
MNLHLTLVPLLLAAFTQIANAQIVALGACPKVPQVADFNPQRYGGLWYEQEKYPFIFELGGKCVTAEYSL
SADNTITVVNRQISTIQLAVPIILLASSLLSIEAQIASLGICPDVPKQHDFHPERFVGLWYEQERYPNVF
ELAAKCVTSEYSMNADTTFTITNKQINSLTGNESKYFGSARRTKISKYQVKFPFACE
>CquiLCN_XP_001851241 .1 hyphantrin [Culex quinquefasciatus]
MDLKCRILICLVGLAVLSVVNCQIPGFGGCPDYTPILRFNRTRFLGTWYEVERYFTVSEVATKCVSATYE
LQPDGKIYVRNALTNRFNNVQRVISGVMEAQGRTKDGRYTIQYQSFPYNYNATFMVLDTDYDSFAVIYSC
SSIGPVGHTASTWVLARERLPPGPVMQRAYGVLDKFRINRSFFVKTIQEDCVIRAPPEPAYDPTESSTGS
RRHSVPGLKGENEDEYSVLKTEDELVQLRNEFVVMPEIDVEVDPVVEESD
>CquiLCN_EDS26941 .1 apolipoprotein D [Culex quinquefasciatus]
MTTFTGVWYVIQKTGTASTCVIYNITKGEDPGEYDIEQRSQRAPLSVGPFKHEYSYTGKLTATDRDVPAR
MTARFPLSVAGSAKFVVFMSDFETYAGVFSCQKIPLGHRQSATILSRTRDLDKIYVDKIRNRLASFSVDP
FDLSIINQTGCPKEGEAG
>CquiLCN_XP_001870653 .1 karl [Culex quinquefasciatus]
MRNFDLPAMMGNWYVIQYFASSETLPEYSCMRSSFTTTDGFVTMNFTYYYSDDPLRNFQQGNITWVIPNF
AQPAHWIHAEFTCKNSRKVQFALNITSKSKISDEEIYNTYVIDTDYKSWGLIMHCAEKTKAQKYLSALML
SRTPDLTQNVVNFLREKLPRYDIDLSTMFGIPQGNCSALEGEQDPKKYFKYETL
>CquiLCN_XP_001869618 .1 apolipoprotein D [Culex quinquefasciatus]
MMVKFPLLFTVLVLLGTVTVNAVLYDRPCRTDVPVVQNFALTRYLGKWYELQRFEKDFQTNYDCVQTEYG
LLDPTTVSVRNSAYSLVNETSIEAIGTAKFSFPEQDIVQAKLNVSFFGAPNDRSNYWVIDTDYEHFSIVW
ACEQLGEDRSSEGYWFLSRTRRFTDDVEANTRAFHAIRQYIDRTEIRFTNQLDERCPDF
>CquiLCN_XP_001866789 .1 apolipoprotein D [Culex quinquefasciatus]
MYKSPQVFAAALLVLGLSSAVVYGVIYDRPCRTEISVVQNFCLDRYLGKWYELQRYEQPFQTKVDCTTAN
YGLLDSATVSVRNSAFSLINGTSSEAIGTAVLSFPEQEIVQAKLNVSFFGAPNDRSNYWVIDTDYENFAI
VWSCEPLPQDQSSEGFWFLSRERKFTDDKDANERAFGAIRKYIDQSEIRFTNQADERCPDF
>CquiLCN_XP_001868835 .1 karl [Culex quinquefasciatus]
MLVLIVGQCSPQMAGGGRRRNRSERCPRVRAMRNFDLPAMMGNWYVIQYFASSETLPEYSCMRSSFTTTDGFVTMNFTYYYSDDPLRNFQQGNITWV
IPNFAQPAHWIHAEFTYEEIYNTYVIDTDYKSWGLIMHCAEKTKAQKYLSALMLSRTPDLTQNVVNFLREKLPRYDIDLSTMFGIPQGNCSALEGEQ
DPKKYFKYETL
>CquiLCN_XP_001866793 .1 apolipoprotein D [Culex quinquefasciatus]
MQLTAVSILLVCLVLPGFRALDVAGPCRTLPVVQSFDVTQYMGMWYEIQRYPHRNQPNGDCVVVNYTLNQ
TTGEVAILNRMRVIADDATVEARGTAVTDPDYPGQGVLRVRFEETPPEVPASYYRILGVVYDRYAVVWSC
RQNGANSVESAWVLSRAPTLEGVSVSNVKAIIDQHLDQESFTITKQGEQYCGGAAALTTSIVVFILSVFV
MLQSNN
>CquiLCN_XP_001866792 .1 apolipoprotein D [Culex quinquefasciatus]
MSVSKIKMLLPLVLALMLLTSQPVVSQRIVEQPCPDPESRPIVQNFDLDRYVAGKWYEILRYDQHFERGC
DCGFATLTPKKSGSIKVENCCERLPNTTLSCSVGRAFVSFPDHVPLEGKLNVTFGGPPLNSNYWIMDTDY
DNYAIIYSCKNLSENKSAEAAWVLSKQRTLEPRIVPTVDKLVEQYLVRADMRTTEQSQSKYVW
>CquiLCN_XP_001866791 .1 conserved hypothetical protein [Culex quinquefasciatus]
MVLLIRMNLPSLQYLGKWYEIERYEQDYERNLECVTAEYTRNVQDGSIDVKNKGFLAKKDAYASFSGIAY
ISDPLLDPVVAKLNVSYGILASGISNYWVVDTDYRHFAVVYSCTPIDDSESVIEGYWLLSRMPKLTEELQ
ITEKLRYLLETYFVPSHVRPTNHSEALCRKEPEIPPVPASLVLPPLP
>CquiLCN_XP_001866788 .1 apolipoprotein D [Culex quinquefasciatus]
MKSLVVLSIVLATVATTCRAQIISLGQCAVPDVVENFNVSAYLGKWYEIERYEQVFQRNGECVTATYSLN
DDGSVRVENAMLVPPSGKFDIDIGRALLSFPDEDPLRAKLNVSFGGMPPIASNYWVLDTDYESFAFVFSC
FPVGNTLKGDNYWLLSRTPELSPEVRDRVEVLIDLYLNRRHIRRTRHNLY
>CquiLCN_XP_001863495 .1 apolipoprotein D [Culex quinquefasciatus]
MKSSTIAIGLLLAMIATSSSVMAVIYEKYCIRFEESYTFSEDRFVGKWYEIRRLEDPIHDQDEHCVQEKF
TRSANMMDFDIVRSVQANASAEPVYSSGVASPRVLASAVVPQFYLRYNTTSPADPDTPIDIVKTDYNSYA
ILYSCLQINSTTVAENAWIYSRRLDIPKPTAELINKFIATKFNHPEHKWGTTVQSSPFCKPTNITNSSHG
LHQLSLLWRSLAVLLLAKMLF

>BmorLCN_XP_004932390 .1 apolipoprotein D isoform X2 [Bombyx mori]
MMFQMSILRAIAVFFSVLMFVSLCGSQIIMPGQCPDVKAMENFDPARYLGKWYEAEKYFFLFEFGGKCVTADYKLRDDGA
IRVLNKQIDIFSGIQKEIKGEATQVGRSDEAKLSVRFPTLPVDVAAPYWVVDTDYDNYAVVWSCYEFGIFHTVNSWILTR
QQNPPKSVLDAAYDAIDKNRISRKFFLKTDQSDCTNFDE
>BmorLCN_XP_004932389 .1 apolipoprotein D isoform X1 [Bombyx mori]
MMPLQFQMSILRAIAVFFSVLMFVSLCGSQIIMPGQCPDVKAMENFDPARYLGKWYEAEKYFFLFEFGGKCVTADYKLRD
DGAIRVLNKQIDIFSGIQKEIKGEATQVGRSDEAKLSVRFPTLPVDVAAPYWVVDTDYDNYAVVWSCYEFGIFHTVNSWI
LTRQQNPPKSVLDAAYDAIDKNRISRKFFLKTDQSDCTNFDE
>BmorLCN_NP_001140192 .1 32 kDa apolipoprotein precursor [Bombyx mori]
MWRLTVLVLAATASAQIPSLGWCPDFQSMANFNMNRFLGTWYEAERFFTVSELGSRCVTTNYVSTPEGRIIVSNEIVNSL
TGMKRLMEGSLQMIGREGEGRFMIKYSSLPLPYESEFSILDTDYDNYAVMWSCSGIGPVHTQNTWLLTRERLPSLMAMQN
AYAVLDRFKISRTFFVKTNQADCTILPDPVAIPIEAKSADVIKNVDIKVKEKEPVEDSDSVKKQIIDEVVQERSAVPEIS
FEPKPVPVPEMILTENEKKGENMEEPKAEDKAEAVEPKAVETTTI
>BmorLCN_ANU05020 .1 RFP, partial [Bombyx mori]
TTAINTIVNRINVLDARYYQTVDRSDAACFYYPEPTGQPVVFRGQCDTTIPVVPNFNANAYMGLWHEIERYPTPFQEGTC
ANARYSLTGGTVDVINTEVINQRLESINGFAVLATTDGSAKLKVTFPVAGTTQTIETDYWVLSTDYTSYSLVYSCRNLDS
ERRQVISWKLSRTKQLTNAAATTIRTVMNNINVLDQRYFSQTDQTPAGCFYFPEPRPGVQVEFPGQCETTIPVVPNFNMA
QFQGIWHEIEAYPKDDQPGQCVNHQFTSGTGNTLNLVSSNVLNQALGITRGVVSFASNDLAG
>BmorLCN_NP_001036872 .1 Bombyrin precursor [Bombyx mori]
MLRLVLLTLLAAATAEVIHEGTCPELKPVNNFNLTAYQGIWYEISKFPNESEKNGKCSSAEYKLEGDVVKVKNVHIIDGV
KKYIEGTAKLTDDANKAAKLTVTFKFGEISRDGSVQVLATDYNNYAIAYNCKYDDKKKSHQVFVWILSRNKKLEGDAKTA
VDNFIKEHSKEIDSSKLVHTDFSEEACKFTISSVITEHGKH
>BmorLCN_XP_004921596 .1 apolipoprotein D [Bombyx mori]
MSGRELIAAYKIWLLFGALAEVVTGTGFGRCPAYPSLPNFDAQRMTGIWYEVERSFYLVEIAASCTRLNVTLNDRGYFQI
TVDSVNRWTGSQSTSYGIGIPSYNGSSVFRYKLNNRMPYLIGRLLPGAGQYNILFTDYDQFALVWSCSSVSLAHSDRIWV
LGRKQEIEADVRVQIYAIMQELRLDPDRLFISKNTNCTDSLEAE
>BmorLCN_XP_004923379 .1 apolipoprotein D [Bombyx mori]
MNVVNLFLVVVLVNTSGGQVLQFGQCPEVETMEYFDLVRFLGRWFEIERFPAWFEDKGHCAYKRFQYCNRKVEIEQVFIR
DGIRFIIHRNSSYNPGDEAIFDIQPSNIDPVGIPLSVISTDYNNYAILFGCKFNVQMDMKYILAWILSREQSLPPEVLNE
TRRKLNSIPFANAAYLMPVNHTEYNCNYHWTAHIQSVYKTVDEN
>BmorLCN_XP_004926879 .1 apolipoprotein D [Bombyx mori]
MWKLTVLGVFLTVTYVYSHTYHLGSCPIVEPMSGFEMNRLLGVWYVIQKTSTASHCITYNFSRTDEPGQYQLEQDSQHFI
LGLTPLKHDYKYTGVLTVPDPAVPARMKVRFPLSVAGSASYTVLATDYTTYAAVFTCQKLAFAHRRSATILSRTKELDKM
FVDKMRLKLSSYGVDPYDLSIISQNDCPGLPDGASEGVNIDINPETFSSHNIGEAVRKTGGAIADGVEYVVEAGKKVYHK
VASSKEDLTEPPSNPYAVRPMDSDAEWLP
>BmorLCN_XP_004923537 .1 uncharacterized protein LOC101739113 [Bombyx mori]
MKMVKFLMFIVFLLFSNVVIGKIVNKRKCPEVRAVPHFDLPGILGDWYVVEYYASAEEALSYSCMKAVFTQDDHVPAIDG
TIEWTPGVTMNFTYRFADDPIGETLFGNITWKIDLNQPAHWTHAESTYDGIYNTYVIDTEYKTWALLLHCAEQTEGARYL
TAFVLSRTTKLQKNVMAYLRDKLPRFEVDINYVFAIPHDDCAVKPPNPNFGPLMLDAQSKKLIRPSVSYKVFGH
>BmorLCN_XP_004923378 .1 insecticyanin-A [Bombyx mori]
MLIKHLIFAASFCVVHAYFTLPGKCPADVQLQQEFRLADFFGKWYQAYHYSSDEQQQNNCSVLELQTKPSGIYLNQSRID
RGLFHRYSIAKLEIPSNLEDAAKLNVKFFFENAPRRLRIRRYPFSVLATNYQYYATVYTCQYSPLTDKHFIYIWILSRNP
ILNDVSKELATKPLSQLGIDVTKIKKDDLTRCTPKYYEDSQTEPMTFRYPVPV
>BmorLCN_XP_012553141 .2 lopap [Bombyx mori]
MLLFLLCISLYGASGAEFTLPGECPNMKLQENLDYTKFSGVWYNVASYASDGRPIYDCATLDFQEDNLGYTLRETYVNVD
GGNRTQKSYLARVDPTFDAGNKAQFIVSHEGGDKVLQFPFFILSTDYDEYAIAYTCKTLKKKTRTHYVFTWILTRTKNKL
QGDTQKKVETALSNYLELAEHRKQFVFKDFSEEDCSYTDQFKTDFFTSNFW
>BmorLCN_XP_004923380 .3 uncharacterized protein LOC101745601 [Bombyx mori]
MLVYSLVFFLIVNSDAVVRNETCPVVTPRELNWKDMDGTWYVAAVATDMQVQGDCAMIIFDHQETPDVSIRWITNNTASF
YNGSVALTPDPNGNSTGGDLLLVTYNDNKTETYSFLDINYEHYAVVFACYNNDDGSSTYEIWKLTRTPHLKDTDAVKLDQ
AIANYSLQGTPFINFNNTEDTCRINAGRQLDASTLIMTSAAAITLLRRMF

>HarmLCN_AFK64814 .1 apolipoprotein D-like protein [Helicoverpa armigera]
MFRFLILAFVATAFADTHDGPCPEVKPVENFNLTAYQGVWYEISKLPIASEGKGKCGQAEYTLNGDEVKV
KNSHVLDGEQKFIEGTAKFAADANNAAKLLVSFKFGEIVSESGLQILTTDYHSYSIAYNCKYDEKTKTHN
EQAWILSRSKSLEGEAKATVDAFLKEHSKVIDSTKLVKTDFSEEACKFTGSSVTTEQTAKP
>HarmLCN_XP_021200319 .1 apolipoprotein D-like [Helicoverpa armigera]
MSVLRAIAVFLCGFLSLRLCRAQIIMPGSCPDMKAMDNFDAARYMGKWYEAEKYFFVFEFGGKCITADYS
LKENGVVGVVNRQINILSGTQSEIQGQATQVSRSDEAKLAVSFPSLPVNVEAPYWVIDTDYDSYAVVWSC
YEFGLFHTVNAWILTRERNPPVSVLEKAYSVLDKNQISRAFLIRTNQKDCDGATS
>HarmLCN_XP_021200364 .1 apolipoprotein D-like isoform X1 [Helicoverpa armigera]
MMWRLLLLAFVATASAQIPSLGWCPDFQAMANFNMNRFLGTWYEAERYFTVTELGTRCVTTHYTATPEGR
ILVTNEITNSLTGFKRIMEGHLQKVGREGEGRVMVKYSSAPLPYDFEYSILDTDYDSYAVMWSCSGIGPV
HTQNTWLLSRDRLPSLAVMQNAYAVLDRYKISRTFFLKTNQADCTVLPEPAAASELDAKSADVPVPAPVP
VEEKAKEVEQPVEKRVDPEVKAVVPEERSAVPEPVEEASKAIQVPELIMSESEKKEEKIEPAPVPAPEEP
AVVAETKEMKETPAQ
>HarmLCN_XP_021182455 .1 apolipoprotein D-like [Helicoverpa armigera]
MYSLSAIGVLLAICGGAFSHTYHLGACPVVEPMPGFEMNKLLGVWYVIQKTSTASHCMTYNFTRTQEPGT
YELEQVSQHFILGLTPLKHDYRYTGVLTVPDPAVPARMKVRFPLSVAGSASYTVLMTDYTSHAVIFTCQK
LAFAHRRSATILSRTKELDKMYIDKMRNKLSSFGVDPYDLSIISQSQCPSHTPDEGVNINIDPETFSSHN
IGQAVRKTGTAIADGVEYVVEAGKTVYNKVASSKEDLTEAPAGRAYSVKDDAEWLP
>HarmLCN_XP_021185371 .1 apolipoprotein D-like [Helicoverpa armigera]
MGAAGVTGFRIYCVLFCTIIKAVISTGLGKCPVYPPLPNFDIQRMTGTWYEVERSFYLVEITASCTELDI
ALNERGYLLITINTVNKWTNSPSTHHGIGIPSHNTSSIFRYKLNNRVPYIIGRLLPGAGVYNILFTDYDQ
FAIVWSCTSVSIAHSDHIWVLGRRREIEAPVRAQIYAIMQELRLDPDRLIISKNNNCTDIRDNKI
>HarmLCN_XP_021190587 .1 insecticyanin-B-like [Helicoverpa armigera]
MLAQCLVLALAVFRVQGAMMNMSCPDVTPRELDWKALDGIWYLTAIATEDMQVQGDCATVLFDHQNTTDV
SISWITNNTVTYYNGSVALIPDPNSNSTGDLLLVTYDDDKTETYSFLDVNYEHYAVIFACYNNDDGNSST
YEIWKLTRTPHLKDTDAEKLDKAIANYSLQDTQFFTFNNTEDTCRVNSGHQLDTSTLVMTSAAALALFRR

FY
>HarmLCN_XP_021190592 .1 lopap-like [Helicoverpa armigera]
MCRQLFLVNFLVVVYSALCGSQVLQFGACPEIDTMKYFDIEEFQGRWYEILRFPIWYEQYGDCAYKRIQY
CGRRLEIEHVFVKDGVQFVLHLNTTYVPGDDAIFVIRESNIDPVGIPMNVVLTDYDNYAIVYGCKYSEAM
GLKYISAWILSRRTTLPEEIMAQVNHELRSLPYANTAYFEEVDQSNEKCASHWTAHVQAVNYNGDL
>HarmLCN_XP_021190608 .1 insecticyanin-A-like [Helicoverpa armigera]
MLILIFSMCFVAAFGAEYTLLGECPDVKLQENLDHTKFAGLWYTVASYASDGRDIYDCSTLEFLEDNLGY
MFKETYVQLDQGNRTQKTYQARVDPTFDAGNKAQFIMSHEAGDKVLQFPFFILSTDYEGYAIAYTCKWLK
EKIRKHYVFTWVLTRGKEKLQGELLKKVENTLSKYTDLAEHRQSFVFKDFSDASCAYTKKFETDFFTSNF
W

>DpleLCN_XP_032529327 .1 apolipoprotein D-like [Danaus plexippus plexippus]
MMSVIRAIAVFLCGLSWTVCKAQILFPGACPDVAAMSDFDPSRYLGKWYEAEKYFAAFELGGACITANYKLKDNGAISVV
NEQFSLLTGTKKSITGEAVQVSRSEPAKLSVTFSSLPVNIPAPYWIVSTDYDSYALIWSCYDFGIFHTRNAWILTRQRKP
SASVLDEAYSAADKNNINRSYFMRTDQTNCSEDS
>DpleLCN_XP_032529061 .1 apolipoprotein D-like [Danaus plexippus plexippus]
MLSLVLLLAATASAQIPSLGWCPDYQPMADFNVNRFLGTWYESERYFTVSELGSRCVAIKYNSTPEGRILVSNEITNSLT
GLKRVLEGSMQMIGREGEGRMMIKYTALPRTYDNEFSILDTDYDNYAVMWSCSGIGPVHIQNAWVLTRERIPPLMVLQSA
YSALERFRISRTFFVKTDQADCNVLPDIAADPSLLKSTIEVSIDAKNVHPIPNVEIPIEKKEESKIPPPIVRSAIPEISD
EPMKMQEIEMMDKKEEKIEKPVEMKKENMEMEKEKEKEKMADIEKPQ
>DpleLCN_XP_032514158 .1 apolipoprotein D-like [Danaus plexippus plexippus]
MGDFLVMGIKLKIILLCTAVKTVTSTGLGKCPIYPQFPDYDINRMTGTWYEVERSFHLMEISASCIQLDVTINERGFLLI
TVNTINRWTNTPSISYGLGIPSHNGSSSFRYKLNNRMPYIIGRMLPGAGLYNVLFTDYERFAIIWSCTNYSIAHADRLWI
LGRHRSLAANTRAIVYALVTALGMDPDRLLLTKNNNCTSTDLTTDYD
>DpleLCN_XP_032521562 .1 bilin-binding protein-like [Danaus plexippus plexippus]
MSGIGIDCCTGTHNDKVKMYAVVFLALVASALAEVYYVDSRCPDIQPVQNFDVANYGKGVWYEIARYPNRKEMKIDCGHTE
YTPQGDHFSVKNLGFNNGKLMSIEGVAKLAEDAGNSGKLIFTLPYGASGKKTDNVLNVLYTDYDNFAIVYHCQFHEEKNS
RQDFAWILSRSKSLSPELKAKVDKFVSESKVLDSSKFVWPDFSDKACKASA
>DpleLCN_XP_032521318 .1 insecticyanin-A-like [Danaus plexippus plexippus]
MWTIFCFCFYVTYVLGAEFTLPGECPTVKIQENLDYTKFSGVWYNVASYASDGRSIYDCASLSFQEDNLGYTLRETYVDI
DQGNRTQKSYFARVDPTFDAGTKAQFIVSHEDGDKVLQFPFFILSTDYSGYAIAYTCKTLKKKQRTHYVFTWVLSRKKEK
LQDETLKNVENVLAKYSELAEHRQSFVLKDFSESSCAYSNKYETDFFTSNFW
>DpleLCN_XP_032521565 .1 lopap-like [Danaus plexippus plexippus]
MFFNVLLLVLSLHLCSGQTILFGACENVETMKYFELERFLGKWYEIERIPSWYDDDAMCSYKIIQRCGRRIEFQHGFVKD
SIEYVLHVNSTYTPGAEAVFDIQKNNIDPFGIPLSIITTDYTNYAIMYGCKVNPELNLKYVLAWVLSRNKTLSSKLLEEA
HQRLMTSLAYFHPVSHSEKLCDHMWSAHVHAHHHEADNDSLAET
>DpleLCN_XP_032527239 .1 apolipoprotein D-like [Danaus plexippus plexippus]
MYLVSIYLLLALCGSAISHTYHLGACPVVEPMPGFDMNQMLGVWYVIQKTSTASHCITYNFTRTDEPGRYELEQLSQHFI
LGLTPLNHDYRYTGILTVPDPAVPARMKVNFPLSVAGSASYTIMATDYTNYAAIFTCQKLAFAHRRSATILSRHKELDKM
YVDKMRAKLSSFGVDPYDLSIISQTECPKHPNGTSNGVNINIDPETFSSHNIGEAVRQTGTVIADGVEYVVDAGKKVYNK
VTSSKEDLTESPDKPVRSLKDDAEWLP
>DpleLCN_OWR41648 .1 biliverdin binding protein-I, partial [Danaus plexippus plexippus]
MKYFELERFLGKWYEIERIPSWYDDDAMCSYKIIQRCGRRIEFQHGFVKDSIEYVLHVNSTYTPGAEAVFDIQKNNIDPF
GIPLSIITTDYTNYAIMYGCKIVSLAYYQSTDNVSCNPKISNSRLGVIKKQDIVIKTIRGSSSK
>DpleLCN_XP_032516144 .1 lopap [Danaus plexippus plexippus]
MMRALSFLFLFSFLICTVYSRIVDKRKCPEVRAVPHFDLSQMLGDWFVVEYYASAEEALSYSCMRAVFTEDDHVRAEDGS
IEWTPGVTMNFTYRFADDPIGENLLGNITWKVDLNEPAHWTHSEITYDGIYNTYVLDTEYKTWMLLLHCAEQSEGARYLS
AFVLSRKTTLPKNVMAYLRDKLPRYDVDIKYVFPISHEDCSDKPAKSDFSPILVEVGSKTPKRPGVSYNVAFGH
>DpleLCN_XP_032521324 .1 bilin-binding protein-like [Danaus plexippus plexippus]
MYTLLLLTLFSTAYANVFFDSKCPEVKSAENFEFSKFGKGDWYEIARYPIDLEKDSKCVKVIYTWMDDHAMVKCILMVND
KQVELNGIMRPVVKAGNTEKLFCSVSNENVKLETDVYILDIDYDNYAIAYGCKYDEEKKSRQDFAWIMSRSNTLSPAIKA
KVENFVKESEFLYNDKFFWPETSCVAN
>DpleLCN_XP_032521317 .1 insecticyanin-A-like [Danaus plexippus plexippus]
MWQTIRNETVASSVAMFKLIPFFLCVNSVFGYFTLSGKCPDSVKLQENFKLADFYGKWYQAYHYSSDDQHNNNCSTLELL
TRPSGIYLNQSRVELGLFHRYSVGKVEIPQREEDASSLYLVFAFKNAPRRLKIRRYPFHVLATNYNYYATIYSCEYSPLI
DKHFIYVWILSRHPILNDVSKQLASKPLELIGLDSTKLWKDDMSKCTPKYYEDFPAEPTTFRFPVPI
>DpleLCN_XP_032521325 .1 bilin-binding protein-like [Danaus plexippus plexippus]
MFALVLLALVAQAFAGEYIDARCPVVRPVDNFDYASFSNGIWYEVGRYDNRKEKGTECSYSIFTPMGDRFKIENIGLRPS
FSSENGQNTDNILNIMSTDNANYAVLYHCNYNEETKTKQVLCQFNISNKIMRTKQTIHRATKIIELARFQIVSRGARLDD
GSD
>DpleLCN_OWR46183 .1 bilin-binding protein [Danaus plexippus plexippus]
MRIQMHISPHTSTMFALVLLALVAQAFAGEYIDARCPVVRPVDNFDYASFSNGIWYEVGRYDNRKEKGTECSYSIFTPMG
DRFKIENIGLRPSFSSENELSWIVSRTKTLRSDFKALIDRYLLESKVLDLSKYIFPDFSDNVCRIDF
>**DpleLCN**_XP_032521323 .1 bilin-binding protein-like [Danaus plexippus plexippus]
MFALLILSLVSTAYAGVFFDSKCPEVKSLDNFSFSKYGESEWFEVARYPNDQEKGFKCIRSSYSFKGDFAEIKS
FLVKDGKEYQLEGTLKKAEDGQKLISNLQYDSIKIENSVYLLDSDYENYSIAYSCKYDEEKKSRQDFVWVASRSLPMSSD
IKAKVENFMKASSFLDYGKLIWHENDCSNLK
>DpleLCN_OWR45930 .1 bilin-binding protein [Danaus plexippus plexippus]
MFALLILSLVSTAYAGVFFDSKCPEVKSIDNFILLKSGESEWFEVARYPNDKAKGSKCIRSIYSFKGDYAEVKTFFVRDG
KEYQLEGTLKQTESGGKFISDLQYGSTKIVNNVYLLDSDYENYSITYSCKYDEEKKSRQDFVWVASRSLPMSSDIKAKVE
NFMKASSFLDYDKLIWHENDCSNLK

>PxutLCN_KPI91793 .1 Apolipoprotein D [Papilio xuthus]
MSVLRAIAVFLCGFLSLRICRAQIVFPGTCPDVEAMKNFDAERYLGRWYEVEKYFAFFELGGRCITADYGQKDDLITVTN
KQINNITGSTNEIKGYATKESGEADEAKLSVYFPKMPINIAAPYWIVGTDYDSYSVIWSCYEFGTLFNTRNAWILTRQRN
PPKSVLKAAYKVTKENNIDQSYFTKTDQTNCLQDED
>PxutLCN_XP_013176156 .1 PREDICTED: apolipoprotein D-like [Papilio xuthus]
MSVLRAIAVFLCGFLSLRICRAQIVFPGTCPDVEAMKNFDAERYLGRWYEVEKYFAFFELGGRCITADYGQKDDLITVTN
KQINNITGSTNEIKGYATKESGEADEAKLSVYFPKMPINIAAPYWIVGTDYDSYSVIWSCYEFGTLFNTRNAWILTRQRN

PPKSVLKAAYKVTKENNIDQSYFTKTDQTNCTVIEDED
>PxutLCN_XP_013176033 .1 PREDICTED: apolipoprotein D-like isoform X1 [Papilio xuthus]
MWRLFFLIAAVTAQIPSLGFCPDYQPMASFNMNRFLGTWYEAERYFTVSELGSRCVTTKYESTPEGRILVSNEITNAMTG
MKRVLDGHLQMIGREGEGRMIVKYATLPVPYDTEFSVLDTDYDTYAVMWSCSGIGPVHIQNAWVLTRDRLAPQMTMQKAY
AALEKFKVSRTFFVKTNQEDCYILPDPAAYSEYKEAGIEVSGKHASLVPVPAATDAEPVVEKKVDAEVKPEVPEVKSAVP
EVINEPQVVPEIKAAETMAKEEKTEMPEQTTEVKKEMPEIKEEAPKEKDLPVKPIQ
>PxutLCN_XP_013181969 .1 PREDICTED: apolipoprotein D-like [Papilio xuthus]
MGDFRTCAATTLYLLLLASVARHVLPTGLGQCPTFGQLQDFDINKMVGKWYEVERSFYLMELSASCTELRVKLNNRGQLD
IVIHTRNRWTGTHCITRGMGVMSHDGASSFRYRVHNRMPYVIGRLLPGAGQYSILATDYDHFALIWSCTGLSLAHSDRMW
VLGRKREIDAQLRAYIYNLLNNFGMDADRLILSKNNDCPDYDKNNTRTD
>PxutLCN_NP_001298747 .1 bilin-binding protein-like precursor [Papilio xuthus]
MFRFVTIAVLFAAATSEVIFEGPCPDIKTVDNFEFEAYGGTWYEMAKYPNAGEENTKGKCTIAEYTVNGDKGKVKNSHVI
DAVRHYISGDLTLVAPGKIMLTYTFGGQSKNSYLNILDTDYKSYSIGYSCKYFKDGNKHQVFAWIKSRSKKLDCEAKYKI
DNFLRTSKVLDSAKFVVNEHTDAACSAPTTKTITEFLK
>PxutLCN_NP_001299264 .1 insecticyanin-B-like precursor [Papilio xuthus]
MFRFLVIAFLAVAAADVVIVDGPCPDVKPMESFKFSDYQGTWYEVAKFPNTGEDGKKGKCTTAEYTIDGDKGKVKNSQVV
DGVKSYVDGDIALIAPGKVRITYKFEEFTKNSVLTVLDTDYKNYAIGYSCKFLDKENKHQVFSWILSRTKSLGDSQAKVD
AFLANFPAIDKSNYVFNDFSEETCKFTSTKAITSIRKKN
>PxutLCN_XP_013170913 .1 PREDICTED: bilin-binding protein-like [Papilio xuthus]
MHCYILFALIVVASASSDVIYQGPCPSVKPQQNFDFASYQGTWYEIARYPNAGEEGTRGKCTIAEYFIHGYDSGRVKNSH
VVDGVRSFIEGDLTLVGPARIRLTYTFDGRSKYSYLTVLDTDYVNYAIGYSCKYFPDGNNHQVFSWIKSRNPTMDPYSRA
IVENYLKKSTILDANKYIANDFSSAACQTYIFRTITEFLKD
>PxutLCN_XP_013170635 .1 PREDICTED: bilin-binding protein-like [Papilio xuthus]
MHRLCLFALMAVVSADVIFDGPCPTVKPKENFDFSAYQGSWYEIARYPNAGEEGTRGKCTVAAYTVFGDSGKVRNSHVVD
GIQSFIDGDLDLVGPAQIRLTYTFGGKSKYSYLTVLDTDYQNYAIGYSCKYFPDGNNHQVFSWIKSRSPTLDAYSRAIVD
AYLKESTILDSDKYIANDFSPAACQAGVVRQITEFLKE
>PxutLCN_XP_013170971 .1 PREDICTED: lopap-like isoform X1 [Papilio xuthus]
MYARKCLTFLLFVVHIKLSRNQVLLFGPCIEVETVKFFDIEKFLGLWYEIERFPTSYEEFGQCAYKRFQACGRRIEIEHG
FIKNDILYIVHVNSTYAPGNDAIFKFKKNNIDSLGIPMSILATDYTNYAVVYGCKNNDTIDIKYISAWILSREKELSPEI
LETAHRELNKIPYASTVYLKPVLQNIEKCTHQWTAHFNAKDVIDEENNEL
>PxutLCN_KPI92168 .1 Bilin-binding protein [Papilio xuthus]
MHCYILFALIVVASASSDVIYQGPCPSVKPQQNFDFASYQGTWYEIARYPNAGEEGTRGKCTIAEYFIHGYDSGRVKNSH
VVDGVRSFIEGDLTLVGPARIRLTYTFDGRSKYSYLTVLDTDYVNYAIGYSCKYFPDGNNHQGRGSVGRPPARWIDDLVK
VAGASWMQVAQDRSLWQSLEEA
>PxutLCN_XP_013170691 .1 PREDICTED: insecticyanin-B-like [Papilio xuthus]
MHCLFVFALIAAATADVIYKGPCPNIQPKADFDFSAYQGTWYEMARYPHAGEEGVKGKCTLIEYYMFGNNSGRVKKSHVV
DNIQKYVEGNLMLAGPATMMTTLTFGGVSKNAYITVLDTDYSNYAIAYSCRYFPDGNKHQVRSWIMSRTTTLDENSRAMV
DSYLNDSRILDTNKYMGNDYSPAACQASLVKSITDLQPQYY
>PxutLCN_KPI93192 .1 Apolipoprotein D [Papilio xuthus]
MHRLTVFGLLLAFTGSAIGHTYHLGACPIVEPMSGFEMNKMLGVWYVIQKTSTASHCITYNFTRTPEPGTFEIEQVSQHF
ILGLTPLDHDYRYKGVLTVPDPAVPARMKVRFPLSVAGSASYTILATDYDQYAAIFTCQKLAFAHRRSATILSRTKELDK
IFVDKMRLKLASFGVDPYDLSIISQSECPGRPNGATEGVDINISPDTFSTHNIGQVVRKTGGVIADGVEYVVEAGKKVYH
KVTDSKEDLTETPNMHLASPNRLSAKPDDDAEWLP
>PxutLCN_XP_013170972 .1 PREDICTED: lopap-like isoform X2 [Papilio xuthus]
MYARKCLTFLLFVVHIKLSRNQVLLFGPCIEVETVKFFDIEKFLGLWYEIERFPTSYEEFGQCAYKRFQACGRRIEIEHG
FIKNDILYIVHVNSTYAPGNDAIFKFKKNNIDSLGIPMSILATDYTNYAVVYGCKNNDTIDIKYRITSRKSPI
>PxutLCN_NP_001299190.1 insecticyanin-A-like precursor [Papilio xuthus]
MLRLTLLVIFVASASAVSVIFEDGPCPQFKPMSNFDFNAYGGTWYEIAKFPNPSEEGDKAKCTLIEYFVRDFRGKMKFTH
VVDGVRKYIEGDLTQVYPGHGKIMYTYTFGGKSKNTYLYVMDTDYNNYAVAFSCRYFKDSDKHQLLSWILSRNKVLEGNA
LAAVDKFLAANSKLINTSYYVQNEHTNEACKARTTEEITEFLKIYQTNPNLID
>PxutLCN_XP_013170583 .1 PREDICTED: insecticyanin-A-like [Papilio xuthus]
MLLILSCLYLINSVLTAEFTLPGECPVVKIQENLDYTKFSGLWYNVASYASDGRPIYDCATLDFQEDNLGYTLREKYIDM
DMGNRTQKSYFARVDPTFDAGNNAQFIVSHEDGDKVLQFPFFILSTDYEGYAIAYTCKTLKKKQRTHYVFTWVLARSKDK
LQGDVLKKVEDSLSKYSELAEHRQSFVLKEFSEQNCAYTNKFEMDYFTSNFW
>PxutLCN_KPJ02409 .1 hypothetical protein RR46_08206 [Papilio xuthus]
MAKIVVLFVILFVIFDVAFARIVNKRKCPEVRAVPHFDLSQMLGDWYVVEYYASAEEALSYSCMRAVFTEDDHVQTADGS
VEWTPGVTMNFTYRFADDPLGENLLGNITWRVDLNEPAHWTHSELTYDGIYNTYVLDTEYKTWALLLHCAEQREGARYLS
AFVLSRKTTLPKNVMAYLRDKLPRVPYPIGKKRYLYRIILSSVRISVRALYLENA
>PxutLCN_XP_013173800 .1 PREDICTED: lopap [Papilio xuthus]
MAKIVVVFVILFVIFDVAFARIVNKRKCPEVRAVPHFDLSQMLGDWYVVEYYASAEEALSYSCMRAVFTEDDHVQTADGS
VEWTPGVTMNFTYRFADDPLGENLLGNITWRVDLNEPAHWTHSELTYDGIYNTYVLDTEYKTWALLLHCAEQREGARYLS
AFVLSRKTTLPKNVMAYLRDKLPRYDVDIKYVFPIPHNECTSTPAIYNYSPILVEAGSKTMKRPGISYNTN
>PxutLCN_XP_013169125 .1 PREDICTED: apolipoprotein D-like [Papilio xuthus]
MLPLIVSLFLITFASGQIARNGTCNFGNILAVRDLNVTQLSGDWYQIKRIPNAQECGTCSTTKIYATQDSFSNITISNKE
INEKKIKYRNGTIVVPYGVKNTVQGQFTITYEDIIYNSIVLATNYTNYAVVYSCKNINNSSKNVWAWVLSRNSTISKNEE
KFVQSIINGNEDLKNVAWTIPDHSKKACDPNSGITIGLSPVLILLVVAVTGKDIFSNKL

>PrapLCN_XP_022114943 .1 apolipoprotein D-like isoform X2 [Pieris rapae]
MSVLKSIVVFLVSMIVVQTSAQVVFPGGCPNIEAITNFEPARYLGTWYEAEKYFAIFELGGKCISAKYTDNGNGVIGVHN
KQVNSKGAKSGIVGEAKLTGPPNVAKLSVRFPSLPVDIPAPYWVIDTDYDNYALVWSCTNLGVMHSETSWILTRERNPPQ
SVLDTVYAAIEKNKIDKTHYIKTDQSNCPNDA
>PrapLCN_XP_022125286 .1 apolipoprotein D-like [Pieris rapae]
MGDFRVTGATRLYFILFCTIIKAVISTGMGKCPIYPQFPNFDMTRMSGTWYEVERSFHLIEIAASCTEIHTSINERGYFV
INVHTTNRWTGNPSTSYGLGIPSHTGSSAFRYKLNNRMPYVIGRMLPGAGQYNVLFTDYDNFALIWSCTNYSIAHSDRIW
VLGREREIDATLRAQIYAIMQELNLDPDRLIISKNNNCTSVPYSDTQYT
>PrapLCN_XP_022123842 .1 bilin-binding protein [Pieris rapae]
MQYLIVLALVAAASANVYHDGACPEVKPVDNFDWSNYNGKWWEVAKYPNSIEKYGKCGWAEYTPEGKSVKVKNFHVIQGK
EYFIEGTAYPVGDSKIGKIYHKLTYGGVTRENVLNILSTDNKNYIIGYYCKYDEDKKGHQDFVWVLSRSKVLTGDAKTAV
ENYLVGSPVVDSQKLVYSDFSEAACKVNN

>PrapLCN_XP_022124129 .1 apolipoprotein D-like [Pieris rapae]
MLRVCLGVLALCGVVLTHTYHLGACPVVEPMPGFDMNQMLGVWYVIQKTSTASHCITYNFTRTDEPGTYQLEQVSQHFIL
GLTPLKHDYRYTGILTVEDPSVPARMRVRFPLSVAGSASYTVLATDYTKYAAIFTCQKMSFAHRQSATILSRSKELDKMF
VDKMRTKLASFSVDPYDLSIISQNECPKHPNGTSEGVNINIDPNTFSSHNIGEAVRKTGGVIADGVEYVVDAGKKVYHKV
ASSKEDLTETPHQGRNMNDDAEWLP
>PrapLCN_XP_022123838 .1 lopap-like [Pieris rapae]
MLILFCLCLSLAYAAEFTLPGECPDVKIHEKLDYTKFSGVWYNVASYASDGRSIYDCASLDFQEDNLGYTLRETYVDFDN
GNRTQKSYFARVDPTFDAGNRAQFIVSHEDGDKVLQFPFFILSTDYDSYAIAYTCKTLKKKVRTHYVFTWVLSRNKEKLH
GDTLKNVETALSKYSELAEHRQSFVLKDFSDVTCAYDNKYETDFFTSNFW
>PrapLCN_XP_022130168 .1 uncharacterized protein LOC111003792 [Pieris rapae]
MSKLVFVLFVVVVNVVSGKIVNKWKCPEVRAAPHFDLAQMLGDWFVVEYYASAEEALSYSCMRAVFTEDDHVQAEDGTIE
WTPAVTMNFTYRYADDPIGETLYGNITWRVDLNEPAHWTHSERTYDGIYNTYVLDTEYKTWALLLHCAEQREGTRYLTAF
VLSRTTGLPKNVMAYLRDKLPRYDVDINYVFPIPHKDCSDKPARPDDSPVLVEVGSKVPLRPNVSYKISFGN
>PrapLCN_XP_022123837 .1 apolipoprotein D-like [Pieris rapae]
MYKNIVILFFFKIVQSQILQFGNCSNITTMDYFQIDKFLGKWYVIEQFPVWYEENGHCAFKVFELCERRVRIQSRYVKDK
TQYILIVNSTYYSGDDAVFQIEKNNIDPVGVPLSVITTDYANYSLLYGCRLNENLQLKYMSAWILSRSLTLTPDVLETAY
RELNAIPDASTGYLQTVDHNESKCTYQWTAEIHAVNTSTND

>RproLCN_AAQ20838 .1 lipocalin AI-7 precursor [Rhodnius prolixus]
MKTINAALFLGLVACVVGATVPKMPSGCADVHNKAVSDFNFDKFFKGQWHLTHAKLRVTTAKTCETFTVN
GDELTFTLNGQSVSCKLEKVTGARFTKFNCQMGQAKFTSYVSVLATDYDNYVLVYRCGSHEGPTKDNYLV
GQRKKVQHFLPD
>RproLCN_AAQ20828 .1 procalin-like lipocalin 2 precursor [Rhodnius prolixus]
MEKFGAVIFLGLVMSTIAQRRQETTYLDQCQSIPEKSGFKKQQFFSGDWFMTHAKDATVDTLLCYKYTTL
VNSEGKLEVQYRYFKKSEERKVICTQKDGNSQAPYIFKCVLIEGEEITYEYEVQHTIVETDGNSALLYRC
LPVGYKYTDAFLVLNRQENGAVSREVQNALSTHNLDVNKFITRKNTVQRN
>RproLCN_AAQ20827 .1 procalin-like lipocalin 1 precursor [Rhodnius prolixus]
MKTIILITIFGILMIRRIQCMQCDCESVEAAGNDGEFFKGNWQVTHSKIGAMFPICGKLETSSQDGKKII
KLDDEEVGTLEIKCTGSKESDCEVKKDNKNYRTFQSC
>RproLCN_AAQ20824 .1 triabin-like lipocalin 4 precursor [Rhodnius prolixus]
MEKLVAVTFLGLVMSTIAQQPPQRPNFLAQCQSVTEKSGFNKEQFFSGDWFVTHAKDGTESTLCRKYTTS
VNSGGKSVVQYGYNRYGKERKVSCTQKNENSQAPYIFDCEVKEDEQTVLKYEVQKTILETDGNSALLYRC
LQVGTQKKKKKKKKKFLAPPPQ
>RproLCN_AAQ20823 .1 triabin-like lipocalin 3 precursor [Rhodnius prolixus]
MKTIVALIFLGILSLVHLSSSKKCEPMNGLNSQKFFSGTWFVTHAKSGSSTILCREITFKKNDGTVESNT
KGKFQQGKSKTEYTVRCTGKESKGKVPFKCTREEGERTIKQNKEYEEEFIVAETDYNSFAVVCINKANKG
KEENILVINKKKKKKKKNFWAPPPH
>RproLCN_AAQ20822 .1 triabin-like lipocalin 2 precursor [Rhodnius prolixus]
MNRIILITIFGILTLRSIHCITNGECDAVTAQENIDEFFTGTWYVTHSKGGARASLCDQFATSSDSGGTK
LIKYSLKEDGSYGTHCEGKPSNKDNPYPYNCELKSSFLNIPAKFTVVSADNNAAVLYKCTKPTTRSADDY
FILNRQKDAEIPEEVQSTLTSLKLTSSSFTSSKDTCT
>RproLCN_AAQ20821 .1 triabin-like lipocalin 1 precursor [Rhodnius prolixus]
MKTIVALIFLGILTLAHLSSSKKCEAMKNIDSQRFFSGTWFVAHAKNGSSTILCREITLTKNGDTVKSDT
KGKFKQGKMKSEYTVHCTGKEKKGKVPFKCTREEADRSIRNNNEYKEDFTVMETDYNSFAVVCTKKEGKE
ENVLIINKNKDADFPSAAKSTLEKAELKSENLNTRKLYDCK
>RproLCN_AAQ20820 .1 lipocalin AI-6 precursor [Rhodnius prolixus]
MKTTIVVTFFGFLGCTFCKRVPTPEGCRDVYNEADPNFKLKKFFNGSWYLTHAKHQNHSVLCTKFGMTMK
PLEIKYEMGGVNVTCKGTKIKGTRRTEYVCEGNRGTPTPYTNYGATMSVIDTDYTNYATVYVCKKNGKHE
DNVFVLSRIRTGEPPEAAKQSLQKLR
>RproLCN_AAQ20819 .1 lipocalin AI-5 precursor [Rhodnius prolixus]
MKTIILITIFGILMIRRIQCMQCDCESVEAAGNDGEFFKGNWQVTHSKIGAMFPICGKLETSSQDGKKII
KLDDEEVGTLEIKCTGSKESDCEVKKDNNKITGLFRVVSTDNSNYALVYICTKPASFPKDDYLILNKDKD
AAVPAAVENKLKELKMESKDFIDKKDVCEDLKIPKIKIISL
>RproLCN_AAQ20818 .1 lipocalin AI-4 precursor [Rhodnius prolixus]
MIIIVATFLGLLGHTFAAEVTSIPTGCNALSGKIMSGFDANRFFTGDWYLTHSRDSEVPVRCEKYQTGSN
LQLNFNGKNGDVKCSGSTVSGNQGFYSFQCTTTSGGSFTSYMAVVETDYANYALLYRCGLYGSTTPKDNF
LLFNRQSSGEIPAGLSTKLNQLELTSLNKLGCS
>RproLCN_AAQ20817 .1 lipocalin AI-3 precursor [Rhodnius prolixus]
MKTIVALFMFGFLAEAQYGGPPKMSRGCKDIYNRGVDNLNYKQFFTGQWFLTHGERVSSKCDTVSVNGDK
ITFKLRGAQINCQLENVPDAKFTKFNCKKSVSKTFSTEISVLATDNNNYALVYRCGVLEDDNYKDNTVVM
QRQKQAPFPPALESEVGKFGHGLKKDSFKVLNC
>RproLCN_JAA77447 .1 putative triabin-like lipocalin 4 precursor, partial [Rhodnius prolixus]
MEKFGAVIFLGLVMSTIAEGTQSNYSLSECKSVQEKSGFNRQQFFSGDWFVTHAKAGTATTLCRKYTTSL
NSEGKLEVQYGYTQKMKTIKVSCTEKNKIDQAPFILNCIVKKGEDTEYQYEVQKTILETDGNSALLYRCL
QVGTQYIGDN
>RproLCN_JAA77441 .1 putative lipocalin ai-7 precursor, partial [Rhodnius prolixus]
MKMIIGVLLLGLVACAFGAASVPRMPQGCEDVHSKAIIDTQFKDKDFFKGKWHLTHAKRVTDPSECETFT
VNGDDVKFVYKGQNVDCKSEHVANATFTKFNCKLGGASFTSYVSVLATDYTKYALVYRC
>RproLCN_JAA77437 .1 putative lipocalin ai-6 precursor, partial [Rhodnius prolixus]
MKRIIVLTFWNSGMYICKHVRTGPEGCRDVYDQAASNLNNEQFYKGSWYVTYGKYQNHTSLCTKFDMTWT
PLQITYDVNTVKITCKGEALQSGARHTEYKCKAPEGTQHPDYESVLAVIDTDYNDYATVYTCLKSLGNGG
N
>RproLCN_JAA77429 .1 putative lipocalin ai-5 precursor, partial [Rhodnius prolixus]
MNLNMKTIILITIFGILLIRRIQCIECDCESVNDQPNIDGFFKGTWHVTHSKIGAVYPICGKFETSSQDN
NKIIKLHDDDESLEIKCKGKDCQATKDNKELTGLFRVVSTDNSNYALVYICTKATSFPK
>RproLCN_JAA77428 .1 putative triabin-like lipocalin precursor, partial [Rhodnius prolixus]
MKIIITVIFCVILTQAYFQQILNSCLEVPGMPNFDSKKYFQGTWYITHVQHGIPSSVCRTIKSSLKPDGS
LEQITYGYSLYGTLKRFYKIECNDKESSFLQKGNVSLVCLIMLDTINAEKMFHIKGVVIET
>RproLCN_JAA77426 .1 putative lipocalin precursor, partial [Rhodnius prolixus]

MKTIIAVAFLGLLGHTSCAESDTTALDSSGCRDVKTIAMQDFDPNQFFTGTWYLTHLKYRNHSSICPKFT
LTPTPPTIDYTYGVLQYPGHTFKMHCDGQTKKTDDVYTPFTCKGN
>RproLCN_JAA77422 .1 putative lipocalin precursor, partial [Rhodnius prolixus]
MKAIILVSFFGILGCTFCKHVRTGPEGCRDVYDQADPKYKRRDFYTGSWYLTHAKYRNHSTLCTKFDMTF
NPLQIKYDANMVKFTCKGKNKRRKT
>RproLCN_JAA77401.1 putative salivary lipocalin, partial [Rhodnius prolixus]
CEEIPPMTNLDSKRYFSGTWYGMYSQYGKPTSVCKTLKMTLKSDGTLNNEVYGYSLTKENKPKFASIHCN
GSENDTKGKYTLICEKVNDNKDKKKHPVTFDGVVLVTDYDRFSIVHQCLSNGTIKLGTFLVLGRKSDDYT
IQDTVKKILDPLDIKFDLFQSREKVDCKKHPDE
>RproLCN_JAA77174 .1 putative salivary lipocalin [Rhodnius prolixus]
MNSKLAYIFLIVCGLSFLQAQPNDYRKYDLRSYLPWKIGACLQPSVVENFKPKEFFKGHWYEMSSFGNFL
WQVDGICPTIEYNVTDAGEILELDYHFESSLGKYVTIRGQSYTQFIKNDRAFLPFTYKVLGGLLTFDLPT
YILGTDYETWAVVYTCKPMLFLKSEMSWILTRQRNSTDQLKPVLETLKKSHLSYGEYKPAVNVGCGPNEP
HL
>RproLCN_JAA77148 .1 putative procalin-like lipocalin 2 precursor [Rhodnius prolixus]
MNRIILITIFGILTLRSIHCITNGECDTVTAQENIDEFFTGTWYVTHSKGGARASLCDQFATSSQDGTKL
IKYSLKEDGSYGVHCESKESNKDHPYPFDCTLQSS
>RproLCN_JAA76784 .1 putative triabin-like lipocalin 4a precursor [Rhodnius prolixus]
MKIIFSLTFVAILALASAATVPDESGCQSVVGKETYEPNKYFHGKWYLTLAQKTPSSPTDVCRESKNEVL
EDGSVIHNIYAYSDKATQQFYLLECTTNLKDVKDGTSVLNCKSTKDGKVNEFQLTATVLETDYEHFTVLY
VCGKLEGKNYGNFLVLNRDKNGEPTDPKIAETLKKHGLDLSTFTSRKNVHCKDHPSTVSN
>RproLCN_JAA76746 .1 putative triabin-like lipocalin precursor [Rhodnius prolixus]
MKTIVALIFLGILTLAHLSSSKKCEAMKNIDSQRFFSGTWFVTHAKNGSSTILCREITLTKKGDTVESDT
KGKFKQGKTRTEYTVHCTGKENKGKVPFKCTREEGGRSIKQNNEYDEEFTVMATDYTNYAVVCTKKAKQG
NEENVLIINKRKDAEFPSAASDLLKKLDLKSETLKARKSFDCE
>RproLCN_JAA76723 .1 putative triabin-like lipocalin precursor [Rhodnius prolixus]
MNRIILITIFGILTLRSIHCITDGECDTVTAQENVDQFFTGTWYVTHSKVGGPASLCDQFETGLQGDTKH
IKYSLKEDGSYGIHCEGKPSNKDNPYPYDCEIKSNQRNIPAKFTVASADNNAAVLYKCTKSATFSTDDYF
VLNRQKDTDIPEDVKSKLSSLGLDSGSFTSSKSECSKT
>RproLCN_JAA76722 .1 putative triabin-like lipocalin precursor [Rhodnius prolixus]
MNRLILLTIFGILMIRRIQCITNNECETVEAAGNDEGFFSGTWYVTHSKVGGPASLCDQFGTGLQDGTKL
IKYSLKDDGSYGIHCEGKPSNKDHPFPYDCTVQSNQKNIPARFTVVSTDGNYALVYKCTKSGFFPTDDYL
VLNKQKDAEIPEGVESKLQSLGLDSSSFTSSKSECTQS
>RproLCN_JAA76720 .1 putative triabin-like lipocalin precursor [Rhodnius prolixus]
MNRIILITIFGILTLRSIHCITNGECDTVTAQENIDQFFTGTWYVTHSKVGARASLCDQFETSSQDGTKY
IKYSFDEGSYGTHCEGKPSNKDNPYPYDCKIESSQKNFPAGFTVVSIDSNYALVYKCAKSLSFPTDDYLV
LNRQKNTEIPEEVENKLSSLGLVSSSFTSSKSECTQS
>RproLCN_JAA76674 .1 putative lipocalin precursor [Rhodnius prolixus]
MKTINAALFLGLVACVVGANVPKMPAGCADVHNKAVSDFKYKDFFKGQWYLTHAKLRVTNAKNCETFTAN
GEQATFTLNGQSVSCKIEKVTGARFTKFNCQMGNNKFTSYVSVLATDYTKYALVYRCGSHEGPTKDNYLV
GQRTKVQHFLPD
>RproLCN_JAA76673 .1 putative lipocalin ai-7 precursor [Rhodnius prolixus]
MKTINAALFLGLVACVVGATVPKMPQGCADVHNKAVSDFKYDEFFKGQWYLTHAKLRVTNARNCETFTAN
GEQATFTLNGQSVSCKIEKVTGARFTKFNCQMGSNKFTSYVSVLATDYTNYALVYRCGSHEGPTKDNYLV
GQRTKVQHFLPD
>RproLCN_JAA76575 .1 putative procalin-like lipocalin precursor [Rhodnius prolixus]
MKTIVLITIFGIWMIRRIQCIQCDCESVEAAGNDAGFFKGTWQVTHSKIGAVYPICGKFETSSKDSAKII
KIEDDDGTLEIKCTGSKESDCEVTKITKNYRTFQSC
>RproLCN_JAA76564 .1 putative procalin-like lipocalin 1 precursor, partial [Rhodnius prolixus]
MKTIILITIFGILMIRRIQCVQYDCESVEAAGNDAGFFKGNWQVTHSKIGAMFPICGKLETSSQDGKKII
KLDDEEVGTLEIKCTGSKESDCEAKKDN
>RproLCN_JAA76312 .1 putative triabin-like lipocalin precursor [Rhodnius prolixus]
MKMIIVLAIFAILTFATADSQIKPKECLKVPVKQNFEPQKYFRGNWFLFNIKREEGQSNTTVCQESKSRM
LEDGTISHVIYAYSDVLKPEFIQINCTGNVKNTQEKILFQCTLRKEEVTENFQMEGTVAESDYENFAVFY
ICSKEGDGFLVLSRKEDSGPTDPRVTETLKKYGMNLEEFISRKNVKCKSHPDF
>RproLCN_JAA76307 .1 putative lipocalin precursor [Rhodnius prolixus]
MKTIIVLTFFGILGCTFCTAPETHVPTGPEHCRDVYKEADPNFKRDQFYTGSWYLTHAKYRNHSKTQCTT
FEMTSQPLQIKFDEDTLRVTCTGTVQQGARHTDYKCKAAEGSTTKYEYESIVSVVESDYKDYATVYMCMK
TGNKGDNVYVLSRTKTRQPSEAEKNSLEKLGETLEDFVKFTCT
>RproLCN_JAA76106 .1 putative salivary lipocalin [Rhodnius prolixus]
MNTVLIILGTIAFAFAEQPPTVQECLNLTAKANFEPQNYFKGVWYLSYIKYADPTGICRVSKLDTLSDGS
VQKKTYGYTESGKGTDFFHVNCNGTLKSGGAIVSFHCEQSGNEGGEPIKFEMDGKVLETDYNNFSVYYIC
YKSGENLKENFLVASRHKGGEVTDDSKIAETLKTHGQYLDKFVSNKNVICKDHPDF
>RproLCN_JAA76092 .1 putative lipocalin ai-7 precursor [Rhodnius prolixus]
MKMINAALFLGLVACVVGANVPKMPSGCADVHNKAVSNFNFDKFFKGQWHLTHAKLRVTTAKTCETFTVT
GDDLTFTLNGQQVGCKLEKVTGARFTKFNCQMGQTKFTSYVSVLATDYDNYVLVYRCGSHEGPTKDKYQI
GQRKRSTTFPSGLTSSLASKGLKQSDFNSYTCPTS
>RproLCN_JAA76091 .1 putative lipocalin precursor [Rhodnius prolixus]
MKTIIGALLLGLVACAFGAAAVPKMPEGCIDVHNKAVSDFNFNKFFSGQWHMTHAKLRVTNEKTCETFSV
NGDKVTFTLNGQSVSCKLEKVAGARFTKFNCQMGQAKFTSYVSVLATDYDNYVLVYRCGSHEGANKDNYL
IGQRTKSTTFPAGLTSSLGSKGLQKSDFSSYNCP
>RproLCN_JAA75778 .1 putative lipocalin precursor [Rhodnius prolixus]
MKTINAALFLGLVACVVGATVPKMPSGCADVHNKAVSDFNFDKFFKGQWHMTHAKLRVTNAKTCETFSVN
GDDVTFTLNGNNVKCKLEKVTGARFTKFNCQMGGKSFTSYVSVLATDYDNYVLVYRCGSHEGANKDNYLV
GQRQKLNISFRIN
>RproLCN_JAA75419 .1 putative salivary lipocalin [Rhodnius prolixus]
MKLNWAATVSWGYQLLLVIAICFFSLSSGQRPAFGRCPDVTPKKDFDVSQFTGEWNEIERSFYLFESSLS
CTKLNFTLYSNDTMVADVNYRAPWRGTSSTSEYIIKDVSKTPGILNMVLASTLPALIARMAPGSGKYIVL
DTDYIDYALIYSCTDLRLVHADFIWVLGRSKDISIDARTIIYSTLDKLKINRDRLLLSKTKDC

>RproLCN_JAA75285 .1 putative triabin-like lipocalin precursor [Rhodnius prolixus]
MEKLVAVIFLGLVMSTIAQQPPQRPNFLAQCQSVTEKSGFNKEQFFSGDWFVTHAKDGTESTLCRKYTTS
VNSGGKSVVQYGYNRYGKERKVSCTQKNENSQAPYIFDCEVKEDEQTVLKYEVQKTILETDGNSALLYRC
LQVGTQYIDTFLVLNRDAGGAVSQDVKNAISAQNVNFENLLTRTSYSCT
>RproLCN_JAA75158 .1 putative salivary lipocalin [Rhodnius prolixus]
MKELILIICTLTIAISRAQMPGFGWCPDKRAMPGFDIDKYLGTWYEAERYFTVLEAGSRCARTNYTKAVD
GRILVTNEITNRLTGIKRVLDGEIRNVPKGGVDSKISVKYSTLPFPMETEYIILDTDYDSYAVVWSCSGF
GPLVNTQTAWVMTRERLPPGTVLQKAYAVLDKNKISRTFFVRAEQEECNLGEALSEKSRASDAANQNVES
ATTSASAVNSSFHHPSVAPVETSSTGNEVYMPQKKVEGSVSSPEAVGVNSESSNNAEETVRPDSNDSSRK
PAPAVTAGPPALSSQHAVVAGSNSVVSEQTKPTVSTSEAMELKN
>RproLCN_JAA75122 .1 putative triabin-like lipocalin precursor [Rhodnius prolixus]
MNGLNSQKFFSGTWFVTHAKKGFSTILCREITFKKNDSTVESNTKGKVRERKTKTEYTVHCTGKENKGKV
PFKCTREEGERSIKQNNEYDEEFTVAETDYNSFAVVCINKANKGKEENILVINKKKDADIPSAATSTLQN
LGLNSDSLGTRKSSAC
>RproLCN_AAQ20842 .1 nitrophorin 3B [Rhodnius prolixus]
MEKFGAVIFFGLVMSTIAQAPPGPTLLDQCKSVQEKSGFNRQQFFTGVWFVTHAKDGTESTLCRKYSISE
NSDGKLVVQYGTIGNRLQIFCIEKNGNSQAPHIFNCVVKKGEQIVHIYEVQKTIVEKDGNSALLYRCLQI
GDRYIDTFLVLNRDAEGAVSQTVKDAISTQSLNFGDFLSGQVLVVKRN
>RproLCN_AAB09091 .1 salivary platelet aggregation inhibitor 2 [Rhodnius prolixus]
MMIIVATLLGLLGHTFAAAGASATTTMPKECLELKGDIKPGFDANQFFTGDWYWTHARDPKHPKLCQKYQ
ATSDLRLKFNGNSGSDVTCQGAKVIGKEGFYSFQCTTSGVTFTSFMAVVETDYNNYALLYRCGRYGSSAV
EDNFLVFNRQSSGGIPGGLTTKLSQLDLTPTSFTKLGCT
>RproLCN_AAO25746 .1 biogenic amine-binding protein [Rhodnius prolixus]
MRAYAALVLFVVALWMSEAEGASGCSTVDTVKDFNKDNFFTGSWYITHYKLGDSTLEVGDKNCTKFLHQK
TADGKIKEVFSNYNPNAKTYSYDISFAKVSDFDGNNGKYTAKNVIVEKDGRKIDERTLQVSYIDTDYSKY
SVVHVCDPAAPDYYLYAVQSRTENVKEDVKSKVEAALGKVGLKLSGLFDATTLGNKCQYDDETLQKLLKQ
SFPNYEK
>RproLCN_sp|Q6PQK2 .1|NP7_RHOPR RecName: Full=Nitrophorin-7; Short=NP7; AltName: Full=Nitrite dismutase; Flags: Precursor
MELYTALLAVTILSPSSIVGLPGECSVNVIPKKNLDKAKFFSGTWYETHYLDMDPQATEKFCFSFAPRES
GGTVKEALYHFNVDSKVSFYNTGTGPLESNGAKYTAKFNTVDKKGKEIKPADEKYSYTVTVIEAAKQSAL
IHICLQEDGKDIGDLYSVLNRNKNALPNKKIKKALNKVSLVLTKFVVTKDLDCKYDDKFLSSWQK

CRUSTACEA

>DpulLCN_EFX90456.1 hypothetical protein DAPPUDRAFT_39582 [Daphnia pulex]
MAGKISIVAALMVCIAGVVHSHTYYLGSCPRVEPVNDFDMSKFLGRWYVIQKFSTASSCWTYDFIRNKTDDSLKIVQSRD
HVALDTIGLDNNYRYTGALDVPDLNRPGFMRVRFPMSLAGKADYVVFATDYENYGAVYSCQSILFGHRRSASILSRRPTL
DQPFINKIRSKLETFGVDPHDFSIIDHTDCKTLPSTSLLNVEVNPNTFSTGNVINVAKDVGKTVG
>DpulLCN_EFX87814.1 hypothetical protein DAPPUDRAFT_306369 [Daphnia pulex]
MLSYSKLWLCLLITLGCSYRTAVDAQVYSLGSCPGVNVVSNFDVDKYTGKWYENRSYFAIFQIGLDCITAEYTKSDTGVT
VKNEGTKKILRTKSIVTGTARQLEAPNGKLGVTFASIPFAPADAPYWVLGTDYTSYAVVWSCTNRAFFNSQIAWILTREQ
FPSTDTINTALAVLATNGISQNPLKTTTQNNC
>DpulLCN_EFX87818.1 hypothetical protein DAPPUDRAFT_306367 [Daphnia pulex]
MSSSFTCLIFLLVAVGCYHSANAQVFTLAQCPTVQVVSPFDADKYLGTWYNNRNYFAIFQAGLDCITANYAKNSDNITVT
NTGFNIITRSKQVALGSARVVEPGKLIVNFPGSPSSQTANYLILDTDYESYAVVWSCSNLGPISLRFAWILTRDQVPTST
IVNKALAVFQKYAINVNKLKVTNHNNCPA
>DpulLCN_EFX82011.1 hypothetical protein DAPPUDRAFT_302799 [Daphnia pulex]
MPIHNKFLVVFVTAVQLLSPRVQSQVTFDQPCPKLDVVRDFDLHRYLGLWYEIESYPAVFSSFASCVTANYSLLADGNVR
VINRSFNTSSKSFNMVDGIARLIEPPKGQAKLGVVFPSNSFSRPVPADGNYWVLDTDYTNYSIVWSCQTFNGKSIQFLWY
LSRQRQPSESGLTFVHHRIDSFGLDQKFLKTMEQRNCPDAPASETSRPIRQQQPTRTSYYYREN
>DpulLCN_EFX87815.1 hypothetical protein DAPPUDRAFT_311900 [Daphnia pulex]
MAGCFRTIVHAQVIALSSCPNVSVVSDFEVDQYLGKWYGNRNYFTIFQSGLDCVTAEYTLDGTEIVVKNEGIQQVTRRRR
TANGKARLIEPGKLSVSFTDRLSETDIPNYFVLATDYTTYAVVWNCFDLGPISSRILWVLTREQHPSISTIDKAYAALKR
NGIDGSSLRLTNQDNCTRR
>DpulLCN_EFX87817.1 hypothetical protein DAPPUDRAFT_311898 [Daphnia pulex]
MSGFDLDQFSGLWYNNRNYYSVAQAGMDCVTTQYNNTGNVLTIKIQGKKSGSTKTLVGKGLKISDGTLTVSFFENAWMSG
AENYLVLNTDYTSYAVIYSCWPFAFGTTSLAWLLTRDQIPSNNIIDTALAVFTANNIDQTKLKIVNQENC
>DpulLCN_EFX88326.1 hypothetical protein DAPPUDRAFT_311514 [Daphnia pulex]
MHSSIVLTVLLMLIGVINCQTLNFGRCPALDVKQDFDLAKYAGKWYEYSKNRRYWIIMDSGLKCASETYTLEGDDTLLMH
NEGQRIINNRPVSVKAVGKHVAPGKLLFSFNSVTLHGTAAEDVPYWILDTDYTNYSVVWSCSENAGVNAQVTWILTREIK
PDPSVVRTAMEVLSRNGLGRTSMVTTNHRSNCRPNYRYFGYPSGFPISPF
>DpulLCN_EFX70399.1 hypothetical protein DAPPUDRAFT_328301 [Daphnia pulex]
MFINLVMLLLVMFTRSPGPVSGQILRLGHCPSFSVLKDFEMDKYLGTWYQIERYFSFPGMGGKCWTQTYYNDPEVEGRFK
LRMDYRDFVVENKMTTEMDIYQDMVDDPATLTSQLFSMPFTADDYQVLSTDYTNYAVEYRCEDRGLFQRRGCMHHYAVME
CYTMAMSYNTYVFRNHA
>DpulLCN_EFX89905.1 hypothetical protein DAPPUDRAFT_299805 [Daphnia pulex]
MANNLIQVLLLATCCIAGLNANVLVRSTRGACPTFTTKPDFDYVQYAGVWFEIEKIPVVFEEGMTCIRAIYDEIAPNTVS
VVNTAVLNGNTTAIYGSAYQPYPETQPGYLIVQFPGRPDGDYFVLDTDYVTYTAVYDCVSVGAFKLEYAWLMGRTNSLTP
EQLATARAAYTQFGIDISTFEATFHDENCVYLP
>DpulLCN_EFX87816.1 hypothetical protein DAPPUDRAFT_311899 [Daphnia pulex]
MRSTTTGTVRHVEPPNGKLNVVFSGDPTQGSANYWILGTDYTSYSVVWSCMHFGIEPINTRIAWVLTREQHPTNATIDAA
LDVLKKNGIDQSKLRLTNQHNC
>DpulLCN_EFX87819.1 hypothetical protein DAPPUDRAFT_230361 [Daphnia pulex]
MSNYIQVSIWLLIAAGCYQTLVRAQLFERGNCPEVKAFPDFNLNEYVGKWYENRKTISNFQNRIAQRCVNVQFNETDGKI
IVKNSGIQRFTRRNIVLSGHARHPDTTKGEFIVNFLGNASYADEKFVILGTDYKTYSVLWSCVDVGPTHLYNLWVHTRDP
NPSSETVEMALDVVKRNALDETKLRMTNRRNC
>DpulLCN_EFX89904.1 hypothetical protein DAPPUDRAFT_230075 [Daphnia pulex]
MSSSRFLATFLFASVVFAAGTQAAVFSRISQRAACPVVTTKPDFNYIPYAGLWYEIERFENVFQQGSTCIRAIYEEISPG
VVSVLNTGVLSDGSLTNITGSATAISPEEPGHLIVSFPGRPDGDYLVLDTDYTNYASVYSCGVAGAFVLEYAWLLGREQT
MTQEVMDVALSKFTQFGVDVSTFKMTAQGASCVYFPEYPFPPSN
>DpulLCN_EFX90319.1 hypothetical protein DAPPUDRAFT_299882 [Daphnia pulex]
MSCSLHKYSGVWFEIESYPDMMDEFDICVSLNYSMQTDGVLQVVSSWFNTSSKAHESIQGSARFVNPGLRAKLLVTYPTS
FVTESMSTDGNFWIIETDYKNYAIVFLCLPIGPNISFQFLSYLSRERFPFTMGLNFVHQRIETLGLDRTLLKPIDQTNCP
S

>PvanLCN_XP_027227665 .1 apolipoprotein D-like isoform X2 [Penaeus vannamei]
MLFASIVALVGLLGSAHAHDWGFGQCPRADPFPNLSVDKFLGLWYVIEQFDTSSTCLTLNYSRVSTTQLKVTKSRQLYLL
DSLNIDHTNDYTGTLDIPDGENAGKMRVKWPLNVAGKGDYVVYDTDYDTYAAIYECQQLSSLMHRKSASILSRTPALDQT
FVNRTKRRLNSFSIDTEDFDVVDHKSCTRRSDTDLSIKIDEDTFKKIMSGASEGIKNVASTVADGASNLADTVVDVTRRL
GAQTDGTNASAAIRADPDVELIS
>PvanLCN_XP_027230074 .1 apolipoprotein D-like [Penaeus vannamei]
MKMQSSVVFLVLVAAASIEGHKFKFGNCKDYSPVANFQPGKMNGTWYVIKKVATTSTCMSVIYNNTANYMEMREIRTPAS
ITPFNIILTNIGKLTQKGNNPAVMNLKWENVISNVLKTTYTVVDTDYTSYAIDAECQSLYFARRESATILSRTPTMDPEL
VKELEKKLVNEGIDTKSLNPIDQSNCFEPQEVDYNIVVDEDGVRAGLKDEDDVSIVDIRNEEQLAEYINKNRK
>PvanLCN_ROT76342 .1 putative apolipoprotein D-like [Penaeus vannamei]
MAPSGGALCCVLLLVFLLQTRPVGSYIFKTGECPSVNPVSNFAFAQFAGTWHVIQSSNTASRCYSLSFSENGGSFFLTMN
KQIFSLRAAGIVHNLRFESQIYPNPNNPASMVLRIPSNLYQDVSYQIISTDYTTWATAWSCKPTVFGHMESVLIMGRSRE
LPLSELRSIRLDLETAGVSVDELSSVQQTECNPDLGGGRFTLDLASDLADTLVDWTALIPSSTIPGLSPSGCFVQEGDFY
LYLETCNATASNVIFDPNDPSEGNETGGEDDSADHGVPESSKVPCSAQHQREKIPGEIKPTGPQWTLPGQA
>PvanLCN_XP_027216267 .1 apolipoprotein D-like [Penaeus vannamei]
MKALRLVPALLLLWAGGAKAASCPNLQLMKNFSVDKFLGVWYMVQAEDNIFQTLKSCLASTFTLLAENKIEVNSKGLDSS
GAPATTQSTMEVDPENPAHMVTDFVPRSIPPVKPPFDILDTDYANYACVHSCISVVGFKTEFIFVYARNRTLETKLVEHC
RKLFESNGLSTTNLVDVPQSNCERSEL
>PvanLCN_XP_027222912 .1 apolipoprotein D-like [Penaeus vannamei]
MRFLIATCLLAAVCHGQYVKPGPCPEFTTKLDFEIPPYMGKWYEYARFTTPDQTGQTCNYAEYADNGNGTVAVHNAGLEA
DGTYSEIYGWVEPSDVGGALLLHLNGVPVVGDYNVLDTDYEGYASVYSCQALLGLGHVEQAWILARTPALEQNKIDAAAA
AFTNFGIDVNQFMATPQENCVFP
>PvanLCN_XP_027224697 .1 apolipoprotein D-like [Penaeus vannamei]
MMTRLNLARAAVVMSAVFSVTSSQELFFGSCPQQAVVQNFDLNSYLGKWYEIEKYFAIFELGGKCITAEYSLLPSGNIKV

VNSQANAVTGKATSIEGQAVLADPASGEAKLVVSFSPFGGAPSGGDGNYWVLDTDYTSYAIVWSCSFDIKLMNAQILWIL
MRDQNPDPQRLNYVKTLIRQRGLDLTRLRPTDQTNCP
>PvanLCN_XP_027239352 .1 apolipoprotein D-like [Penaeus vannamei]
MNTLLLVTLAAVTAPSLGHEILWGSCPSVKPVADFDMERFSGLWYVIEKFDNDERCVTWNISKGSDSNTWVLLERKATGF
LSTVGLDQDDLHTATITLDPGRPGVMSISWRLNIVGSYRMTVHSTDYDHFAGAFECQQVAFFQRQQATVLSRKPNLQIDL
RQMARLDRKDIRVEYYRPVSHGGCGYEDTAISAGAGSGDPGPLGGGPSPANFGDFIQ
>PvanLCN_ROT67942 .1 Apolipoprotein D [Penaeus vannamei]
MMTRLNLARAAVVMSAVFSVTSSQELFFGSCPQQAVVQNFDLNSYLGKWYEIEKYFAIFELGGKCITAE
YSLLPSGNIKVVNSQANAVTGKATSIEGQAVLADPASVGGAPSGGDGNYWVLDTDYTSYAIVWSCSFDIKLMNAQILWIL
MRDQNPDPQRLNYVKTLIRQRAHEDQGDWPL
>PvanLCN_XP_027218262 .1 uncharacterized protein LOC113810814 [Penaeus vannamei]
MQTAILVVVLSLAASLAASYKVGFGTCPPVNPMKNFQPAEFQGLWYAVEMFTTNSKCVTLTFNRLGKNDFKVTEGREFVA
AQVVGIDHSFKTKGVYNSRAPQQPAKFHARWSNSESRVGSVFLLFSLAFSVSVCLGNIWQQPEAIIVDTDYWQYAVIVEC
QSVMYFMKRTSAVILSRKRDLDLRVLNQVKRDLCDGYGFDCKDFETIDHNRCTSVQREPARKDVARASPLNRPKPLRKFP
MPEVVRTTPSSKIKPRTFRPITQKPTAAPTTPRVYGKLQRWCLDFFC
>PvanLCN_XP_027214758 .1 apolipoprotein D-like [Penaeus vannamei]
MPNLTICQLLLTALLLAVLGCGGGADGQVLFRGRCPKIPPMRGFDPKRFLGRWYEIERFFVSYEGLAGSCWVENYLYDKQKGFFTRLDWKDHLTGRI
FNIENGLTFDEYEPAVIHYVVQRPSIPILQGEYRILATDYYSYALAWQCDELPLGTAHTQILWFLSKIQHPTYETAKKAKYIAHSIGLDTSLLQKQD
RKDCPP
>PvanLCN_XP_027221670 .1 apolipoprotein D-like [Penaeus vannamei]
MASKVVLAAAALCGVLSAASGRVAPRLEIGECAEFISKPEFNLPEYLGRWFEVQKFENPFQLGMECVTADYTPIDSSSIN
VYNNGVGGDGEFIGIVGNASLIDPNFPAEFQLNFAGRPAGRYNVLETDYETFSAVYACTQLDLFKQEFAWVLARTPQLAP
DALQQAVDVFAAFNIDVTRFQLTDQGETCVYTSPPETN
>PvanLCN_XP_027237807 .1 apolipoprotein D-like [Penaeus vannamei]
MAVLLKFLTALSLFVGAARAQGFMPVPPDDGPCPVFPVVKNFDLHAYLGRWYEIERFFNPFQDGSCVTADYALFPNGSVS
VLNTDVQEGASNTIAGVATLSDDPSAGQLFVEFPFTAGFDGGDLGGNKPNYNVVATDYKNYAVVYTCEFFGPELKFEFSW
ILARRPYIPSSFLADLKAWLQLVNINAKRYMPTVQTDCPNRVAS
>PvanLCN_XP_027235597 .1 apolipoprotein D-like [Penaeus vannamei]
MCRGSMLSVAALAALAVRVAAASTVLEVGACPNVTTVPDLNLQELTGKWYHILHFPSEDEPFGACTQSIYNYTDGNLEVF
TEGLDFAGSDIISVGVLGKLKDSPSEALQLDLDGMPPLSVWVAYTDYTSLLCLYSCTSFPGLRVDWAWALTRTRHPQPRL
MLRCRSKLRNLGVNAAKFERIPQNDVVCKGNRS
>PvanLCN_XP_027233281 .1 crustacyanin-A2 subunit-like [Penaeus vannamei]
MLKALLTAALVALVAADSIPDFVAPGTCAKVANQDNFDLHRYAGRWYQVQIIDIPYQPYTRCIHSDYDYDSVNGFRVTTA
GFSPNNEYIRLQGKIYPTKDFPPAHMLVDFPSAFAAPYEVIETDYDSYSCVYSCIGTDNYKSEFGFVFSRTPQNSASAIS
RCASVFRRNGVDFSSFSAVPHTSDCVYRA
>PvanLCN_XP_027233298 .1 crustacyanin-A2 subunit-like [Penaeus vannamei]
MLKALVAAALVALVAADSIPDFVAPGTCAKVANQNNFDLHRYAGRWYQVQIIDIPYQPYTRCIHSDYDYDSVNGFRVTTA
GFSPNNEYIRLQGKIYPTKDFPPAHMLVDFPSAFAAPYEVIETDYDSYSCVYSCIGTDNYKSEFGFVFSRTPQNSASAIS
RCASVFRRNGVDFSLFSAVPHTSDCVYRA
>PvanLCN_XP_027233283 .1 crustacyanin-A2 subunit-like [Penaeus vannamei]
MGYKEQDAPPGVTHNKHHVEGTLNCCLVALVAADSIPDFVAPGSCARVANQDNFDLHRYAGRWYQVQIIDIPYQPYTRCI
HSDYDYSDSVNGFRVTTAGFSPNNEYIRLQGKIYPTKDFPPAHMLVDFPSAFAAPYEVIETDYDSYSCVYSCIGTDNYKS
EFGFVFSRTPQNSASAISRCASVFRRNGVDFSSFSAVPHTSECVYRA
>PvanLCN_XP_027233278 .1 crustacyanin-A2 subunit-like [Penaeus vannamei]
MLKALLTAALVALVAADSIPDFVAPGSCARVANQDNFDLHRYAGRWYQVQIIDIPYQPYTRCIH
SDYDYSDSVNGFRVTTAGFSPNNEYIRLQGKIYPTKDFPPAHMLVDFPSAFAAPYEVIETDYDSYSCVYSCIGTDNYKSE
FGFVFSRTPQNSASAISRCASVFRRNGVDFSSFSAVPHTSECVYRA
>PvanLCN_XP_027233287 .1 crustacyanin-A2 subunit-like [Penaeus vannamei]
MLKALVAAALVALVAADGIPDFVAPGNCAKVANQDNFDLRRYAGRWYQVQIIDNAYQPYTRCIHSNYDYSDSFNGFRVTT
AGFSPNNEYLRLQGKIYPTKDFPPAHMLIDFPTVFAAPYEVIETDYDSYACVYSCIDTDKYKSEFGFVFSRTPQNSASAI
SRCASVFRRNGVDFSLFNPVPHTSECVYRA
>PvanLCN_XP_027233296 .1 crustacyanin-A2 subunit-like [Penaeus vannamei]
MLKALLTAALVALVTADSIPDFVAPGTCAKVANQNNFDLHRYAGRWYQVQIIDIPYQPYTRCIHSDYDYSDSVNGFRVTT
AGFSPNNEYIRLQGKIYPTKDFPPAHMLVDFPSAFAAPYEVIETDYDSYSCVYSCIGTDNYKSEFGFVFSRTPQNSASAI
SRCASVFRRNGVDFSLFSAVPHTSECVYRA
>PvanLCN_XP_027233297 .1 crustacyanin-A2 subunit-like [Penaeus vannamei]
MLKALVAAALVALVAADGIPDFVVPGNCAKVANQDNFDLRRYAGRWYQVQIMDNAYQPYTRCIHSNYDYSDSDYGFKVTT
AGFSPNNEYLRLQGKIYPTKDFPAAHMLIDFPTVFAAPYEVIETDYDSYACVYSCIDTDKYKSEFGFVFSRTPQNSASAI
SRCASVFRRNGVDFSLFNVVPHTAECVYRA
>PvanLCN_XP_027233291 .1 crustacyanin-A2 subunit-like [Penaeus vannamei]
MLKALVAAALVALVAADSIPDFVAPGTCAKVANQNNFDLHRYAGRWYQVQIIDIPYQPYTRCIH
SDYDYSDSVNGFRVTTAGFSPNNEYIRLQGNIYPTKDFPPAHMLVDFPSAFAAPYEVIETDYDSYSCVYSCIGTDNYKSE
FGFVFSRTPQNSASAISRCASVFRRNGVDFSSFSAVPHTSDCVYRA
>PvanLCN_XP_027233288 .1 crustacyanin-A2 subunit-like [Penaeus vannamei]
MLKALVAAALVALVAADGIPDFVVPGNCAKVANQDNFDLRRYAGRWYQVQIMDNAYQPYTRCIHSNYDYSDSDYGFKVTT
AGFSPNNEYLRLQGKIYPTKDFPAAHMLIDFPTVFAAPYEVIETDYDSYACVYSCIDTDKYKSEFGFVFSRTPQNSGPAI
DRCASVFRRNGVDFSLFNVVPHTAECVYRA
>PvanLCN_ROT62657 .1 crustacyanin subunit A [Penaeus vannamei]
MLKALVAAALVALVAADGIPDFVVPGNCAKVANQDNFDLRRYAGRWYQVQIMDNAYQPYTRCIHSNYDYSDSDYGFKVTT
AGFSPNNEYLRLQGKIYPTKDFPAAHMLIDFPTVFAAPYEVIETDYDSYACVYSCIDTDKYKSEFGFVFSRTPQNSGRRL
AGAPPSSAGTASTSLSST
>PvanLCN_XP_027217734 .1 crustacyanin-A2 subunit-like [Penaeus vannamei]
MLLRAVALAAVALSVPGGLCSPAKEISFGQCPEITLKQDFVLDPYLGLWYEQQRFDNGFEMGLDCANARYTDLGDGTFEL
HNSGRKAEDLFAESFAIGYVHAPGHIIAKFEGHDPVNYFVLDTDYDTFAAVYNCVQLGELRYEYAWMFTRETSASEDLIA
KIHQVFVDNGVDVSLFQVTNQGPDCVYIP
>PvanLCN_XP_027215211 .1 apolipoprotein D-like [Penaeus vannamei]
MAYLTSFSWKVLAILASSLLGEGVLASAASDDAFLIRGECPIIQLESNFDTQRFSGVWYRIGGLPNTEEKSVNCTVYNYR
FTGNGYTVTSSGVGADGAPVTQTTPSSTQRGASPTSPRPSEVSQGAQWAGGGGDGCRADFTADLVVVSTDFTSYACLFSC
YNFQRTHRAHFAWILSRSNSLTPEKIATCQKSLRKVGIPLGKLTKTNQDNCDYSAITG

>PvanLCN_XP_027233277 .1 crustacyanin-A2 subunit-like [Penaeus vannamei]
MLKALVTAALVALVAADGIPDFVAPGSCAKVANQDNFDLRRYAGRWYQIQIIDNPYQPFTRCIH
SNYDYSDSDYSFKVTTAGFSPNNEYLRLQGKIYPTKDFPAAHMLIDFPSAFAAPYEVIETDYDSYSCVYSCIGTDKYKSE
FGFVFSRTPQNSGPAIDRCASVFRRNGVDFSSFNVVPHTAECVYRA
>PvanLCN_XP_027235669 .1 crustacyanin-C1 subunit-like isoform X2 [Penaeus vannamei]
MRGASLPCAVVLAGVIAAAEGLFGIPEFLELGGCANVTLMEDFDPVKYQGLWFDIASVPNEYQVTKQCISQNYTWTGDEM
RVVSRGLAEDGLKVRQGMYMVPEEFEEPFEPAHMTVNYGRMPESPYDVISTDYTTYSCVYSCLEYFGFRAEFFWLFSRTP
WISSWARETCQQDLEDMGIDTGKMLEVLQGETCPYNTRLDAILDENRKLMRKLMSAAPSNVTTSFRRNERVRPADEVITP
RKTARVDTPQKDGDPEAEPEVTDAAATPTATRLVCLLALNVALLPVVAR
>PvanLCN_XP_027217735 .1 apolipoprotein D-like [Penaeus vannamei]
MSRGTWMAPLALLAALFSGSLAANLIGRQVSLGICPNITNKADFDAVPYLGRWFEYERYDILFQSGMDCVNALYSDMGNG
YVEVHNTARTSYGDFTDIVGQAHVIEPGVLLVEFGGYVPAEYHILDTDYENYSSVYNCFEEGDYHWQYAWLLTPPRSPLR
RSSPPPARPSPTTGLTSASST
>PvanLCN_ROT72886 .1 hypothetical protein C7M84_008705 [Penaeus vannamei]
MSRGTWMAPLALLAALFSGSLAANLIGRQVSLGICPNITNKADFDAVPYLGRWFEYERYDILFQSGMDCVNALYSDMGNG
YVEVHNTARTSYDVPAEYHILDTDYENYSSVYNCFEEGDYHWQYAWLLTPPRSPLRRSSPPPARPSPTTGLTSASST
>PvanLCN_ROT67395 .1 crustacyanin subunit C [Penaeus vannamei]
MEAWADRRERAKGGEDTLGVCVRYSGLWFDIESVPNQYQHTKKCVTQNYTWTGEYMNVATRGLTDEDEKVRQGAVMHVEE
YDVEKPDPAYMTVDAAGVPAAPYQIIATDYRTYSCVYSCLEYFGFRAEFAWVFGRTTTLPPSTIAKCHKKFTSMGVDPKK
MFPIVQGAACPYYEKLDEMMATSERQLVALLGPDLPTTSTTTSTTTTSPVTSKLAREEDLLEDIQGTVRLEEKIVKEIET
KVEANQEVNRVAVEEKEGDVDYSSGLRSSASCLLLSLLCITVLMLH
>PvanLCN_XP_027233275 .1 crustacyanin-C1 subunit-like [Penaeus vannamei]
MNAALILFALFAAVAADKIPDFVVPGKCPVVDEKALFEQQKPNHPSFAGVWYEIALTENPYQLLEKCVRNEYAFDGTKFK
ARSTGIAGDGTMMNRNGQILPMPLGEPHLSVDYEESWIAPYVILDTDYENFACIYSCTEYNFGYYSDFSFIFSRSPSLSD
KYIRRCEAAFSNIGVDMSRFTKTVQGSSCSYDSLGSF

>PcarLCN_QEA69217 .1 lipocalin-like protein 1 [Palaemon carinicauda]
MRTSGAALVFVGLLVGIVGAHDWGWGSCPVIPAKAKLNVDKFIGLWYVIELFDSSSTCQTLNYTRMSETQLSVTKSRQFY
ILDSLSIDHANTYKGKLDIPNEDNAGKMRVKWPLNIAGKADYTIFDTDYDTYAVIYECQEVSTLAYRQSAAILSRSPTLD
SAIVDRVKNQLKGAGISISHFDKIDHSVCTQPSDSSMKIDIDDNTFKDIVDSAGSGIKSVASTVANGATNLADTVVDFTR
RFGASTDGSSASATLQEEKYADVEIIS
>PcarLCN_QEA69219 .1 lipocalin-like protein 3 [Palaemon carinicauda]
MWLYYSILSAFFFYHVSAHTIHFGRTCPNVQPFPNLAVDKILGTWFVLHKTDTDNNCLVWNLTRGAMPDSLKVTETRQLS
VLDVFRVDHTHAITARIDIPNPEVPGKMRIRWPTLALTGKADFIVFDTDYETYMAIFECDRAGFLHRRSAAILSRTQDID
ENMVKRVRRLLETADVGHYYLSEISHDACRVEGKTKWHIDREFFGLAPGEDAAAEVMNSNKEVTNDYDITQLEIVEEEAN
PSYSFRGSLREGRPDI
>PcarLCN_QEA69218 .1 lipocalin-like protein 2 [Palaemon carinicauda]
MSVLRVAVTSLVIGLALSAETFWGRCPSAPEMADFDMAKFEGLWYLHETFDPGEKCMTWTITKGTEPNTWKVVEEKESGA
INAVGLAHSDFNSGTITMKNPSKPSQLRIYWPISGIAGDAALTIYDTDYVTYAGAFECQNVGFFHRQNGIVLSRNPQMEV
ADRQNSRIKTPRIKVEYYQRVLQGNCRYKYNEDNATTTGEEWQGYHGEDGIYAVGEEY
>PcarLCN_QEA69215 .1 crustacyanin-A1 [Palaemon carinicauda]
MKMVGFFLLVLGIVGSVISEETPDIPEFLQRGNCAKVSVLPKFDLRRYSGRWYQTDVIEIPYQPYTKCINSFYEYSEPKY
GFNVRTAGLTPEGEHLKKEGKIYPTERFPASHMLIDFPTVMGYPYEVIDTDYDTYSCVYSCVDWNAYKTEFAFVFSRTPE
NNGPATAKCKSVFSKRGIDFSKFVAVPHTDECVYRA
>PcarLCN_QEA69216 .1 crustacyanin-A2 [Palaemon carinicauda]
MLRFVFLVLGFVVSVISEETPDIPEFLEKGNCAKVGIFPKFDLRRYSGRWYQTEIIEIPYQPYTRCINSFYDYSEPMYGF
KLRTAGFSPEGEHMALEGKVYPTKNFPPSHMLIDFPTVMGYPYEVIDTDYDTYSCEYSCVDWNAYKTEFAFVFSRTPENN
GPATAKCKSVFTKRGIDFSKFVAVPQTDDCVYRA
>PcarLCN_QEA69214 .1 crustacyanin-C2 [Palaemon carinicauda]
MFSKVLLLAVAAAVLADDIPDFVIKGNCPAVEEQRLWDEQVPKHYKFGGVWYQQAISTNPYQLLKKCVRIQYDYNGKGFD
VKAVGITPEGSQLKRTGKIAPMPLGDSHLMINLDNSFPAPLVILDTDYDNYACMYSCMDYNYGHHSDFPFFFTRTPDAPD
KYINKCKEALDNIGVDPSRIIKTEQGNHCNYDQLKSLIRD
>PcarLCN_QDH76668 .1 crustacyanin-like lipocalin [Palaemon carinicauda]
MFSKVLLLVVTVVVVADEVPDFVTQGKCPAVDEQKLWEQQLPRHADFGGVWYQQAISTNPYQLLKKCVRIQYDYNGKYFN
VRGVGLTSEGTELKRIGKLSPMPLGDPHLMINLENSFPAPLVILDTDYNNYACMYSCMDYNYGYHSDFAFFFARSPDAYD
KYINKCKAALDNIGVDSSRLIRTEQGRDCNYDQLKKLLRDEL

>PtriLCN_MPC19037 .1 Apolipoprotein D [Portunus trituberculatus]
MKVVGVAVMMVMVVSTAAGHDWGWGSCPSIKTFPNLDVDKFLGMWYVIQQTDTTSSCLTMDYSRSSPTQLRATKSRQLLV
LDSFSIEHTNSYTAKLDMPDLDNPAAMRVKWPLNLAGKADYKVVDTDYDNYAVVFECQKFANFLHRKSTAILARSPSLSN
EIISEERKALNDMGISTGDLDAIDHSVCVRREDSGLNIDIDEETIKKMLKDASQSVRNIASQLAGGASDLAASLAELSRT
LASTGSNQEAVAIID
>PtriLCN_MPC58924 .1 Apolipoprotein D [Portunus trituberculatus]
MQASGRSVAMAVVVVSVLAATTTAHMMGLGSCKNFEGMQNFDPARFNGTWYVMNKVGTRSDCLVVTYNLMEDDQSFEVKE
VRRPPYSDVIPLDFAITNTGSLTPKGDSGKFTVRWNNGIIPFTGTYAIPNTDYENFAIDIECQEFFGFLRRVSATILSRN
PEMASAKVEELLDELASQFEIPRNRMNAVDQSNCVEMEANDFNFVVDENGIRSALKELKKIEYLSKLSPEQAAEIVETFE
N
>PtriLCN_MPC18808 .1 hypothetical protein [Portunus trituberculatus]
MVTTNAIFRSMQVLGSWYVLHKFDTDNTCLMWNMTHGPLPDTLLVKEIRQLAVLDTLGVDHTHAITARVDIPNPEVPGKM
RIRWPTCKCWAPVRRLLDAAGVSHSALESIDHDLCKTKGKNNWHVDEELFGLLPGVTEEEVRRRVSEDVENYDISQIEII
GDGVIPEGDFRGSIIRGIPGNSGIKSKPHV
>PtriLCN_MPC40350 .1 Apolipoprotein D [Portunus trituberculatus]
MLRVLLLCVAGFGLCFGQVLKPGKCPEFIPKTDFEVEPYLGKWLENARFPTSYQINQTCNYAEYSDRGDGTVGVHNAGLE
ADGTFSEIFGYVELTDVPGSLALHLDGVPFVGAYDVLETDYETYSAVYSCLNLIGLRHMDQAWVLGRHMLSEEELNIALS
AFTRWGIDISSFEYTPQTDCSALP
>PtriLCN_MPC38434 .1 Apolipoprotein D [Portunus trituberculatus]
MKATITSLLLAVVVALSEAKVGALRHVSTGKCPTITNKQDLEVAPYLGRWYEIERFEARFEEGMDCVHVDYTDLDIEGRN
PAKEIVHSADEVKRFSDSNREKEMDVMISAFCLLSTDLRA
>PtriLCN_MPD04706 .1 Apolipoprotein D [Portunus trituberculatus]

MKAAVAALLLAATVVLSEARTTKLGVLRDVGECPTVTTKQDFDMLSYLGSWFEIERFDALFEEGMDCVQIVYSDLGEWEG
KCQDRRIQPSQRE
>EsinLCN_AYU71035 .1 crustacyanin-C2 [Eriocheir sinensis]
MKVTLVSILLAAVVAVGQTMAAKGLIARDVSVGICPTFSNKEDLDPVPYLGPWYEIERFELAPEKDMDCVKAVYSDLGDG
LVEVHNMARSNGFFTEIYGTATVIEPGVLLVEFPGSVPSEYHVLDTDYTTFSAVYNCEQVEEMRFQYAWILSRTPTMDQE
TYDYARQVFVDNGIDVTIFQPTYQGDDCPYVE
>EsinLCN_AYU71034 .1 crustacyanin-C1 [Eriocheir sinensis]
MKFLIVSVLLAVVAGLSNAGTVQRLGMFRDSSLGSCPNITNKEDLEVVPYLGAWYEIERFDIIWQLGMDCVEAIYSDIGG
GWVEVHNVARKPSGEFTEIIGNATVIAPGVLYVIFAGHVPAEYHVLDTDYTTFSSVYNCFETGPERFQYAWILSRTTTLD
DATYDRARQAFADNNIDLSLFHPTYQGDDCPYLGTQ
>EsinLCN_AYU71036 .1 crustacyanin-A1 [Eriocheir sinensis]
MKAMLVTLLAVAAVAQSQAKEGEKFPSGIVSVGECPSITPKEDFDPAAYLGAWYEIERFNMIFEEGMDCTQSVYSDLGDG
VMEVHNVGRTSQGPFTAVGTVTVEEPGALLVAYEGYIPETFYVLDTDYTTFSAVYKCEQMGELRSQYAWIVSRALTLNQA
TLSHVHQVFASNGVDVRLFQPTHQGRECPYLNNKQEL
>EsinLCN_AYU71112 .1 crustacyanin [Eriocheir sinensis]
MKMLRVSAAVVVLVCWTGSGGGISLVDASTLLEVGACPNVSSVPNLDLAKLEGQWYQILQIPTDDNSIARCTRSTYTYAD
GYLEVVTEGRDAVGGAVARTGVLARLPDSPTGALQLDMDGMPPLSVWVLDTDYSGVACLYSCTEFPGLRAEWAWAVTRSP
RPRVKLVHRCRAKLRRHHVDTAELERVPHSAQCLKGN

>EaffLCN_XP_023325324 .1 uncharacterized protein LOC111699024 isoform X1 [Eurytemora affinis]
MGKVHLFSFLLLPAVFSHEIYPGKCPDFTPMKQFDWTKFSSGVWYVIQKFDTKSSCLTYEFKTDNLGFKSVEQIRQLPFK
EAIGVDHQYKYTGKLFAPQESEPAKMVARFPLNAIGSASYVVTDTDYDNYGMLCTCQVSVIHFHFILYMSG
>EaffLCN_XP_023332463 .1 apolipoprotein D-like isoform X1 [Eurytemora affinis]
MVTLFGLSMGTTKSFGSCPPVRGVENFEVQRYLGVWYEYSSVFELFSIGARCVRATY
SDLGNNTLRVFNEQVNYYTGNYGNLEGYAKPANETSDIAEFIVIFPDIPGGGRKTAPNYNVVKTDYESYAVVYNCNQYPL
IKTESLWLLTREQNPSQELVEQLYSEMREMGLPVDSFYKTEQSNCTQLPEPGTVQYKFSPESLG
>EaffLCN_XP_023342265 .1 apolipoprotein D-like [Eurytemora affinis]
MLWLLCLACFVSFGQTAVWNLGACPDIPGMDNFDLTKYMGTWFEYSNVFEIFQIGAKCVRAQYAIKGETISVKNEQVNMW
WNSYQQVQGSARPADPAKPNKAELVVTFEGIPFQSTSSGKVAPNYSVVDTDYTTYAIVYQCTTFPVWRRESLWVLTRRQV
PEQALVDSVYKKMTSLGLPVQNLEKTEQEGCSLLP
>EaffLCN_XP_023338682 .1 apolipoprotein D-like [Eurytemora affinis]
MLTLVCFISCLAAVQAAVPSFGACPNVRGMTNFNKNSYLGVWYEYSNVFEIFQIGSSCVRATYTDLGTNVGVLNEQVNDL
TGYGNITGTARPAALSGEFIVGFSSVPFASGGTEPNYRVVDTDYTSYAVVYSCSPFFFFMKKESLWLLTRQQTPQQYVVD
VGYSKMRALGLPVAALEKTPQTNCIRLPLPSTTTATAGK
>EaffLCN_XP_023334392 .1 apolipoprotein D-like [Eurytemora affinis]
MLIIVALSGLFCVASAGPMQSFLGGIRAQCPPKPPTVSTLDAAAYLGKWYEQKRIPASFQLNTRCVRAEYGLNDEGVITV
HNTATKQDGSFDEIFGTATVPDPNHPGELKVHFPESPVDGDYWILDTDYENYSIVYSCVDYIFGLIHFEFAWILARDTNM
DPALIQKGVDLLESYGIDISLLEDTVQTEDCFYG
>EaffLCN_XP_023324477 .1 uncharacterized protein LOC111698387 [Eurytemora affinis]
MRIFVVFSLLQGLMANRFVNRDMVGASHDVICPISQAKPNFNIDLFMGHWYILEYQYPREMKLTDLSCLSFDF
NAAGEDIVGNFSFRYPPRFGHYYHIPTVSNVIANGQEGLWMTSFKGVSLLSGIVDTDYENWAVFVQCVNESGENKFMSTR
VLSRTRDLSPQDWLTVREAIQV

>TlonLCN_KAF2366362 .1 Lipocalin/cytosolic fatty-acid binding domain [Trinorchestia longiramus]
MKGLLLLLFMTTIASTQGHDWALGACPSVKPLDNLAVDKFLGFWYVIEQFDTSSLCLTLNYTRVSESELKVTKLRQFSAL
DRVGIEHTNSYSGDLDMPHENDLARMRVKWPLNIAGKGDYIVFDTDYTNYAGIYECQTVTALMHRKSAAILSREPTLAPE
FVDRVKRRLTSFGISTRHFDRVSHQGCRSRADADVNLHVDGDTFTSVVSGARDGIEDAASTFMKGIRDLATTVSSATQNT
FNSFQGGDSDTASKNEGELQKPVEETINEGLGAAGETINKGVETVEETLKKGVEAAEEALGDAVDKVEDALTGVNEFTIE
RNGDKNAPADGTPLPDENINSVHTDDSLQENFYAVQNAPELPAGDVIDTVVRVE
>TlonLCN_KAF2356095 .1 Lipocalin/cytosolic fatty-acid binding domain [Trinorchestia longiramus]
MDFKNTLLLVLVAACVGLVSPHTVHMGRTCPEVDPFPNLSLDKILGVWYVVYQFDTSNTCLVWNITRVGEERLAITETRQ
LWLLDSLRIDHQHLLTATLDIPNPEVPARMRIRWPTSITGKADFTIFDTDYQEYMAVFECDRAGLIHRRSVTILSRASVM
NQMFVDRIRRILDTKNIPHAALNIIDHNICREQGQYSWHTEGELFGLLSRDASQTQEQRLASGVEGYDISQLEILGDGVL
KNADGTEFKGSIKVPRST
>TlonLCN_KAF2348293 .1 Lipocalin/cytosolic fatty-acid binding domain, partial [Trinorchestia longiramus]
MVSVVALVLLALGISDAHKLGLGPCQDVTPMQGFDVSKFYGRWYINEVFEAVNTCMTLDFEEKTDNSNEIKASQGRQISPL
DAINVKHTTNYAGTIIKGDSETPARMRVKWPVSGIFGSLTYTVVDTDYTNYALTFECASFIGVRYYSASILSRNPQLDPTV
MEEIKAKATENGIDVSNFQVVSQQECTKLGEGIDINTDPDDYNLLGLISDDKMQGIKSDDDLIKIFEGNATPKQ
>TlonLCN_KAF2369086 .1 Calycin [Trinorchestia longiramus]
MEPVKQTLIGLVVAILVPNLYASKSTFGSCPKVTPQENFNLTRFEGVWYAVEVFDDNVKCMEWSITFEGDNIWHIKETKD
SGVIGEYNGKLTPNDEQTGALTVKWSANIGSYPLTVFTTDYENYAGVFLCQQVQKMTWLITSNRCDSTAGRKEWFSHAPP
SCISRFDNRLEEWLKNLASTCRTSRKWTKETASVI
>TlonLCN_KAF2367273 .1 Lipocalin/cytosolic fatty-acid binding domain [Trinorchestia longiramus]
MKVLVVASILFVLGGSEGIALYSSPTSLRLTKRQAIDDTFGTAPKTIIYRNPRSPTDQPLQCPKPPIVQNFQPEKYLGRW
FEIERFETPFQTGHCVTADYTLLSNCSIGVSNTQVLSNGTVDVAKGVAHSAGDPSQGSLTVSFPEEERQRGKVQSPGNEG
NYNILATDYVNYAVVYSCSIADIRGKMMKFEFSWILARLPRVNTVFLQRLHQWLQNVGVPVERYARTQQARCKYTTA
>TlonLCN_KAF2351042 .1 Lipocalin/cytosolic fatty-acid binding domain, partial [Trinorchestia longiramus]
FSVALLLALAGLSNAYLDEGECPPFQTTAEFEIPLYLGRWFEAERYPTAFETDQSCVTADYGAIDDTTISVTNTAILPDG
TVTSISGTATATDVPGQLILQFPNEAVGFYNVLGTDYENYSTVYACSQVGTYRTQYAWILSRREVLEEEYLTMAMDVFTA
NGIDTSVFMPTPQGGDCMYVDPPEPV
>TlonLCN_KAF2361791 .1 Lipocalin/cytosolic fatty-acid binding domain [Trinorchestia longiramus]
MWAVTTFLCVFVALAGASSSPSFLKRGHCKKGVEPVRDFDPNQFVGRWFRIGGVRNPDEDRTITCTTLDFRVKGTGMDMT
AQGLDHADEFTMRVTPLSLDDPNVASFGLTSNNRPVSLEVLETDYTSYACLFSCTDIAKTHFAQFAWILSRQPTLNKTQI
GKCQLALKEAGVAVGKLKGTRQGGQCNYPPPLP
>TlonLCN_KAF2348121 .1 Lipocalin/cytosolic fatty-acid binding domain, partial [Trinorchestia longiramus]
FSVALLLALAGLSNAYLDEGECPPFQNIAEFEIPPYLGRWFEAERYPAGFEADQTCVTADYGPIDDTTISVTNTANLPDG

TVTSISGTGTATSTPGQLIVQFPNEAVGFYNVLGTDYENYSTVYACAQVGNYREQFAWILSRREVLEEEYLAMAMDVFTA
NGIDTSVFVPTHQGEGCIYADPPE
>TlonLCN_KAF2356926 .1 Lipocalin/cytosolic fatty-acid binding domain [Trinorchestia longiramus]
MDLRSTTIITALLCSLAKTSAEIGLFGCPSQAVIGNVDITQYAGTWYVYSMYERRKQSEYDCPQEKYTPTSNGLVKVEHQ
YKRNNVFISKIGQMKFADPSSTEGKLSLYFTEGAYEDQVNDATKVNYQILATDYTGYSIVWDCTSLFFLHFTRLRILTRE
RSPPQAMIDNVQSQLRDRNIKVSLLRKNNHDDC
>TlonLCN_KAF2366412 .1 Lipocalin/cytosolic fatty-acid binding domain [Trinorchestia longiramus]
MPYFDRYRYEGLWYENQRYFTLFEAGGRCMTILHEDAGDGRFAVTNSMVDWFGKRVTVSGFASIIDTKGPVGAKYNLRYY
SNPFLALASKGKTDGNYWVLDTDYDNYAIVWSCNDKLFSNTQFLWILTRLRHPDDWVVDRAIWEVERRGLDISELKTTDH
TGCPPAFDSYELGYDSWNSIYAPSLSPYTVPPVTGVGYSQPLGNAVVSRPYYDFGVRSKLNEYNSVSQERTAWNQSSVPH
TEDSSYLQYTPLPAMNMFQNFTQANMLPKSSYFDTVVAHNSSNALNISPLPATKFSDNRYKKVKVAQERIQVKAGTSSPL
QNGWQVTDYHQVLQLRSSQ
>TlonLCN_KAF2349367 .1 Lipocalin/cytosolic fatty-acid binding domain [Trinorchestia longiramus]
MTMVWSLQEVLTAKKRHQTLLVLPVLLSLVQAQVLFGGECPPLKPMAYFEPNQFLGRWFEIARFYVSFEGLAGTCWVENY
HYDRNRGHFTVLEWKDHITRRIRTVENGILHDKKEQGLLKYSLQRPNLPFIRGKYLILATDYERYALAWQCDNLPLGVAH
TEILWFLSRDQFPDPSVTKKVIELTKKFGLNPSHFEFQNRKGCPH
>TlonLCN_KAF2356925 .1 Lipocalin/cytosolic fatty-acid binding domain [Trinorchestia longiramus]
MRGLSVGFLILAFTLASVEAGFGLGRCTYQPVVDSLDFSQYAGDWYEWGRYFIVSQVLAECTKNILTENTPFMDVEVEYI
SFDRIRSKKGQLKYANPDKSDGVLTFDYSVDAFKGKAEMHTFPNYNVLDTDYDSYAIVYNCADLFFAHYDDVYIYTRDRI
PDPSLIDDIFSELYKRNIDTSIIARTNQVDCTN
>TlonLCN_KAF2367221 .1 Lipocalin/cytosolic fatty-acid binding domain [Trinorchestia longiramus]
MLCLKVLIFAITWMHPATAGICPSYPIIRDFDFSKYTGLWYHQDKQQTSWGSPGRCWQSIYVRDGKSGEYRMKLRYQNTI
TGRSGLLESTLTLQGPRKPSVIKYSIPYTFMTDEFQILATDYDSYSIEYQCHTNSILPKTENVFLLTRDKTPRAEVLQKA
YSIMHRYGIDLRKLERVDQTCGGLSLEGVLRPEEMIAVQPWGDPVRETLPLLEQNKGVVYMGGNQVYDAGTVEAVRRRDQ
HVSASHISSPLRRRRQQPMRWSDDAQRRVSLGQSLATRLWHVLHTPFK
>TlonLCN_KAF2354569 .1 Lipocalin/cytosolic fatty-acid binding domain [Trinorchestia longiramus]
MLTALLAASSLVCMAQLVYPGACLPITPVQNFDLKKYAGLWYENRKYFAFFQLAGTCVTATYVGIPGNDSAVEVLNDGYFF
GSPKTVIGVAEKVDPSSTEAKLRVSFTSTPFAAAPSERGNYWVLETDYTSYAVVWSCRQDNDDSNRQNLWILTRDRVPTVR
TLAKARKAIETRGLDYNSLTVTDQYNCS
>TlonLCN_KAF2356922 .1 Lipocalin/cytosolic fatty-acid binding domain [Trinorchestia longiramus]
MNKTFLFSILTLGLMAQAQEIFFKKCPSPTVVKNLDLNRYLGKWYENRRYFAIFQIGGRCTTAIYTDAGNGLVKVENIQT
NLFGKRNNVVGQAKLANPESTEGKLSVSFSSETPYENMANEVTDSNYWVLDTDYTSYAVVWSCRNALIFNTQFLWVLTRE
RNPPTTLINRALAVIKERGLDATRLSITQQTGCKN
>TlonLCN_KAF2356923 .1 Lipocalin/cytosolic fatty-acid binding domain [Trinorchestia longiramus]
MNKAFLFIILTLGLMAQAQEIFLKKCPSQTVVENFDLSRYLGKWYENRSYFAIFQIGGRCTTAIYTDAGNGLVKVENTQI
NFLGKTSGVVGQAKLANPESTEGKLSVSFSSETPYENMANEVTDSNYWVLDTDYTSYAVVWSCRNALIFNTQFLWILTRE
RNPPADVIDKALAVIARRGLDATRLSITEQTDCKN
>TlonLCN_KAF2356924 .1 Lipocalin/cytosolic fatty-acid binding domain [Trinorchestia longiramus]
MRGLSVGFLILAFTLAFVEAGFEFGRCSFQPPVDPFDFNEYSGEWYEWGRYLTPSQIVAGCTKNILTPNGPLLDVEVEYI
FLDRIRAKKGQLKYSDPDKTEAVLTFEFSANGFGDRQNEYTFPNYKVLDTDYTSYAIVYNCIDLYLFQFTDLYIYTRDRQ
PTEDVIANVYMELYKRNIETFGIARTDQGNCPN
>TlonLCN_KAF2362181 .1 Lipocalin/cytosolic fatty-acid binding domain [Trinorchestia longiramus]
MEGLDLNKFAGTWFTIERIPNQYVETASCIYTNYTVDGSGLLTHEFGKNAVGEAITIQSQMKANKPGEAMTVISDGVPEA
PLKIVATDYDTYACLESCLQYPAFKAQFAWILTKKPASSIEAAKTCHDILKTKMSIDSSSFKKVKQGRGCEYPTWARNSN
>TlonLCN_KAF2361275 .1 Calycin [Trinorchestia longiramus]
MLGGRLLTLPPVSGFVPDFIEWGYCSEVHLQENFDPVKYEGWWWDIFRVPNSYEGVDKCIHQNITLKDDVMVVKTEGLSA
KGIFSNKTAFLSTDTSHPVRNPARRTVDAEGVPAAPYLVMATDYLQYSCVYSCLQFPGVFAEFFWIFGRQPTLDEKYTQE
CIHHFRQAGVQPGKLEKVTQGEACPYAPRLKAMQTTNHALLKDVKRDERSEKLLSHSSSSTTDSGLLREGTSAAGRAVFW
LECWGVSVMTATALWALQQCRCLCS
>TlonLCN_KAF2368084 .1 Lipocalin/cytosolic fatty-acid binding domain, partial [Trinorchestia longiramus]
VVRVSVVVLVCFSYIRPVITLGPSFVRFGFCSDVVQQPDFDMEQFTGLWYGILRVPNEYSPLKRCITSHYSAADENGKFT
NSRNGVAENGTEVHSELELMVVDDEFYAYSIPPRKSRPEIGVTVAETDYTNFACVYSCIQRFTFKAEFVAVMSRTPSIKK
AYVEQCRATITEQSSFDWSHLSMVEQGE
>TlonLCN_KAF2360803 .1 Calycin [Trinorchestia longiramus]
MLASGKTRVRIPLPTWLTQLEIRHGTWVKQPNNYRSNNPTQEWARSFAPSNNSFFNRLRTPVKIFVNIFRKQPYAAPNRL
SYTISGVPRTIFEDHYEVAGTDYKSWTIEYSCRNLPYGSIRFAWILTRSPHPPSKVIKKAKAVMAALGIDPHLLQRHKNT
CVKAY

>HaztLCN_XP_018021503 .1 PREDICTED: uncharacterized protein LOC108677745 [Hyalella azteca]
MLGYSLLLVSLLGITRVAGHDWALGACPSVKPVDNLAVDKFLGLWYVIEQFDTSSLCLTMVRWPLSQYIRVRWPLNLAGK
GDYIVFDTDYTHYAGIYECQAVTSLMHRKSASILSREPTLSPDYVDRVKRRLTSFGIDLRHLDRISHEGCRSKADADVNL
HVDGDTLTNVMSGARDGIEDAANTFINGIRGIATTVSNVTQNTFNNFPGITTSTFGNTVTNTPSTGFGGSSTTTTSPATT
KSVDVNIRTGSTVPDEDVNSVLTEDNLEENFYSVANSSITPSEDEIYRVVRTE
>HaztLCN_XP_018017076 .1 PREDICTED: apolipoprotein D-like [Hyalella azteca]
MKQAMTAVNPVMAVVLLLTLGSAAGHKLGIGPCPDVPSMKSFDVNQFFGKWFINEVYANINTCMTLTFAAIPNSTTEIKA
TQGRQLPALDAVNVKHTTTYVGTIITGPQEAPAKMTIKWPLSGAILGRMAYTVIDTDYKNYALTYECRSFLFGRYYSATI
LTRETLLDPTVMEEVKKKAAEAGLDTSLFKVVSQKTCTAPGEGIDFSQHEDAYNLLGLISDDKLQAIKTDAQLIEILGHK
PPAQP
>HaztLCN_XP_018009192 .1 PREDICTED: apolipoprotein D-like [Hyalella azteca]
MKLLSSVLTVAAMAVLAPGAQASVSVRGSCPAVAAVSDFNMTRFEGLWYVIEVFDKGVACMEWKIAPMEADVWHIKETKQ
SGYVGEYNGKLTRNDNSSGSLTVNWDANIGRGFPFTVYATDYDTYAGAFMCQQVWFFNRQEGVILSRSPVLHIAQRQQTR
GMLSRYGVEMSYFSKVEQGLCGRTSLPDAGAGSDNKRFDEYDNLSSDA
>HaztLCN_XP_018020509 .1 PREDICTED: apolipoprotein D-like isoform X1 [Hyalella azteca]
MIKVIVLAALLGSECVVAQYSAAEAHSFSKRQATDDTLGTAPGTIVPGPADSGPDQTPQCPKPPVVQNFQAERYLGRWYE
IERFTAPFQTGICVTADYALLGNCSIGVTNTQVLPNGVVDVARGVAHSAGDPGSASLTVTFPDEERTRGPVQTPQTEGNY
NVLATDYENYAVVYSCSIANFRGRDTKFEFSWVLARQPRVNSVFLRRLHEWLQNLGVPAERYMLTRQAGCQYTAA
>HaztLCN_XP_018020510 .1 PREDICTED: apolipoprotein D-like isoform X2 [Hyalella azteca]
MIKVIVLAALLGSECVVAQYSAAEAHSFSKRQATDDTLGTAPGTIVPGPADSGPDQTPQCPKPPVVQNFQAERYLGRWYE

IERFTAPFQTGICVTADYALLGNCSIGVTNTQVLPNGVVDVARGVAHSAGDPGSASLTVTFPDEERTRGPVQTPQTEGNY
NVLATDYENYAVVYSCSIANFRGRDTKFEFSWVLARQPRVNSVFLRRLHEWLQNLGVPAERYMLTRQVGCQYTAS
>HaztLCN_XP_018012794 .1 PREDICTED: apolipoprotein D-like [Hyalella azteca]
MNGFSAVLLLAVAGLTNAYLDQGVCPDFQNIAEFEIPPYLGRWFETHRYPNGFETGSKCETADYGAINDTTVSVVNTANL
ADGSLYVISGTGTATDVPGQLIVQFPGESVGFYNVLATDYETYSCVYTCAQIGDLRDQYAWVISRTWELDDATLAVVLDV
FESNGIDTAVLQPTPQGGDCIYTPAP
>HaztLCN_XP_018012730 .1 PREDICTED: crustacyanin-C1 subunit-like [Hyalella azteca]
MQTVAAILWVFVGLAAANDSPSFLKRGSCKTGVQPLSGFDRNKFTGRWFRIGGIRNPDEEKAVKCTTLDYRVGGTGMDMV
SQGLDHIDEFTRKVTPMTMDDLTVGSFGLTSNNRPVKLEILETDYTSYACLYSCTDIAGTHFAQFAWIMSRQPKLDKREI
GLCQLALKEAGVPVGKLKGTRQDSACNYPPEE
>HaztLCN_XP_018013962 .1 PREDICTED: apolipoprotein D-like [Hyalella azteca]
MKWSVLVLLGLLAAAKAEITAGICPEWTNVETFDIPSYLGRWYEMARLPVAFELGISCVTVDYGPLSDTMITVTNNGDLG
NGIVTVEGTGTATNVTGQLMINLNGEDVGLYNVIDTDYVTYACVYTCEQLGDLKDETAWAFSRTTSYDPETRDKVEAAYT
KFGIDVTKFHITPQGGDCVYAPLP
>HaztLCN_XP_018022291 .1 PREDICTED: apolipoprotein D-like isoform X3 [Hyalella azteca]
MLKFVLMIVCLLSIAAQAQELFFKKCPNPSVVSNLDAKKYLGKWYENRKYFAIFQFGGRCASAKYSDAGNGLIKVENNQL
NFFGKPSGVVGQAKFANPSSREGKLSVSFSSPTPYANNSNAVTDSNYWVLDTDYNSYAVVWSCTSALIANIQFMWVLTRD
PQPSEALVNKTLTIIRNKGLDPTRLSITDQNNCK
>HaztLCN_XP_018022292 .1 PREDICTED: apolipoprotein D-like [Hyalella azteca]
MKSLLVLVISLLATAAAQKLFFAKCPSQAVVPNLDVKKYLGLWYENRKYKTIFQLGGRCTTATYTDAGNGIIGVRNDQKI
TVIPVSVVGQAKFADPTKTEAKLTVSFTSATPFANDTSSVTASNYWVLDTDYDNYAIVWSCQAAIIANIQFLWVLTRQRS
PPEAIITKVLGVITDRGLDTSKLDVSKQDLCF
>HaztLCN_XP_018019164 .1 PREDICTED: apolipoprotein D-like [Hyalella azteca]
MNGFSAVLLLAVAGLTNAYLDQGVCPDFQNIAAFVIPSYMGRWFEAHRFQAGFEDGMTCATADYAAIDDTTISVTNSAVL
SDGTLDAISGTGSATGVPGQLIVQFPGEPVGFYNVLATDYETYSSVYSCRQIGDLRDQYAWVMSRTEELDADSLAAALDA
FESNGIDTSLLQATPQGGDCIYAPVPPKI
>HaztLCN_XP_018027662 .1 PREDICTED: crustacyanin-A2 subunit-like [Hyalella azteca]
MTATVALVLVTLQIVSPALGQIIPEFLGFGYCPETPPQADLDLAKFSGTWFTVTRVPNVYDALASCIYTNYTLDADNNLA
VQAFGKNAAGEETVVNSAIKSNKPNEPLTVVSTGVPEAPLKIVATDYSTYACLESCLQYVAFKAQFAWVLTKKPASSIEA
TNICTEIFKNKMGIAASTMKKVTQGRGCEYPSWARGGN
>HaztLCN_XP_018018424 .1 PREDICTED: apolipoprotein D-like [Hyalella azteca]
MNGFTAVLLLAVAGLTNAYLDQGLCPDFQNIAAFEIPPYLGRWYEAHRYPAGFEEGNTCVTADYGAIDDTTISVTNNANL
PDGTLDTIVGTGTATGVPGQLIVQFPGEPVGFYNVLATDYETYSSVYSCSQIGEFRDQYAWVLSRTEEMPADSLTAALDA
FESNGIDTAVLQPTLQGAGCVYAPVPPKI
>HaztLCN_XP_018022290 .1 PREDICTED: apolipoprotein D-like isoform X2 [Hyalella azteca]
MLKCVLMIVCLLSIAAQAQELSFFKKCPNPSVVSNLDANRYLGKWYENRKYFAIFQFGGRCASAKYSDAGNGLIKVENNQ
LNIFGKPSGVVGQAKFANPSSREGKLSVSFSSPTPYANNSNAVTDSNYWVLDTDYNSYAVVWSCTSALIANIQFMWVLTR
DPQPSEALVNKTLTIIRNKGLDPTRLSITDQNNCK
>HaztLCN_XP_018011456 .1 PREDICTED: crustacyanin-A2 subunit-like [Hyalella azteca]
MTGYILPLLSVVAIVPVAAGFLLPSFLEFGQCFAYPPAADFNMTKFTGTWYTVQWVPNEYIPIVACTNANYTVVDDHVVC
NERGLDAQGEKRSRVTKLDLLTDDTSTFSARGEGLRPTAPMNIIATDYDTYACVYSCMKTFAMKAEFAWILSRHPLLDKP
SMEACMEAFTEQHEFDLGKLESVEQGKTCPYWSHLGLPEKTLFLYSSVNDISLTEHVGKKSAQWQGRTPGHRGPVSASTL
PRCSHLSVLISVISLLCMHR
>HaztLCN_XP_018027613 .1 PREDICTED: apolipoprotein D-like [Hyalella azteca]
MVMPAPKTSLALLLALLVAQGASQVLFGGACPPLKPMTYFEPNQFLGRWFEIARFFVSYEGLAGSCWVENYYFAPKRGHF
TVLEWKDHLTGRIRNIENGIVHDNKQQGLIRYELQRPNLPFLRGHYLILATDYRRYALAWQCEDLPLGVAHTEILWFLSK
DQFPDPTVTQKVLELTKKFGLNPSHFEFQNRKGCPH
>HaztLCN_XP_018012146 .1 PREDICTED: apolipoprotein D-like [Hyalella azteca]
MCQFSSHAPRRFSAVLLLAVAGLTNAYLDQGVCPDFQNIAAFEIPPYLGRWFEAHRYPAGFEEGNTCVTADYGAIDDTTI
SVTNTANLPDGTLDTIVGTGTATGVPGQLVVQFPGEPVGFYNVLATDYTTYSSVYSCSQIGEFRDQYAWVLSRTEEMSAD
SLTAALDAFESNGIDTAVLQPTPQGAGCVYAPVPPKI
>HaztLCN_XP_018011458 .1 PREDICTED: crustacyanin-A2 subunit-like [Hyalella azteca]
MYLFITLVLLAVILPCQAFLSPPSFLRFGSCSEVPVQGGLNLQKFAGLWYTVEKMPNEYDPAKFCINTQYSYHGDHMESV
QRGLYEDRSKQKLMTFLVVPNVTDPVMTVIADGMPNAPLKIIATDYDNYACMYTCLGFVGLKAEFGMVLTRQALPPPSAV
QVCRDLFENSTDMNISTMEIIEQGKNCPYWSKLDKQDRDLYESYLTSRSLVSEKTDQDRETAAGALHDTLSNSAARAKLL
DLSLALTASLVFLLRHFS
>HaztLCN_XP_018012796 .1 PREDICTED: apolipoprotein D-like [Hyalella azteca]
MNGFTVVLLLAVAGLTNAYLDQGNCPSIKHVDEFDLPSYMGRWFETQRYPDGIEKGRACVTADYVLVNDTRFSIRNSANL
HDGSIELLHGNAVTTDTPGRFIVGFQGEPPIVYNVLATDYATYSSVYSCAEGEDYREQFAWVLSRTERLDPDALAAALDA
FDSNNIDTSVLQVTPQGGHCVYTSPPPKV
>HaztLCN_XP_018026829 .1 PREDICTED: lazarillo protein-like [Hyalella azteca]
MMSSVSVVVFAALMNIAPVEASFGFGGCPSQAVVDPFIETTFMGTWYEFGKSASLGMLFSRCPKIAYAAGANGLINVTED
WITWFSKGCKTGQMKKANPSKSEGKYSISFWAGAYNGKENNVTAANYNILNTDYSNYAIIWDCFNAFLFHTVSVRVITRE
RVPPQAIIDTIYKDFDSRKISRFGFVQRNQQNCND
>HaztLCN_XP_018022288 .1 PREDICTED: apolipoprotein D-like isoform X1 [Hyalella azteca]
MLKCVLMIVCLLSIAAQAQELSFFKKCPNPSVVSNLDANRYLGKWYENRKYFAIFQFGGRCASAKYSDAGNGLIKVENNQ
LNIFGKPSGVVGQAKFANPSNKEGKLSVSFSSPTPYANSSTAVTNPNYLVLDTDYKSYAIVWNCLSEFFGNIQFMWVLTR
DPQPSEALVNKTLTIIRNKGLDPTRLSITDQNNCK
>HaztLCN_XP_018022294 .1 PREDICTED: apolipoprotein D-like [Hyalella azteca]
MAMHQTGRVIGALVATLMSLASVNGTVFLGSCPNQPVATSFDTTSFVGDWYEYSSYVGLGQVAFRCPKEKYSIIIPGRFN
VEQERIILGVKNTVKGQMKFADPKNLEAKLSISFVEGAFDGRWTEVTPPNYNVLKADPAFIIVWDCTNLVAIHFPTLRIL
TRQPNPPQFVIDEIYKELEDRGISTGLLLKNSLDDCS
>HaztLCN_XP_018012792 .1 PREDICTED: apolipoprotein D-like [Hyalella azteca]
MVKMFSAVLLLAVAGLTNAYLDQGNCPSIKHVDEFDLPSYMGRWFETHRYPDGIEKGRACVTADYVLVNDTRFSIRNSAN
LHDGSIELLHGNAVTTDTPGRFIVGFQGEPPIVYNVLATDYATYSSVYSCAEDHQKQHFGVSYVEGYALNIG
>HaztLCN_XP_018018376 .1 PREDICTED: uncharacterized protein LOC108674908 [Hyalella azteca]
MQIGCARTRCTLRLLAVFLSVLKFTAGLTTQANIEIFNTHKALGQCPTFTPLSTLTKTDIVGIWHVYQQSDNSLENSWTC
KNLVYSAISNDFTYNFRVNNLATSYTGTYGVNTAGAGIISVALRYQLALGADSFGNVNQFVVAADATKYLIIRACTNFGL
FNQQYVYVLTRQTSPDASTITAINTALATAGVQAAQLYTVTQTGCT

>HaztLCN_XP_018008868 .1 PREDICTED: apolipoprotein D-like [Hyalella azteca]
MRSAMGKWMWIAATLHLMTSWALAQVHYREPCPNMPVIKNFDFDRYLGRWYEQERFFTAYQNIGYCWSGTYIKDKYSNKV
SVRLDFEDILLRIPAKIFVDIFRKNAYAAPNRLSYSISGVPREIFVDHYEVSGTDYTSWTIEYSCQNMPFGSLRYAWILT
RSPHPPASVIKKAKAVMVALGIDVYLLERHKNTCVPKY
>HaztLCN_XP_018024192 .1 PREDICTED: apolipoprotein D-like isoform X1 [Hyalella azteca]
MYRLKMSLRIVLLFAVVAAVIAAAAVDPTNLSPPSVADMGWCADLSMVKDFDVDKFLGTWYQQMEVPSPFSEEGQCTEIT
FAKTDAGLNLTSESRSADDMNIKQTFFLSARTRNDTTPYYVMIKDNLPHVEYNVAATDYVSYACTYTCFQIVMGLRVELF
FITTRDPNPPENILNKCNDYFYNNFVDTSKLVPVDHSTNLCDTQWKNNSRPAEEQGNAGLEEREAFEDDIDRGLRSDDAF
ERYEAESKVVMTLLLGESEVETDHIHSSGSSQARCSLHCIEWLLTSMAIILLNTKIVV
>HaztLCN_XP_018024194 .1 PREDICTED: apolipoprotein D-like isoform X2 [Hyalella azteca]
MYRLKMSLRIVLLFAVVAAVIAAAAVDPTNLSPPSVADMGWCADLSMVKDFDVDKFLGTWYQQMEVPSPFSEEGQCTEIT
FAKTDAGLNLTSESRSADDMNIKQTFFLSARTRNDTTPYYVMIKDNLPHVEYNVAATDYVSYACTYTCFQIVMGLRVELF
FITTRDPNPPENILNKCNDYFYNNFVDTSKLVPVDHSTNLCDTQWKNNSRPAEEGNAGLEEREAFEDDIDRGLRSDDAFE
RYEAESKVVMTLLLGESEVETDHIHSSGSSQARCSLHCIEWLLTSMAIILLNTKIVV
>HaztLCN_XP_018012793 .1 PREDICTED: apolipoprotein D-like [Hyalella azteca]
MNGFSAVLLLAVAGLTNAYLDQGNCPSIKNVDEFDLPSYMGRWFETHRYPDGVEMGRTCVTADYALVNDTRFTITNTANL
QDGSIQRQHGNALTADEPL
>HaztLCN_XP_018028308 .1 PREDICTED: outer membrane lipoprotein Blc-like [Hyalella azteca]
MHTILLRRLATLWALASALCLVGCAWTTPPEGISAVTPFELARYQGRWHEAARLDHTFERGMTDVSATYTPLPDGSVRVL
NRGFDTAKGGWREAEGRAVFIGAPTTGSLKVSFFGPFYGGYHVAALDPDYRWSIVVGPDRSYAWILTRDPRISPALKQDL
TERIKALGIAPEALIWVSHERQEP

>AampLCN_KAF0310838 .1 Apolipoprotein D [Amphibalanus amphitrite]
MASRSSVLPLLLAVLAGAVAVSAHRYGAGKCANVTPMDDFDASRMVGLWYVHRAFSTSSTCLTFNYTLTTTGLEVVETKE
LRALDAVGLDHKYSSVGTLKDNGLPGAYQASFSTNPLKSKYVIISTDYDNYAGVFHCQHWAKLVHRRNVYVLSRSRDIEE
IYIEKIRRRMLVFDLEPEQLESVSHEACTDRADSDFDVRLDGSLFDLRDKE
>AampLCN_KAF0296142 .1 Apolipoprotein D [Amphibalanus amphitrite]
MELLRSACGVLVLAALATAHVNHRSSCPIPTHPMKGFVPNKFLGEWYVMRAFDPDVSCLVFNYTTCGEGCLRVVETKKID
LIDNIGISNVYQTEGTLRIDGSEPSEMTASFTTNPRSALYTVLATDYTSFAAVYTCQNVGLDALPLYHRRDVYLLARDRN
TPVTPEVVEQVSAAMVANMIEDEFAPLDHNSCQSAEEADVSVNVSKIGQRVSSVASTVTEGLSKFASSIAGIFSSSDPDP
ASGSVSPLRRRRGAHF
>AampLCN_KAF0288582 .1 Apolipoprotein D [Amphibalanus amphitrite]
MTDYDRFTFTMLSTLLTVCGLVASGMAACPDIPALDVFDAAQYIGHWYEIQKYPLFGENSDTCGQANYTAEADGSISLVN
RGIKPDGSLDFITGTAVAKDPAHPAELTVHFDQGSVGAYNVIRTDYTASALVYSCVNVLGIKFEYAWFLSREPTMDQAVQ
DEYIKILSDAGSDISRLEVTLQNCGNLF
>AampLCN_KAF0306847 .1 Apolipoprotein D [Amphibalanus amphitrite]
MTSQRRALQLLVVMGVLVPSSGQLPFLGRCPAPPAVHHFNTYHYLGKWYEHSRYFSLASLAGKCTHFIYTDQGYGRIGVE
NQQIKLLTGKLNSVVGVARVVDPHYSGRLRVVATNLPSLGTGPNYIVVDTDYSSYAAVWSCADFKLFNLQTLQILTRHRR
PEPHLVADLRHRLRYYGLNTEKLAKTNQEDCPHGY
>AampLCN_KAF0294801 .1 Apolipoprotein D [Amphibalanus amphitrite]
MLFTLLTVCGLVASGMAACPDIPSLEVFEAAQYAGHWYEIQRYPLIGEVGETCVQTNYTLQADGSVRLENRGRKPDGVID
SITGTARAEDPAHPAELSVLFDEGFQGSYNVIRTDYTASALVYTCTPLGDIILENAWFLSREPTMDQAVQDEYTRILADA
GSDTSQLSVTPQNCGSLF
>AampLCN_KAF0288583 .1 Apolipoprotein D [Amphibalanus amphitrite]
MLSTLLTVCGLVASGMAACPDIPSLEVFDAAQYVGQWYEIQKYPLIGELGETCVQANYTA
QADGSVRLENRGKKPDGSIDSIQGTARAEDPEHPASLSVYFDEGFRGEYNVIRTDYSASALVYACTPLGDISLEYVWFLS
REATMDQAVQDEYIQILTDAGSDISQLEETPQDCGSLF
>AampLCN_KAF0289102 .1 Apolipoprotein D [Amphibalanus amphitrite]
MESSRALRVLSLLVLVAAAAGQVPFLGSCPRPPIVGNFEPQFYLGRWYEYSKYFTVFEVGRKCIQAVYSDAGYGRIGVTN
RSIKVLGGTRSDIRGVAKPVGRPGQAKLRVNFEGVPSFGSDANYIVLDTDYTQYAIVWSCSSLKLFNTQFLFVLTREQFP
SPYLVKHIMKRIRHFGLDTGKLQKTDQKNCPYGH
>AampLCN_KAF0288580 .1 Apolipoprotein D [Amphibalanus amphitrite]
MLSTLLTVCGLVASGMAACPDIPSLEVFDVPQYAGQWYEIQRYPLISELGSTCAQALYVAQPDGSVRLENRGRRSDGSLD
SITGTAVAKDPAHPAALTLLFDEGFQGSYNVIRTDYNASALVYSCTNLFGARIEYAWFLSREPTMDQAVQDEYTRILADA
GSDTSQLSVTPQDCGSLF
>AampLCN_KAF0287821 .1 Apolipoprotein D [Amphibalanus amphitrite]
MSPPRVAVPLALLLTLAALTAAQQRFPGSCPRPPIVQKFQVRRYLGHWFEYARYVTVFQRGLRCSRAEYSDAREGRIGVV
NRAIRIADGSTTEARGQAVPVGRPGEASLRVSFDGQPSRGTEANYNVLETDYKRYAIVWSCTSRGVGEQKNLENEQLLFV
LTRARVPSQRLLRRIMRRLQRLGLNTEKLIKTDQENCSKLDKIIAGKSRKAAILKLCSLKHH
>AampLCN_KAF0302926 .1 Apolipoprotein D [Amphibalanus amphitrite]
MVPLRLPLLFTASLLTINVTCQKVRIGECPDVEPVLNFNATAFSGLWYENRKYVNRFTLGQKCVTTRISSNDDSVLVFDT
LGYEKFFKPVRLRGFALQTYGPDVGAFSVGVPIAEDLSNPNLNVIDIDYDQYAILYGCRTKMGGLSHLEWLWVLSRTPYL
TAPTLTAILLRLSHHGFRPLALHPTEQNFCPDNIPGV
>AampLCN_KAF0288581 .1 Apolipoprotein D [Amphibalanus amphitrite]
MYTRYVNCVYLLYAGQWYEIQRYPLISELGSTCAQAMYVAQSDGSVRLENRGRRPDGSLDSITGSAYAEDPEHPAALTLV
FDEGFKGDYNVIRTDYNSSALVYSCGSVFGVRIEYAWFLSREPTMDQAVQDEYTRILADAGSDTSQLSVTPQDCGSLF
>AampLCN_KAF0303822 .1 Apolipoprotein D [Amphibalanus amphitrite]
MLLPTLLLGCFLLVTDVTGHKYSTSKCPNPNPVKNFDESQILGKWRVEYAVKTTSTCFDMDFRKEGSQLMVYETKEPAAP
EKLSLDVKYGSVGTLASTAQAGYYQASFSTSVISGRFVVLDTDYKTYMVVFHCMQMSILGSRRAVYVLSRDGAEITDERL
KQVRARLPELDVEAGLLAKVSHEQCTTAEAADVDLTGSVNVKDVLKAAKKVVTVGGGILELVQVFGALG
>AampLCN_KAF0291958 .1 Apolipoprotein D [Amphibalanus amphitrite]
MRAVCLLGVLLVMAAGASGRCRRFPAIETFDIDSYLGRWYLQYIYTNEATVPLSQCWSWLYFKDRNDKLRIQTSYVNSLT
NRVTNYQNRLWMKNPSEPSIMRYKQNYFLYNRNEDYQVIATDYANFTIEYQCSGSNVLNRREAVWLMTREQYPHPFVLER
AFAAMIQLRLNDVDMERAYQSCTVRETNRRSDAPRTFADWLTGGMTSRIQRRVQGKTLSGFINWLGL

>AnasLCN_KAB7499004.1 hypothetical protein Anas_03465, partial [Armadillidium nasatum]
IKCGISILVLSCPMSLSYSSFGKDMPECCSLPKLIVKDYVLGVWYVTYQFDTSSTCKVWDLKRSDTNTEQLLLTESRQLY
FLDAVGIDHKNSITAVIDIPNPEVPSKMRIRWPTSALTGKADFIIFDTDYENYLAIFQCDRAGLLHRRDVTVLSRTPDVD
DMFVRRVRRLLETADVGHDALDKISQEQCRKSSSRNWHIDEKLFGLLPSGQTSNLKELSRGVADYDISQIEIIGDGIEQM

SESSGNFRGSMTKGGANI
>AnasLCN_KAB7507166.1 Apolipoprotein D [Armadillidium nasatum]
MTFKLLCVLAIIVASASAHDWGMGSCPSVAPMSNLSIEKFLGLWYVIEQFDTSSTCLTLNFQRTSETSLSITKNRQFYIL
DRAGLDHTNSYSGTLDIPYANNQGLLRVKWPLNAAGKGDYIIFDTDYEKYAGVYDCQQIAFFLYRQSAAILSRTPHLDPM
YTDRVKRRLESFKVDTEEFNVIDHNITCKGKDQTDLNINVDKDTFKNIFSSTADTLKDAANKGSSQEEFRTLHTQLLTEV
KTFTTNEAVKEPAEGVNKPSPHLNDVEILEK
>AnasLCN_KAB7496147.1 Apolipoprotein D [Armadillidium nasatum]
MNSFDVDRFLGKWYIIEVFTATNYVNKCMTMTFERLTDTDFEVIQAREFSLLDKVNIDYRHQYTGTLKMTDANRPSEMKI
KWPSHIIGSATFRVVDTDYDKYALINDCQNLFVVARLSFAILSRERTLDEETVNMLRQKIRDMDIDTSPLNAVDQFSCEG
GSGTGTGTGTGTGTGTGTGSGTGTTGTRTGTGNRDGTDTEDGNSNGTGGGEEFPTDRDNSTEPIEANFLALLDEKEIA
KIKDTYHLAELLNWQVTKEELHNDDHLPDPKTGGKKDKKQ

CHELICERATA

>LpolLCN_XP_022241422 .1 apolipoprotein D-like [Limulus polyphemus]
MNGVRLVFVVFLYVSASLHIVCGRVFKLGSCPNVEVQENFNLNKFIGQWYVIQRFQSSSQCLTQNITVEDGDYYLSENGQ
LLSSDLLGINQVSTHEGKLLVPKKDSPSKMVVDFPLTFITKSFMTNISDPFGKVNYWVMMTDYDNYAAIWSCRRMLLGHL
QNAEILSRSPTLDKLIINKIRGRFENYGIDEHNFSVIDQKDCRDKKKRNGVGISLFLDNIFGRLRI
>LpolLCN_XP_022250524 .1 apolipoprotein D-like [Limulus polyphemus]
MNWIGIALVLVYTTTVSKLVNGHVFKLGSCPNVEVQEDFNLDKFLGQWYVIQRFQTNSQCLKQNVTEENGVYYLSQNRQF
FDTDFIRINRGSTSKGKISIPDKNSPSKMIVDFPLNLFGKVNYWVMMTDYDNYAAIWSCRRMLLGHFQHAEILSRSPTLD
KVILNKIRGRFETYGVDEHQFSVIDQEDCQDQKDRNGVEISLLVDNIFDRFRI
>LpolLCN_XP_022252745 .1 apolipoprotein D-like [Limulus polyphemus]
MMWRVFVVVSMVPILATKVATEIFGLGACPPVKVHPSFDINQFLGKWYVIQSHLTSGTCLQRQVSRAPNGRFYLSQIGST
IAGLPVLGESSRELEIPSGEPAKMALKRPFNIMRPTLDYWVLGTDYDNVAITWTCRNLFFGHLHSLEILSRQSSLDHNVI
NIIENFLHDFNLEPGRLVYISQKNCDHTEERPTLSLSFTNFVDRLFNL
>LpolLCN_XP_013784636 .2 apolipoprotein D-like [Limulus polyphemus]
MKNEVIVLMLICGLCKSQYLAPGNCPRPEVVKNLDLNKFLGKWYEVQKTYTFFERNLRCVNAEYDNVGNGKIILTRIGMD
KDGVSKSIGGRAIVAKLSEPAKMVIEFETSPSIPVNYWILDTDYNRYAVVWSCFNMGPFGFFHSEKLWILSREKYLTVDL
LGAIYKNLSKKGIDWSSLRNINQLDCGISKENDVAPNI
>LpolLCN_XP_013784348 .2 apolipoprotein D-like [Limulus polyphemus]
MKAYTLLCCLLVVTMATSSSRSLLNGECPQPPVKANFELQKYLGTWYEVERNPAIFQSGQKCTSATYTLKDNGKVKVFNK
AVIVKTGDVTSIEGEAYIPDPNEPAKLLVSFPGNPFDGNYWILDTDYDKYSVVFSCQSILHVFRIEYLWILSREKTLPDT
TLTNIYRILDKNGIARNKLIKTDQSDCKTE
>LpolLCN_XP_013785678 .1 apolipoprotein D-like [Limulus polyphemus]
MKTKVLLVILIVRLALTTDGQSLAPANCPRPDVVKDFELSKYLGKWYEIQRTLALFERDLQCVTAEYGILKNETISVRNA
GINSNGEKKGIEGEATVQNISEPAKLKVRFETAPFPANYWILDTDYDNFAVVWSCFNLGPFGLLHTENLWILGREKVLDE
DLLKTIYSKLDKLGIEWDHLRNTTQIGCDISGNNK
>LpolLCN_XP_022237321 .1 apolipoprotein D-like [Limulus polyphemus]
MQKFDSAKFSGKWYEIEKSFEFSDIGSSCVRWEIKNGEEGVYSAVGKGVGLLGNQLKVTGTLKTPNASEPGKMRMHYDGL
PYVENYWVVNTDYDQFAITLSCLELIPNLVFRDVLTILSRKPSMDATLKSQIYDFLISNNINQYDLFLIDQEDCSIL
>LpolLCN_XP_013778512 .1 apolipoprotein D-like [Limulus polyphemus]
MRSLLQNLIFVFCVLGPVGVFPQVPGFGQCPTHPVMKKFDYRKFLGEWYEVARQFTWFELGWKCVYTNVTDLGGKVLGFM
DRAVTIFDAKLFFDGKTKPLDPKEPAKLVFKPFDKLPFRMKLWILDTDYKEYCIFWTCVDFLKVSHAENLWIYSRNKTIR
QSTKVKIIQFLDKNKVNRYGLKHTNQTNCPW
>LpolLCN_XP_022246664 .1 apolipoprotein D-like [Limulus polyphemus]
MYIGKWYEIERSFAIFEMGLKCVTSEYTLNEDGTVKVINRGVKMLRKSSTVNIKSKYWVLDTDYKQYSLVISCNDVPRNM
FHFEILWILSRKPTLDGDILTRIKNDLESRGIDVAGLKKTEQDCFK
>LpolLCN_XP_022237967 .1 apolipoprotein D-like [Limulus polyphemus]
MRLKYEALPYIEKYLVLDTDYDNYAITWSCLELLPKRKFREILTIFARKPKISQELQKFLNDFVVKRHINTILLTGINQD
NCPES
>LpolLCN_XP_022255402 .1 apolipoprotein D-like, partial [Limulus polyphemus]
FYNGIWYIVEGYPNRFYDDSVCHEWSLATDGKYVYINETVFNVKKHDFDTLQGKARYQSKGGVNCLVLSPGIKSLPYGAD
MCIISTDYSNYGVMYVCRPFKGNKKQEYIYIMSRTPTLEPRLRARVLIDNIVRGGFQVTPFAPMNQLYCPLP
>LpolLCN_XP_022253346 .1 apolipoprotein D-like, partial [Limulus polyphemus]
LNSGEIIIGVEGEAYPPDPEVPAKLKVEFPTSNSTYNTGKYWVLDTDYNTYSVVYACTSIAGVFRSESCWILARQRTLPQ
ETTNKLFNFLDTNGINSNKLIMTNQTNCD

>AvenLCN_GBM40656 .1 hypothetical protein AVEN_111284_1 [Araneus ventricosus]
MSVGLIAVLTLSVIAGCYGNTFKMGACPRVVVMDQLDFDRFSGDWHVIQRFNPMATCTKFAIEKGPDGVYTVNETSRPLG
LNFGFHQQYIKARKINFLRNDTTSIFRLERNLVHFTLSTFGVVDTDYDKYAIIWGCDPVLFGSVQNVDILSRQTSLDSDT
IKKAKDTLKEMHIDTSPMDNVDHSHCGNSGNGDGAEGEGNPNTNNIVAG
>AvenLCN_GBM56015 .1 Apolipoprotein D [Araneus ventricosus]
MNPAFIACFLLCAAHFASAQFPYLGKCPTPDVKENFDLEKFSGRWYEIKRTMSVLEIGARCVAVNYTDAGYGDGSIEVIN
EGVSAVLKLRKSVRLIATLPDKNEPAKWSLRLSDLSLKTDYWILDTDYDNYAVIWACNHIWLTFKYARTENLWILSRERT
LPEETLDKIHEKLNALKDIKSRKLLRKVNQEKCD
>AvenLCN_GBM98442 .1 Apolipoprotein D [Araneus ventricosus]
MQLLLLGLAALVGFASAQKVSVGGCPDVPIKENLDVKQYVGKWYEIEKNPVPFEAGLKCNEANYGDEGDYVSVVNKGVSK
RTGKESSVEGKATIPDKNVPAKLKVKFNGMPFSADYWVLDTDYKQYSVVFSCFSVLNLFNAEYVWILSRTPTLDENIKQN
IYKFLDENKIDRSKLSATVQDC
>AvenLCN_GBM98444 .1 Apolipoprotein D [Araneus ventricosus]
MQLLLLGLVAFFTFLVDSNAQKIKTGKCPAIPVKKNFNVKEYAGVWYEIEKNPTSFEFGSKCTNAVYGDEGGYLSVTNRG
VDIKTGKGRSIHGKVTIPDKSVPAKLKVKFDAVPRIGDYWVLDTDYKQYSVVFSCVSVDEALKIEYLWILSRTPTLDENV
KESIYKLLDKRKISRDNLSPTIQDC
>AvenLCN_GBM41520 .1 hypothetical protein AVEN_32326_1 [Araneus ventricosus]
MWAQILLLCASATVGLCALGSCPTPKVQADFELEKLSGTWYLLEATASIDRVSKKCSQFILDKKTATKSSFLHKFISTVS
DKWKSEYSELTTPKKDEPAKLLVNPLKGTIMARKWPLWVIDTDYDTHLILYSCHKLVLAYTEEVFILSKTPKLDDKKKTE
LYDLLQKRNLSQKTLVEVNQNEKDCKE
>AvenLCN_GBM98448 .1 hypothetical protein AVEN_114080_1 [Araneus ventricosus]
MAGHRNCHLVTCAAIFSVYLVCYVHAQTLLLGACPSPPMQSNFSAQDFLGRWFEVERTFVMAEIGWRCISVDYKEESGRI
RVETASALP
>AvenLCN_GBN95069 .1 hypothetical protein AVEN_188064_1 [Araneus ventricosus]
MVPDFASGVWHLDDIKETGFLLSITCITVNVKDLTSVSSRTFDKAAAPSLVPDFASGVWHLGDIKESGFLLFITCITANI
KDLTSVSSGTFDKPAAPDFGISVELKKKISSCSLPVVNQ
>AvenLCN_GBL95581 .1 hypothetical protein AVEN_24796_1 [Araneus ventricosus]
MLRPTFVLLFGLVPLVCGQLIIGFCPPLHNYVKNLDFEKYSGTWYVVAQSRFHPMQALNCQRETYAVKRNGLSVTFNTSK
KYEHQQKYKNGTLKQEGDNKGEMKLSLNEYKLDILEREKEKAKKIYGNQPNGLWWLEFRLDRNRIEKRWSLKSRFRGTLK
EASEKNNALRRLKPRFEGSIN

>PtepLCN_XP_015904925 .1 apolipoprotein D-like [Parasteatoda tepidariorum]
MQSLGVFAFVLCIVAGCHGNTFKLGACPRVVVMNNLDFDQFLGEWYVVQRFNPMATCTKILYNKTSDDSYTISEKSRPLG
LNFNFHYQFIKPRHLNFLRNDTTSIFRAERDIAHFTLSTFGVIDTDYNNYAVVWGCDPVLFGSIQNVDILSRTPTPQTNL
IKSVKETLKNLSIDYHPMDNVDQTRCEDSNSNSDGSSTDGDRNTNNIIAG
>PtepLCN_XP_015904924 .1 apolipoprotein D-like [Parasteatoda tepidariorum]
MKILAVLLKTFFIIALCRGSDFKYGGCPKVVVMNNLDFDRFSGIWYVIQRYSPKATCTKIIFEKAPDGTYTMNETGRLLG
LNSNTFDTKTRKIEFLRNDSRSVFRAEKNITHFTLSTFGVIDTNYVMYAIVWGCDPFLFASVQNIDIMSRIPIPSKDLVK
NAERLLKDLSIDYSSMKNVDQSNCTELPSTTTSSSKDTNNIVIASNPPERTEKQ
>PtepLCN_XP_015919070 .1 apolipoprotein D-like [Parasteatoda tepidariorum]
MWAAQILLVCLSATVALAAIGSCPTPKVQADFDLEKFAGTWYLLEASAAVDRVGKRCNTYIVEKKERNHASLLHKYMSSV
TEKWKSEYSDISTPKSKEPAKLSVTPLKGTILARKWDLWVIDTDYDSYAILYSCKKLVLAYTEEIFILSKTRTLEDSKKT
QLYDILRKRDLNQKNLVTINQNEKDCKE
>PtepLCN_XP_021000965 .1 apolipoprotein D-like isoform X1 [Parasteatoda tepidariorum]
MKTFLPVVCFVLIFAGVFAQEQKPTSCPGNVKVMQNFDVNQYLGLWFEIEKTPMVFEAGLKCITANYTDKGNYISVFNRG
VSRRSGREKTSEGKATVPNSDEPAKLKVRFDILSFSSDYWVLDTNYDEYTVVYSCFNMLKGYETEFVWIMSRSTTLSEAT
KDKAYKLLKANNIDTENLRTTDQDC
>PtepLCN_XP_021000969 .1 apolipoprotein D-like [Parasteatoda tepidariorum]
MQQILFLTLIVVTIGLVTAQRVSGGACPNVPVKQNFDVSKYAGVWYELEKYPVPFQAGLKCNTASYTAKGDYLVVVNYGI
NKRTGRKHSIEGKATIPDKNVPAKLKVKLNTMPFSANYWVLDTDYDEYSLVYSCHSVLRLFKTEFVWILSRETALEETAK
ENIYKLLDDNNIDRKTLVHTDQDC
>PtepLCN_XP_021000966 .1 apolipoprotein D-like isoform X2 [Parasteatoda tepidariorum]
MKTFLPVVCFVLIFAGVFAQEQKPTSCPGNVKVMQNFDVNQYLGLWFEIEKTPMVFEAGLKCITANYTDKGNYISVFNRG
VSRRSGREKTSEGKATVPNSDEPAKLKVRFDILSFSSDYWVLDTNYDEYTVVYSCFNMLKGYETEFVWILSRSPTLSETT
KDKAYKLLKANNIDTENLRTTDQDC
>PtepLCN_XP_015910770 .1 apolipoprotein D-like [Parasteatoda tepidariorum]
MFKSLYLVLYVCLLFVTVGFSQRISAGECPDVTVKKDFDVSKYVGQWYEFEKNPAFFENGLKCNWAIYRNEGDYISVNNS
GVNVNTGVRTSINGKATIPDKNVPAKLKVQFDTSPKPGNYWVLDTDYEKYSVVYACSSIEHMFKAEFLWILSRTPVLDDK
TKSVAYDVMDRNSVSREGLFPTVQDCYGH
>PtepLCN_XP_021002786 .1 apolipoprotein D-like, partial [Parasteatoda tepidariorum]
MQRIFILAFVAVTVGVVTAQKISGGGCPDVPVKQNFDVNKYVGVWYEIEKYPMPFESGLKCITANYTAKDDYVVVVNAGI
SERTGKVQSIEGKATIPDKNVPSKLKVKFNYMPFKSNYWVLDTDYDEYSVVYSCFSVLHLFKT
>PtepLCN_XP_015920996 .1 apolipoprotein D-like isoform X2 [Parasteatoda tepidariorum]
MGRCGYYSVVLFAWAFVFIVKVQTQTLLLGACPNPPMQQNFSTQDFFGRWYEVERSFVMAELGWRCITVDYKEEGNRIKV
ETAGQAVVRRTMNALATFSPSNPARIILRGEGSLPTQSTNYILWTDYENAAVVWSCRNVEPPIPISGFDFLRNLSHTENL
WILSRQRTLSPEIKEQIYSVLDTNAINRRSLRPVPQDNCQAAANPTE
>PtepLCN_XP_015927987 .1 lopap-like [Parasteatoda tepidariorum]
MAPKTRIILIKAMLYPATCPDYNVMNDFDIEKFDGVWYENQRTFALAEGGLKCAKTVISGANKERLRASFRRTGVNILNR
KVTLMRGYLHTPRGEAPAKLELVMGPLQLFLQYHVIDTNYDDYAVIWGCFESPKYSPILGHTENLWILSRNKTLSEEHKE
TIYNKLDTLNINRAGLVETTFENCTSSKYS
>PtepLCN_XP_015911791 .1 apolipoprotein D-like [Parasteatoda tepidariorum]
MLKLTAVLVAVVISCVTAQRISPGGCPVVVVKDGFDASQYVGVWYEIEKNPAVFEAGLKCNQANYTAEKDYIRVVNTGVS
TLTGKKVTISGKATVSNKNVPAKLKVKFDSMPFTADYWVLDTDYEEYSVVYSCVTTLNLFKAEYVWILARDAALQEETKN
KVYKILDNNKISRQSIVATTQDC
>PtepLCN_XP_015911794 .1 apolipoprotein D-like [Parasteatoda tepidariorum]
MNGIISIVLVVVCFNLVSTQKISFGQCQNVQVQQDFRADRFTGLWYEIEKYTVPFEIGLKCVTANYTQESDHLKVVNAGI
FKWINMRTSLEAQLNTPNSQEPAKMQLTASF
>PtepLCN_XP_015930076 .1 apolipoprotein D-like [Parasteatoda tepidariorum]
MKYLSIVLLVLAKLVSSLRPLKVDMCEHPPLMKDFDYRKMGGRWYEFARLPNPFQYLMPQRCNTQYMTVTDGGLNFNTSM
IEESTGFEINFNGGLFPYPEAPYHMKFTIEMVGLEDWVDIVDAKYDEYVILYHCKPIGAFRFAFGWILTREKELSEEKKE
RLLKKVKSYNLGPRLEYESQKKCYD

>SmimLCN_KFM71663 .1 Apolipoprotein D, partial [Stegodyphus mimosarum]
MSVMLVLAVVLSVLAGCHANSYRMGGCPRVTVMKSLDFDRFSGDWYVVKRFNPMATCTKISIEKGPEDVYTVNETSRPFG
LNLGFNNQYMKARKVKFLRNDTNSIFRVDRNYAHFALSTFGVVNTDYDNYAVVWGCDPVLFGSIQNIDILARDPKPSADV
LTSAKNVIKEMGVDSSPLENVHQSGCQPASQS
>SmimLCN_KFM69602 .1 Apolipoprotein D, partial [Stegodyphus mimosarum]
MFHLAFSVVLVAVLYGQALTQKVDVGGCPDVKVKEPFDVSKYVGEWYEVEKNPTPFEAGLKCNKAKYENKGDYISVVNTG
ISERSGKESTIEGKATIPDKNVPSKLKVKFNNMPFKADYWVLDTDYENYSVVYSCFSVFKLFNAEYLWILSRTPTLEDAT
KENLYKFLDDNKIDRTRLK
>SmimLCN_KFM62833 .1 Apolipoprotein D, partial [Stegodyphus mimosarum]
MLVKLAVLCLTATVCWAGFGTCPAPKTQSGFDLDKFMGTWYELEATPGKDSLTKCSTYVLEKKEKRKAALLHKYISPVTG
KVKSSFSEISTPKSSEPAQLLVSPLKGTIMSRKWNLWVIDTDYETYAITYTCHQVLLNFYEEARILSRTKTLDDKKKMDI
YAIMNKNNLPQKSLIEIDQQ
>SmimLCN_KFM79274 .1 Apolipoprotein D, partial [Stegodyphus mimosarum]
MAFFRVALVLFFVCVIKGVTGQFPYLGKCPSPEVQENFDMEKFKGTWYEIERTMSFLEIGAQCVSTNFSDAG
>SmimLCN_KFM74144.1 hypothetical protein X975_07331, partial [Stegodyphus mimosarum]
MTLCFLKRSADDIIKKEMKSCLIFLLSLPLILGQLVLGPCPKLQEFVQDLDYNKYSGKWYVVAQSRRHPMRALECQTVNY
TQNSNGLDVEYNTAKKYLHMKNFDGGLLKRESGKSSGELSLFLPHFPQRLQFRVLKANYSEFAVEYVCLPGVFNYTSSVT
LLHRKPQATLQNLELALQPLNIQKHVNPHIGNPLIPVNHTNCNQ
>SmimLCN_KFM73870 .1 hypothetical protein X975_10357, partial [Stegodyphus mimosarum]
MGIKNTFRIHILVIFAISIAWSLSLYPASCPEPKVQENFDIERFSGLWYEVQRTFALVEGGLKCATTVVSGASNKKMRAT
FRRNGINILNRRITLMRGYLHTPDAKAPA

>TclaLCN_PRD34593 .1 Apolipoprotein D [Trichonephila clavipes]
MKFRILIAVCFVLCTERLAFGQFPYLGKCPEPKVQEDFDMQRFQGRWFEIERTMSFLEIGSQCVTTNFSDAGYGDGSIEV
ISEGKGPVLKLHKSVRLIATVPDEKEPAKLKLRLSDLSLKTHYWILHTDYDNYAIIWACNHIWLTFKYARTENLWILGRE
RTLPEETLDKLHQRLKTVKDIRVAKLMKKVNQENCD
>TclaLCN_PRD36028 .1 Apolipoprotein D [Trichonephila clavipes]
MIYFLEEIRYSLPEVKLVIAVFEFTFSKYCKSEYVGEWYEIEKNPVPFEAGLKCNKAKYGDEGDHVSVVNQGVSVRTGKP

SSIEGKATIPDKDVPAKLKVKFNGMPFSANYWVLDTDYKQYSVVYSCFSVFNLFNAEYLWILSRTPTLDDSIKENIYKLL
DEQKINRKRLQPTVQDC
>TclaLCN_PRD36027 .1 Apolipoprotein D [Trichonephila clavipes]
MRSFLGRWYEVERTFVMAEVGWRCITVDYREESGRVRVETSGNAVVKRTMAAVATFTPSTPAPLPTQSTNYVLLSDYNNF
AVVWSCRNVDPLIPITGLDFLRNLSHTENLWILSRNRTMDATTKEKIYSFLDTNAINRRSLRPVPQENCQEGTR
>TclaLCN_PRD33386 .1 blc [Trichonephila clavipes]
MRIYWLLLLLLLAGCTSVPDKVTPVSPFELERYLGSWHEIARLDHSFERGLTQVTAEYSLRDDGGISVLNRGYDAAKERWK
SATGKAYFVDDTNTGRLKVSFFGPFYGGYNIAKLDPDYQIALVVGPNLDYAWLLARKPQLSAAECAPYLAEAERIGIALD
QLIWLAPCR
>TclaLCN_PRD37252 .1 blc [Trichonephila clavipes]
MPFIKTFPKASWRLAKIAVGGVVLTGLAAGTIAYAQTKPLATVDKVELDKYLGVWYEVARKPMFFERKCAYNVSATYTLN
ENGNIVVDNKCYDKDGNLQQSIGEAFVVNAPFNTKLSVSFLPEGVRWVPLARGDYWILKLDEEYQTVLVGEPRRKYLWVL
SRTPNPKKETILEYLNYAKSLGFDVSDVIFPDHR

>TurtLCN_XP_015793556.1 apolipoprotein D [Tetranychus urticae]
MKFVLITAILCYLQTSSLAQRFVSGPCPVIDPPENFQKQKFFGHWIETEKTPSMFDLMMRCMTVEYSDDK
DGSIDVAVKGVSLGGIPVSISGDGLIQDINRAGFYNVRYGLGMPMQGTQVTVVDTDYNEYALIYSCTNSL
LSGLFHSEYIWLLSRDGSLSNPTRQNIYEKLDNLKINRSGLQLSDRTGCPSNVTREGDADLAAVQTTQLP
IVQ
>TurtLCN_XP_015783199.1 apolipoprotein D-like [Tetranychus urticae]
MKMFATLFVVFAIASSALGECPVPPTVPGADIGKIAGRWYEIAGPTRNFTCMTWDYTPREDGNFNSTTLS
TLPDGSKDAEYMLAERADDQNLLNLYAMSHRTPYPITFYIADTDYDNYLAAYTCVVVPWRSDILSGLILS
RTNTMDAEKYAELTDLLVNKIGVSSDVVGPITQKDCKYWPVPQ
>TurtLCN_XP_025018076.1 uncharacterized protein LOC107369238 [Tetranychus urticae]
MKQFFALCVFAFSFASVFAIECQECNRSVKTSIQPGNCPEPNRLPQPFDLNQYLGIWYVLKSTKPYFEDG
LRCLQADYKFDTTTGTFVTNSTGFDIFNETVSRVQFAKSYVQDNVFTAKYSLTKFYKI
>TurtLCN_XP_015788860.2 apolipoprotein D [Tetranychus urticae]
MKHISISYSTSFINKHSFNFSCRNYYNMKMIATLILVFAIASSTLAQCPVPPTVPVSDVAKIAGRWYQIA
GPTFSGTENQPKCVTADFTPKKDGNFNLTMNATDPDGSKNSGVGLVERTDNQNLLKLHTSPYTTSSSINV
YLVDTDFDNYLAAYICFLDPSQSPVQTGVILSRANTVDAEKYAELTDLLINKAGVSTDYFIATNQKDCQY
>TurtLCN_XP_015783651.2 apolipoprotein D [Tetranychus urticae]
MEMIVPLFVVFAFASSALGQCPVPPPVPAVDMSKIAGLWYEMAGLSAVGFTEAYLCTTDEFTHREDGNFN
FTIRGTKLNGVNITDYGLTERTDVQNLLNIYTPPDSPYPLVFYVADTDYDNYLAASFCYCVPGIFTGKGG
MILSRTNTMSADKYNELADMLVNKIGVPRDYIEPTSQKNCKY
>TurtLCN_XP_015783728.2 apolipoprotein D-like [Tetranychus urticae]
MILNTYASVNQQFSLQTFNFLICAKTMKMIATLFVVFAVASSAFGQCPVPTPVSGADIGKIAGRWYEISG
STLDLTCVTMDFTPREDGNFNYTTLGSKSDGSKHSRHLLAIRTDNENSFNISDMSTETRYPITFYIADTD
YDNYLAMYSCVFLPGYSQIQLGFILSRFNSMKDDKLAELTDLLIKLGLTDDKIQSISQEYCKYWPASQ
>TurtLCN_XP_025016706.1 uncharacterized protein LOC107368188 isoform X3 [Tetranychus urticae]
MKMYVFVLLLSVLVAVGQSTKIDGPCPIKIKQIETFDPNRYLGKWWDVYANSYGVCGSGYGCQLTYYRLY
HEDIHAIPINWFTGPTYSNECSFVIYQTDYDVDPRLFYDGDDNSIYVLDTDYDSYAVEIGCANDDGVTHE
ERVVLRSRNFVMSDEVRDKLVKKVVDYGFAEPNLNAHNFTGCKDFTPDF
>TurtLCN_XP_025016705.1 uncharacterized protein LOC107368188 isoform X1 [Tetranychus urticae]
MIAMKMYVFVFVLSVLVAVGQSAKIDGPCPIKIKQIETFDPNRYLGKWWDVYANSYDVCSSGYGCILTYY
RDYDEKLHAIDVNYYFGPDYANICSFGIWQTDSDVDPGLFYDGDKNSIYVLDTDYDSYAVEIACINDDGS
THEERVVLRSRNFVMSDEVRDKLVKKVVDYGFAEPNLNAHNFTGCKDFTPDF
>TurtLCN_XP_025015913.1 apolipoprotein D isoform X2 [Tetranychus urticae]
MNKLALISLIVLSSTLSASALPNIFNLKALQDKIPQLSAVGSSFWVGRCPQTTEIKGGFNLTQYLGKWYE
IKRTDLIFELGLRCVKTEYTVESNGNVAVNNTGVSLINTQVSVVGTAKPGTQSNVFAVSFFQFSPSAQYW
IVDTDYETYSLVVSCNDVLGLFNLKNAWILSRKPNLDSDIVDQLVAKLKAIGVETDKLTTTAQDCGN
>TurtLCN_XP_015794145.1 apolipoprotein D [Tetranychus urticae]
MITKGYILLLCFIASVCCEVTLPGPCPNIPAPANFSWKKMEGSWYVLKETKDENGDLENCPVDTYIFRDN
SSVTYLNGFHLGGYTRGEGGPGLIDQSQPNKITIKFTPTGKSFDRYVVDTDYENYAVLIGCKEKKGNHLI
HAKLLTKSQTPPAQSKLAAYIAVQNLGVPVTSLINNYDQYSYCNGYPDSKANRGKSQS
>TurtLCN_XP_015792804.1 apolipoprotein D-like [Tetranychus urticae]
MKQFLALCVFAFSFALVFATGCEECKKSVKTSIQPGKCPEPNRLPQPFDLHQYLGTWYEIKSTKPYFEDG
QRCIKADYKFDTTTGIFVTNSTGFDSVNKTVSRVQFAKSYDQDNVFTAKYFLGSGYYLTTQYWVVDTDYN
NYALVVSCNDLFGLASSRDVWILSRKPTLDDDIVTNLVAELDTVGIHGSKLGDMIQNC
>TurtLCN_XP_015792803.1 apolipoprotein D-like [Tetranychus urticae]
MKQFLSLCVFAFSFALVFATGCEECKKSVKTSIQPGKCPEPNRLPQPFDLNQYLGTWYEIKSTKPYFEDG
QRCIKAEHKFETTTGIFVTNSTGLDSVNNEVERVQFAKSYDQDNVFTAKAFLGSGYYLTTQYWVVDTDYN
NYALVVSCNDVLGQFNFRDVWILSRKPTLDDNIVANLVAELDTVGLHGIVLGDIIQNC
>TurtLCN_XP_015792802.1 apolipoprotein D [Tetranychus urticae]
MKQFLSLCVFAISFALVFSTGCEECSKLVKTSLQPGNCPEPNRLPQPFDIKQYLGTWYQIKSTKPYFEDG
QRCIKADYKFEATIGIFVTNSTGLDSVNNEVERVQFAKSYDQDNVFTAKAFLGSGYYLTTQYWVVDTDYN
NYALVVSCNNLLGLDNSRDVWILSRKPTLDDNIVANLVAELDTVGLHGIVLGDIIQNC
>TurtLCN_XP_015792521.1 apolipoprotein D [Tetranychus urticae]
MKQFFILCVFAFASASAFVIPSLTLDPLQQIKRIVPEIFNLTDLRVDLDDIKSSISPGKCPEATELPEPF
KLNSFLGTWYEIKRTGQIFENGLRCVQANYKLDQAAGNVIVNNSGVNPKGKPVATIGTAATTDKSNVFAV
KFFPLSPSAQYWVVDTDYTGYSLVVSCNNVFGFFNINDAWILSRKPSLDNEIIQKLEAKVIELGFTKLRF
TDTPQDC
>TurtLCN_XP_015791047.1 apolipoprotein D [Tetranychus urticae]
MKNLAFVLYLSSAILVANCFPDLFSDLTRFTQTEIDLWKSLIPNVKAFPDALKVLSLGGCPQPENPPSDF
DFRDLLGLWYETERTALFELLETGQKCPQITFSNDENNDLKVNMTVISSLLNTPVTSIKSAKINSNQPNV
FSMRYSLLASDIQYWVVDTDYDNYALIFACKQSLFLSIRNAWILGRKATLDASIRDNLVSKLSKLGVPET
SLIKSDQSCSTFVPIQ
>TurtLCN_XP_015789489.1 apolipoprotein D [Tetranychus urticae]
MKMIATLFVVFAFASSALGQCPVPLPVPAVDLSKIAGRWYEIAGLTAGEFIESRACTTHDFTPREDGNFN
CSFGGIKTDGSKFSDIGIAKRTDIPNLLNLNSLTEMVPWPINFYVADTDYDNYLAASFCMDIPQLLITVR

VGMILSRTNTMSADKYAELTDLLVNKIGLPSDSIQSTIQKDCKYWPVSELQQSEEIEYSPFN
>TurtLCN_XP_015788858.1 apolipoprotein D-like [Tetranychus urticae]
MKMIATLFIVFAVASSTLAQCPVPPTVPVSDVAKIAGRWYQIAGPTFSGTENQPKCVTADFTPKKDGNFN
LTMNATDPDGSKNYGVGLVERTDNQNLLKLHTSPSTTSSSINVYLVDTDFDNYLAAYICLLDSSQSPVQT
GVILSRANTMDAEKYAELTDLLINKAGVSTDYFIATNQKDCQY
>TurtLCN_XP_015786723.1 apolipoprotein D-like [Tetranychus urticae]
MIATLFVVFAFASSALGDCPVPPTVPGADIGKIAGRWYEIAGPTAHQISPNLTCITEDFTLRKDGNFNLT
VLSTFQNGSKHVEYGLAGRTDAQNSLNLYPMGYKTPYLLTFYIADTDYDNYLAAYACYTDAGLSTIQGGL
ILSRTNTMSADKYAELIDLLVNKIGVSSDSVGPIVQRGCKYWPVLQ
>TurtLCN_XP_015786722.1 apolipoprotein D-like [Tetranychus urticae]
MIATLFVVFAFASSALGDCPVPPKAPGADIGKIAGRWYEIAGPTAHQISPNLTCITEDFNLRKDGNFNLT
VLSTFQNGSKHVEYGLAGRTDAQNSLNLYPMGYKTPYLLTFYIADTDYDNYLAAYACYTDVGRSTIQGGL
ILSRTNTMSADKYAELIHLLVNKIGVSSDSVGPIVQSGCKYWPVLQ
>TurtLCN_XP_015785538.1 apolipoprotein D-like [Tetranychus urticae]
MNQFFVLCVFAFAFAPTFEARGLFQSLTGSVKPGKCPEPTRLPQPFELKQFLGTWYEIKSTKPVFEKDLR
CVQANYQLDSATGNVVVNNSGVYVSNNTIFTVGTAKTTKQDNVLSVKFLPYSPAAQYWVVDTDYKGYALV
VSCNNVFGLFNFRDVWVLSRNPTLEDATVKNLVAKLDSVGIKDTQLFDTLQNC
>TurtLCN_XP_015785531.1 apolipoprotein D [Tetranychus urticae]
MKQFVILCVFAFSFVPAFEAAGLFQLDSIKTSFLPGRCPEPARLPQPLDFARYLGEWYEIKSTKPIFENG
LRCIKANYFVDTYPVIFVNNSGVDLANNTIFSLGAAAPTQQDNVLEVKFFKYSPYAQYWVVDTDYDNYAL
VVSCNNIFPFFNPRDAWILSRKPTLDDDTVKNLVAKLDSVGIKDTEFLDTVQNC
>TurtLCN_XP_015785530.1 apolipoprotein D-like [Tetranychus urticae]
MKQFFVLCVFTISFAAAFDAADLLQHDSSRKSIKPVDCPELTRLRQPFDLKKYLGAWYEIKSTKPIFENG
LRCVKTIYGMDNFQIVFVNNSAVDLANNALFSLGVASHLENALSVHTFFSNGLSDSPYSQYWVVDTDYDN
YALVVSCNAVAPLFTIRDAWILSRKPTLDDDIVKNLVAKLPSFGYEDVELLDTAQNC
>TurtLCN_XP_015784418.1 apolipoprotein D [Tetranychus urticae]
MSNKIFFILLACYLGSISCEIFDIGTCPTPTTFSPSDAAKFYGKWYEIARVESGTEKGLRCVTFDIASNA
SYLSGHPVTQTGFDDKNNEYIYIGNLQMWDDQTVFEYGNFDILDIDLAYGYTVVDTDYDNYAIVLGCSEL
PPFGYWFPFLLVRIRVAWILSRTPTLDAELQSKLVDLLAKNGVGQSKLTFNKFDSCPSYY
>TurtLCN_XP_015783727.1 apolipoprotein D-like [Tetranychus urticae]
MKMIATLFVVFAFASQAFGQCTVPPSFSCPNITKIAGRWYEIAGPIHDLACVTMDFTPREDGNFNYTIIG
AKSDGSKFSQHLLATRTGLENSFNVSDMSTNTTSQITYYIANTDYDNFLVLYACVYLPGYSNFLSGSIFS
RTNTMSDGDVYKLKNLLVDTYGVPSDSLVAITQKGCKYWPVF
>TurtLCN_XP_015783725.1 apolipoprotein D-like isoform X1 [Tetranychus urticae]
MIFTLFVVFAVASSALGECPVPPTVPGADIGKIAGRWYEISGSQLGGSQERVTCLTQDFTPREDGDFNFT
MLGTQHDGSMNIEQLLAIRTGSQNSIDLNDMAKRLPYQVTLYVADTDYENYLATYSCIHIPGYSNIIYGW
VLSRTNTMSDEKYAELNDLLVNKVGVPSDVFGPIIQKDCKYWSLSPQ
>TurtLCN_XP_015783652.1 apolipoprotein D [Tetranychus urticae]
MKMIATLFVVFAFASAALGQCPVPPPVPAVDISKIAGLWYEIAGFSAIGFIDSYTCTTEEFTPREDGNLN
ITIRGNELNGSNVTDYGLVKRTDKQNLLDIYSLSPEHEENPYPIIFYVADTDYDNYLAASFCYYIPGLDT
AKGGMILSRTNTMEADKYNKLVDMLVNKIKVPRDYILPTSQKDCKY
>TurtLCN_XP_015783405.1 apolipoprotein D [Tetranychus urticae]
MQLLFVSIWFLNIAINVNSQNLSPGSCPSLQPPSGFLLPKYLGKWYEIARTDNIWEINLKCVTADYSLNE
DGTVKVDNQGFNPRDKLSKAIGTARLTESSNLLKVKFSRFSPEAPYLIADTDFNNYAIVVSCVDVFKAFK
FESAWILSRYPTLPKDTLDRLFNDLETKFGVDKKTMQLVDQSNCPVRDKQ
>TurtLCN_XP_015783357.1 apolipoprotein D-like [Tetranychus urticae]
MKMIATLFVVFAVAASALGECPVPPTVPGADIGKIAGRWYETTDAFSHNSTFITFDFTPREDGDFNLTIH
APFSKHDEYVLAKRTDNQNVLNFIDVYHKTRYQVTFNIADTDYDNYLVAYTCLVYSSHSALDLPMILSRT
KTMSAEKYAKLSDLVDNEIGVSRDKIEPVIH
>TurtLCN_XP_015783355.1 apolipoprotein D-like [Tetranychus urticae]
MKQFFVLCVFSLSFAPVFETRDLSQHNAIRTFIAPAGCPEPTRLPQPFDLAKYLGDWYEIKSTGPIFEYG
LRCTQFNYKLDNATGNIAVNETGVIFSGQKFSSLGVAVPSAQDNVYSIKFPNYATYGQYWVVDTDYTNYA
LVVSCYTAFSHDVWILSRKPTLDDGIVEKLVAKLDSVGIHNTKLSETIQNC
>TurtLCN_XP_015783285.1 uncharacterized protein LOC107361069 [Tetranychus urticae]
MKMIATLFVVIAFSSLAFAESGYIPCPVPPTTPGADIDKMAGLWYKTNGAPYSINLQVYFTPREDGDFNF
TIRSPYGGKDWEDAIAERTDNQNILNLYNGMSKEIPSFMTLNIVDTDYDNYASVYLCWSFVPGTSFVGEH
MNLSRTDYMAADKYRELANHVFDKAA
>TurtLCN_XP_015783283.1 apolipoprotein D [Tetranychus urticae]
MKSIILISALIGCCFAATPKIGKCPTPGDEKTDFEVSRYLGRWYEIKRSDTPSEKGVKCSTANYSLRSDG
KINVTNSGVNSEGDNVVVYLTASIVNPDQPNFLSVEIFPGAPTASYWVIKTDYETYSSLISCVQITADMY
SLSGWILSRTPTLDVALVDELTKLLETKGVTELLTNDRTGCTN
>TurtLCN_XP_015783221.1 apolipoprotein D-like [Tetranychus urticae]
MILNTYASVNQQFSLQTFNFLICAKTMKMIATLFVVFAVASSAFGQCPVPTPVSGADIGKIAGRWYEISG
STLDLTCVTMDFTPREDGNFNYTTLGSKSDGSKHSRHLLAIRTDNENSFNISDMSTETRYPITFYIADTD
YDNYLAMYSCVFLPGSSGLFSGSILSRANSMDDEKHAELFDLLINKVGLTADKIQPITQEYCKY
>TurtLCN_XP_015783216.1 apolipoprotein D [Tetranychus urticae]
MKMIATLFVVFAVVSSALGECPVPPTIPGFDIGKIVGRWYQTVESWPENATTFTLDFTLREDGDFNFTAR
IADDSLPAEYALAKRTEYQNVLNVTDPYHKSRYQTTINIVDTDYDNYLAAYDCFIFPHHSAIHARYVLSR
TETMNADKLAQLTDLVNKIGVSRD
>TurtLCN_XP_015783215.1 apolipoprotein D-like [Tetranychus urticae]
MKMIASLFVVFAFASSALGECPVPPTTPGADIGKIAGRWYETIEILPNLTTTLTLDFTPRDDGDFNLTTH
APFSKHDEYILAKRTDNQNVLNFTDPDHKIRFQNTFNIADTDYDSYLVAYNCFIFPHHSAVHARYVLSRT
ETMNADKLAQLTDLVNKIGVSPD
>TurtLCN_XP_015783189.1 apolipoprotein D-like [Tetranychus urticae]
MKMIATLFFVFAVASSALGQCPISPTTPGSDIGKIAGRWYAIAAPTIDRFQETVTCVTEDFIFREDGNFD
YTVLGTSLDGSKTRLHWLAERTESQNAFNLTDRSKSYQVTYNIVDTDYDNYLAIYSCFYLPGYTTIQTGM
ILSRTNTMNADKYAELTDLLVNEIGVASNTFGPIVQKDCKYWPVSSHDFQT
>TurtLCN_XP_015782933.1 apolipoprotein D [Tetranychus urticae]
MDSRFNVFIVCLAIGVTFVNGQRPSPGGCPKITSNAMNNTAKFDINKYAGIWYELERSFNVFEIGLRCIT

AEYLAKSDGTVGVVNRGISKWGQKVTIEGSGRVADRSKPSYLKVAFKYSPEAPYWIADTDYTNYAIVVSC
SDLFGLFRVDSIWILSRTPDLPQSTMDALHSYVDRLGLSSSALSRVDQSNCPN
>TurtLCN_XP_015781510.1 apolipoprotein D [Tetranychus urticae]
MKMIATIFVVFAFAYSALGECPTPPVAPDDADVARIVGRWYEIARPTTASENGLTCVTCNVTPRDDGDFN
ITNLGTKPDGSMASEYAIAKRTSSQSLLHINPIAIKIPFAIPFNIAATDYDNYLVAYTCLRDVKRGWILS
RSNSMSEAQLTELTNLLATKYGVSLDETEFTSHKDCKYWPA
>TurtLCN_XP_015781509.1 apolipoprotein D [Tetranychus urticae]
MISTLILVAALAGSAYSGSCPTPPPVPAGFDITKFGGRWYEVVRTSGFTENGLRCVTADFILRSDGDFNT
TNSAIRKNGEAAIEHGTANRVKGGPADELKMVSSTIPLPIAFYIVDTDYDTYAAAYTCIGVPPLFTYETA
WVFSRKNTLDEATTKRLTDLLTSKYGIYESSLEVTNQTDCTYTPFP
>TurtLCN_XP_015781508.1 apolipoprotein D [Tetranychus urticae]
MKMIATLFFVFAFASPAFGGLFSKCPTPPTAPAGADIAKIAGRWYEIARPTKASENGLTCVTCDVTPRKD
GNFNITNLGTKPDGSKAGEYGLAKRTSSQVLLNIYSLTVKLPFPIGFNIAYTDYDNYLVAYTCLGIPPIY
TVKHGWILSRNNTISADKLTELTDLLFTKYGVSRDETEFTTQKDCKYWPVPQ
>TurtLCN_XP_015781506.1 apolipoprotein D [Tetranychus urticae]
MKMIATLFFVFAFASPAFGGWFGWCPTPPAAPEGTDITKIAGRWYEIARPTKASENGLTCVTSDFTLRPD
GDYNITNLGLKPDGSKAGEYGEAKRTDSQSLLNVYSFAVKLPFPIGFNIAEADYDDYFVAYTCIGVPPFF
TSKHAWILSRKNTMDADKLKRLTDLVINQYGVEPAEIEVTPQKDCKYWPVPQ
>TurtLCN_XP_015794744.1 apolipoprotein D [Tetranychus urticae]
MLPTFILLTLLSSSYGHILRMGDCPDVEVMRNFDMDKFLGKWFVLQKFRTMSNCMAEEITKDENGDYHIS
EFLVPLGVQIHQRGKVEFAKNEPKSAMRVDYPITTPLGSQNYWVLDTDYTSYAAVWSCQKILFGHRQSAQ
IMSRTSDLPKEKIRELRQKFESYGINEHDFSFIDQKKCESNDNKRSSINETNCTGCIKNSNFAMKVGPVR
ILTKRRR
>TurtLCN_XP_015791458.1 uncharacterized protein LOC107368188 isoform X2 [Tetranychus urticae]
MKMYVFVLLLSVLVAVGQSTKIDGPCPIKIKQIETFDPNRYLGKWWDVYANSYGVCGSGYGCQLTYYRLY
HEDIHAIPINWFTGPTYSNECSFVIYQTDYDVDPRLFYDGDDNSIYVLDTDYDSYAVEIGCANDDGVTHE
ERVVLRSRNMTMPDEVRDRLVKKVVEYGFAKPNLNAHNYTGCKDFTPTH
>TurtLCN_XP_015791442.1 uncharacterized protein LOC107368167 [Tetranychus urticae]
MKIYVSVFVLSVLVAVGQCTKIDGPCPIKIKQIETFDVNRYLGKWWDVYAGSNSYGSCGSGYGCILTYYR
LYHEDTHAIPINYFSGPDYANICSVGIWQTDYDVDGRLFYDGDDNSIYVLDTDYDSYAVEIACSNDDGTT
HEEKVVFRSRNMTMSAEVRDKLVKKVVDYGFAEPELVAHNFTGCKDFDPDFWHKH
>TurtLCN_XP_015791433.1 uncharacterized protein LOC107368158 [Tetranychus urticae]
MRMYVFVFLLCALVAVGQSAKVDGPCPIKITQIQTFDHQRYLGGWYDVYTGSNSYVRDCGSDSGCQATYY
RAYDDDTHSFYLNYYFGSDYTGLCSFPIYTTDTDVDARLFYDTDFTSLYILDTDYDSYAVEVGCSNDDGL
THEERVFIRSRNTTISDELRDQLVEKVVDFGFAEPDLIAHNFTGCPDWHPGFWHKD
>TurtLCN_XP_015788952.1 apolipoprotein D-like [Tetranychus urticae]
MNQLALISLIVLSSTLCQLGSSLSPGRCPQTTEIDGGLNLSRYLGKWYEIKRTDLIFELGLRCVKTEYTV
ESNGNVAVNNTGVSLINTQVSVVGTAKPGTQSNVFAVSFFQFSPSAQYWIVDTDYETYSLVVSCNDVLGL
FNLKNAWILSRKPNLDSDIVDQLVAKLKAIGVETDKLTTTAQDCGN
>TurtLCN_XP_015788941.1 apolipoprotein D isoform X3 [Tetranychus urticae]
MNQLALISLIVLSSTLCQLGSSLSPGRCPQTTEIDGGLNLSRYLGKWYEIKRTDLIFELGLRCVKTEYTV
ESNGNVAVNNTGVSLINTQVSVVGTAKPGTQSNVFAVSFFQFSPSAQYWIVDTDYETYSLVVSCNDVLGL
FNLKNAWILSRKPNLDSDIVDQLVAKLKAIGVETDKLTTTAQDCGN
>TurtLCN_XP_015788931.1 apolipoprotein D-like [Tetranychus urticae]
MNQLALISLIVLSSTLSVYAYPQLGSSLSPGRCPQTTEIEGGFNLTQYLGKWYEIKRTGLVFEFGQRCVK
AEYKLDSDGNVAVNNSGVNLVNFEVYSVGTAKPGTQSNVFAVTFFPLSPSSQYWIVDTDYETYSLVVSCN
DVLGLFNLKDAWILSRKPKLDEEIVKRLVDKLGKLGVPSSLLSTTTQNCDK
>TurtLCN_XP_015788922.1 apolipoprotein D isoform X1 [Tetranychus urticae]
MNKLALISLIVLSSTLSASALPNIFNLKALQDKIPQLSAVGSSFWVGRCPQTTEIKGGFNLTQYLGKWYE
IKRTGLVFEFGQRCVKAEYKLDSDGNVAVNNSGVNLVNFEIYSLGTAKPGTQSNVFAVTFFPLSPAAQYW
VVDTDYETYSLVVSCNDVLGLFNVKDAWILSRKPKLDEDIVNRLVGKLDTLGIPSFLLSSTTQNCEN
>TurtLCN_XP_015780968.1 apolipoprotein D [Tetranychus urticae]
MKMIATLFFVFAFASPAFGGWFGWCPTPPAAPEGTDITKIAGRWYEIARPTKASENGLTCVTSDFTLRPD
GDYNITNLGLKPDGSKAGEYGEAKRTDSQSLLNVYSFAVKLPFPIGFNIAEADYDDYFVAYTCIGVPPFF
TSKHAWILSRKNTMDADKLKRLTDLVINQYGVEPAEIEVTPQKDCKYWPVPQ

>VdesLCN_XP_022663986 .1 apolipoprotein D-like [Varroa destructor]
MRAHCIGAVCIFLHLFSDTLGKKIIPMENFDPEKIQGTWFVIRRNTKLFDSIKCIKYNLKHDPNIDDIYSMTSYWINSAG
EYVETQFNIVDDRALKSRFFFESTQEKIAVNVLGTDYTNWMVGFGVNGPKETYYVSTRTKSLPPDSQDAINEILKKNEVE
QDWADVEQVACPDTH
>VdesLCN_XP_022664566 .1 uncharacterized protein LOC111251803 [Varroa destructor]
MCRFNVLCFTAVAILSGVSFAEKVILMEDYDPEKVQGTWYVWRINAMIFDEVKCIKFDMLHDIGTEDLYRINSKWINTEG
QYKHTEFNVLDDKYHKAQYLFESTADKVALSIMGTDYSKWIVGYGNNLGKESYFVALRTKTVPEEVKVPIEEILKKNEIN
RDWQIIVQDDCPEEH
>VdesLCN_XP_022653633 .1 uncharacterized protein LOC111247224 isoform X1 [Varroa destructor]
MMAVFSFVLAGLLGALHLTNADEVILMEPFDPLKVTGTWRVWKRNTHLIEQTSTVRCIEVEIEHDENSGDIFKMLVKWID
PNNGNLNISFYVVDDRSHPARFFFKADQHRIALSVLGTDYNNWAVAYGMLGLKESYFVAARKLPLDDAALTDVESIISKN
QVSKDFTVVDHSSC
>VdesLCN_XP_022653636 .1 uncharacterized protein LOC111247224 isoform X2 [Varroa destructor]
MNLAFAFCNWALLRNRKVTGTWRVWKRNTHLIEQTSTVRCIEVEIEHDENSGDIFKMLVKWIDPNNGNLNISFYVVDDRS
HPARFFFKADQHRIALSVLGTDYNNWAVAYGMLGLKESYFVAARKLPLDDAALTDVESIISKNQVSKDFTVVDHSSC
>VdesLCN_XP_022671613 .1 fatty acid-binding protein-like [Varroa destructor]
MSEPGNTEVKAISLFNGIWKTVESVDYEDFLKAVDVGITWRVLSSGSKPSIEIAINGNQWTLKTHTLLKTHELKFTLGEE
FIETRLDGVRVKTTCTLENGKLVQRSLGDKEIIIVRELHGDDLLKTTFSYKDITAIRVYKRSTRITR
>VdesLCN_XP_022665322 .1 fatty acid-binding protein homolog 5-like [Varroa destructor]
MAAAFVGSWKLTESEKFDDFLKELGVNMVLRKFAQAATPTVNISNEGDKWKIETITTMKTSLIEFKMGEEFLEKRLDGNE
VRSVMTMDGNKMIQKQFADEAKKLKEVTITRWIDGNRLLVEAAVSDIVSTRKYDRQ
>VdesLCN_XP_022661284 .1 fatty acid-binding protein, heart-like [Varroa destructor]
MVDALIGTWKLETSENFEELLKELGVNMVLRKAATATKPNAEISKNGDEWTIRSVSTLKTTEIKFKIGEEFEETRADNSK

GKSTFIIDGDKLKQVSEKDGKQYSTVREIVGGKLKVTVTIGNIVCTRVYSKV
>VdesLCN_XP_022662731 .1 insecticyanin-B-like [Varroa destructor]
MTRVPARALSLVTLLCGQVRLTQLLMLFLVRCTDAMVMPGLCPTEIKTVDDFDHDRFMGTWYEVQRTPFLMETMIRCNKF
HYQDKGDSEEGIIVYMKGNRQSVSAMLSSTR
>VdesLCN_XP_022658018 .1 apolipoprotein D-like [Varroa destructor]
MKFARAGSERCSVSGVSNRCCGVICARKVRDATSAEPPTGSVRGPATRRASAYCCRPLTLFALLGLVGFAQAHQYSLGAC
PPEVPFKKSFDVDRFLGVWFVIQKSHSTMSCLKVNITRRGDEDFKITEYRRFGLLQKAVDHTLIDVGVLKIPDPTEPAKM
NVKFSLSAWVEPFTVMDTDYDTYAATFSCVNAAGMGHRKNGMILSRRPDLDNTVADKLRKLFDSFGVGPSSLSFIDQKHS
CTSMLKSKNDHDRVISLGPLNINRKPEP

>CscuLCN_XP_023223180 .1 apolipoprotein D-like [Centruroides sculpturatus]
MILVEPIERTTLYVLYFALHQFLGEWYVIQRFQSNNRCIKQNISKTDDYAYKITYKLSETRKSTTLSFLGLEQMIEQSSN
IHFPYSNISSKMEVNLPLSVWGASNYWVVMTDYTMYAAVWSCRNMFFGQVQSADILSRTPRLDRLIIDKIRRRFEGYGIN
VNHFSVIDHSECPEAERRRSSFFVLLV
>CscuLCN_XP_023240882 .1 apolipoprotein D-like [Centruroides sculpturatus]
MTWVVACIFIYQAVTVLSQRPFSGPCPKHAAQEGFDMGSFLGVWYEIEKTSLVQDGVRCIKTKYIKTNDGKLTLTTTGIN
LLGIEQSSRGEAVLESLDPATLLVQYNTIIGLPVSTTLKILDTDYKNYAVVWSCSPVLGPLGHTENLWLLSRKTSMDPDV
RKTLMERLTTLNIDHEGLRTISHQNCT
>CscuLCN_XP_023240879 .1 apolipoprotein D-like [Centruroides sculpturatus]
MKKLGLFISIYLLQCIYVNGQIHFAGSCPNPTPMEDFDIVKFAGKWYEIERTFSIFGFPWNCPTTQITITGEGVEMERGV
FASTLSANHYNRIALAKLNVRDRRQPAKMEFRMPGITEPIKYYIMDTDYDNYTLAWGCYEASNLRGTFGHFEYIWLLSRT
PKMDTAVQQELYTLMDDRGIRRLRLTRNRMDNCEEKVN
>CscuLCN_XP_023222347 .1 apolipoprotein D-like [Centruroides sculpturatus]
MRDSKKVYFESITMEKIMSGFILILCLISFVKCQSYFSSSCPSPAPIKDFNITAFTGKWYEVESTRSIFETFWNCPTTTI
SITGEGTEIERGGYVSLLNRNFYNRIVRTKLNAPDARRPGRLEFNFPGITQPLKYVILDTDYDNYALAWSCYEFGRLGGT
LGHFEYIWVLSRHVHTDKEVKQKLYTALDDNGIRRFYITKNRMDECHSKP
>CscuLCN_XP_023226368 .1 apolipoprotein D-like [Centruroides sculpturatus]
MFQFQGVWYEIKRTFTLFETGLKCSATIITNVENGNALFRRTGVNIINRHFIIMQGKLRTVNSISPANMELTLGALRFRS
IYKIIDTNYEDYAAVWSCYESPLLAGTLGHTENLWILSRQILLKPEYLNKMFDVIDSLNISRATLIDTNFNNCTDISGRL
NSKLSSHF

Table S1. Sequence coverage of lipocalin genes in the antennae and other organs of selected species. M: male; F: female; Ant: Antennae; L: Legs; Phe: Pheromone glands; Sal: Salivary glands; Abd: Abdomen. Tblastn was performed searching the following specific SRA databases. *Bombyx mori*: Ant-M: SRX3924058; Ant-F: SRX3924059. *Anopheles gambiae*: Ant-M: SRX552892; Ant-F: SRX336124; *Aedes aegypti*: Ant-M: SRX468763; Ant-F: SRX468786. *Culex quinquefasciatus*: Ant: SRX352802; Hind-L: SRX352803. *Rhodnius prolixus*: Ant-M: SRX1011778; Ant-F: SRX1011769. *Leptinotarsa decemlineata*: Ant-M: SRX974484 Ant-F: SRX974488. *Helicoverpa armigera*: Phe: SRX692537; Ant: SRX3595782; Sal: SRX3595774; Abd: SRX3595762. *Varroa destructor*: Phoretic forelegs SRX4157185; Reproductive forelegs; SRX4157183; Rear legs: SRX4157188.

LCN acc. no.	Sequence coverage%		LCN acc. no.	Sequence coverage%	
Bombyx mori	Ant-M	Ant-F	*Culex quinquefasciatus*	Ant	Hind-L
Bmor_XP_004932390	77	54	CquiLCN_XP_001863795	37	50
Bmor_XP_004932389	65	67	CquiLCN_XP_001851241	27	0
Bmor_NP_001140192	15	4	CquiLCN_EDS26941	44	35
Bmor_ANU05020	17	15	CquiLCN_XP_001870653	65	23
Bmor_NP_001036872	16	16	CquiLCN_XP_001869618	0	0
Bmor_XP_004921596	20	22	CquiLCN_XP_001866789	0	29
Bmor_XP_004923379	0	5	CquiLCN_XP_001868835	91	71
Bmor_XP_004926879	63	78	CquiLCN_XP_001866793	15	0
Bmor_XP_004923537	29	50	CquiLCN_XP_001866792	83	68
Bmor_XP_004923378	36	21	CquiLCN_XP_001866791	0	53
Bmor_XP_012553141	59	68	CquiLCN_XP_001866788	28	0
Bmor_XP_004923380	0	0	CquiLCN_XP_001863495	71	41
Anopheles gambiae	Ant-M	Ant-F	*Rhodnius prolixus*	Ant-M	Ant-F
Agam_XP_320076	0	87	RproLCN_AAQ20818	55	56
Agam_XP_320077	0	0	RproLCN_JAA77437	23	0
Agam_XP_003436167	0	0	RproLCN_JAA77174	95	97
Agam_XP_563568	18	82	RproLCN_JAA76575	31	0
Agam_XP_003436166	5	0	RproLCN_JAA76312	100	88
Agam_XP_563569	0	0	RproLCN_JAA75419	22	20
Agam_XP_001689062	58	50	RproLCN_JAA75158	33	13
Agam_XP_312344	55	73	RproLCN_JAA76784	0	28
Agam_XP_003436165	0	0	RproLCN_JAA76106	0	16
Aedes aegypti	Ant-M	Ant-F	*Leptinotarsa decemlineata*	Ant-M	Ant-F
AaegLCN_EAT40277	26	13	LdecLCN_XP_023016063a	20	30
AaegLCN_EAT38566	54	50	LdecLCN_XP_023016063b	17	45
AaegLCN_EAT38565	36	54	LdecLCN_XP_023025211	91	93

AaegLCN_EAT38564	0	74
AaegLCN_EAT38561	55	62
AaegLCN_EAT38559	31	29
AaegLCN_EAT38558	58	48
AaegLCN_EAT36662	44	45
AaegLCN_EAT34157	23	52
AaegLCN_XP_001660231	0	89
AaegLCN_XP_001660226	0	19
AaegLCN_XP_001660228	59	62
AaegLCN_XP_001660229	72	69
AaegLCN_XP_001660232	48	87
AaegLCN_XP_021709994	82	33
AaegLCN_XP_001660225	68	47
AaegLCN_XP_001660233	80	80
AaegLCN_XP_021703438	26	13
AaegLCN_XP_021699910	45	43
AaegLCN_XP_021697582	83	87

LdecLCN_XP_023024212	54	54
LdecLCN_XP_023019747	58	61
LdecLCN_XP_023019746	62	59
LdecLCN_XP_023019744	58	60
LdecLCN_XP_023019743	41	38
LdecLCN_XP_023029793	87	90
LdecLCN_XP_023019521	83	89

Varroa destructor	Phoretic forelegs	Reprod. forelegs	Rear legs
VdesLCN_XP_022663986	76	78	23
VdesLCN_XP_022664566	45	49	99
VdesLCN_XP_022653633	55	60	0
VdesLCN_XP_022653636	53	65	0
VdesLCN_XP_022671613	77	28	66
VdesLCN_XP_022665322	69	23	69
VdesLCN_XP_022661284	86	89	80
VdesLCN_XP_022662731	92	0	41
VdesLCN_XP_022658018	57	57	57

LCN acc. no.	Sequence Coverage%			
	Phe	Sal	Ant	Abd
HarmLCN_AFK64814	88	87	25	48
HarmLCN_XP_021200319	100	21	98	100
HarmLCN_XP_021200364	60	14	53	76
HarmLCN_XP_021182455	87	78	14	17
HarmLCN_XP_021185371	98	84	73	92
HarmLCN_XP_021190587	89	20	100	100
HarmLCN_XP_021190592	0	71	34	55
HarmLCN_XP_021190608	70	72	55	69

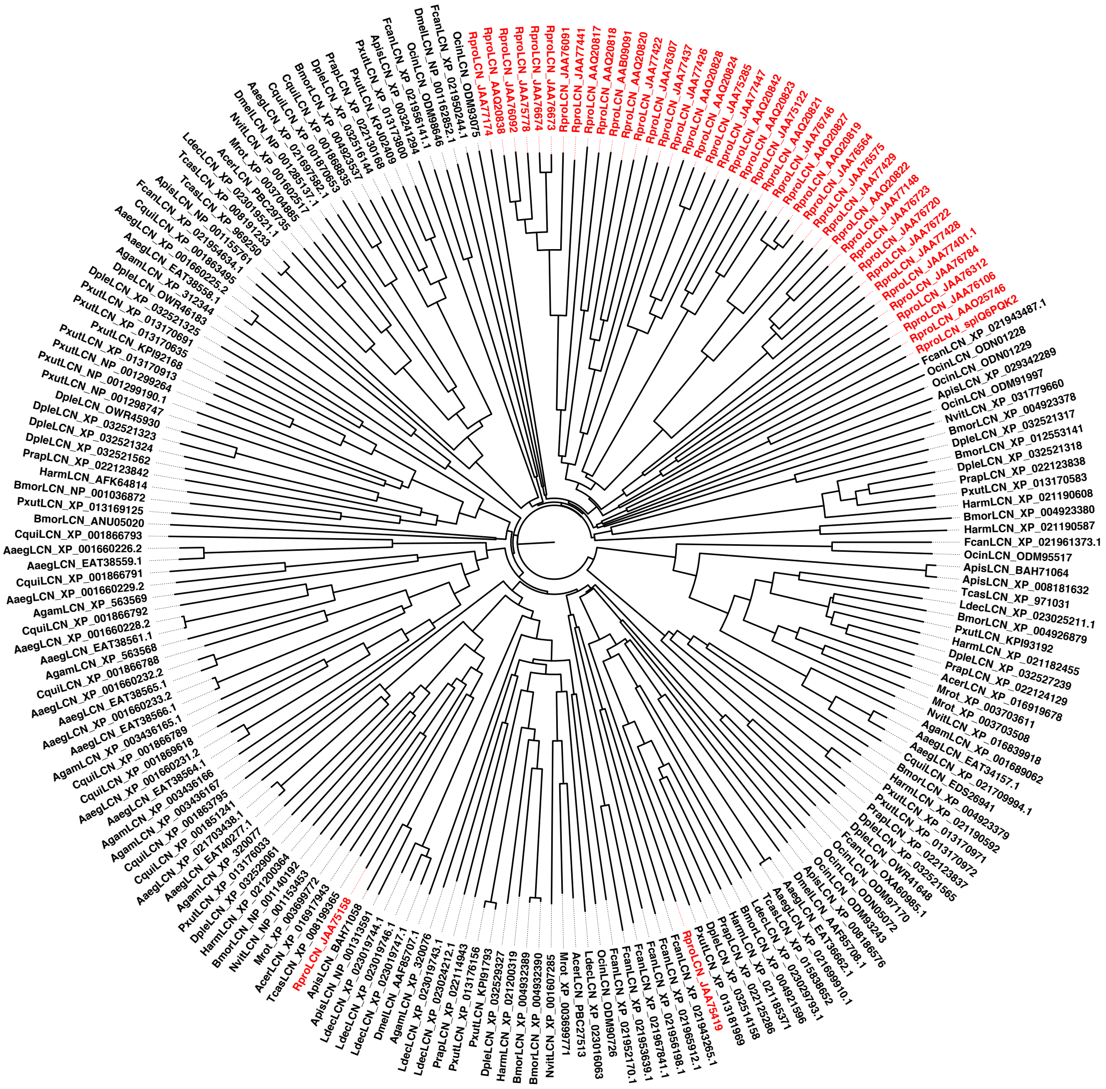

Figure S1. Phylogenetic tree of lipocalins identified in the genomes of selected species of Hexapoda. Apis: *Acyrthosiphon pisum*; Aaeg: *Aedes aegypti*; Agam: *Anopheles gambiae*; Acer: *Apis cerana*; Bmor: *Bombyx mori*; Cqui: *Culex quinquefasciatus*; Dple: *Danaus plexippus*; Dmel: *Drosophila melanogaster*; Fcan: *Folsomia candida*; Harm: *Helicoverpa armigera*; Ldec: *Leptinotarsa decemlineata*; Mrot: *Megachile rotundata*; Nvit: *Nasonia vitripennis*; Ocin: *Orchesella cincta*; Pxut: *Papilio xuthus*; Prap: *Pieris rapae*; Rpro: *Rhodnius prolixus*; Tcas: *Tribolium castaneum*. Nearly all the sequences of the hemipteran *Rhodnius prolixus* segregate into a separate clade and are reported in red font.

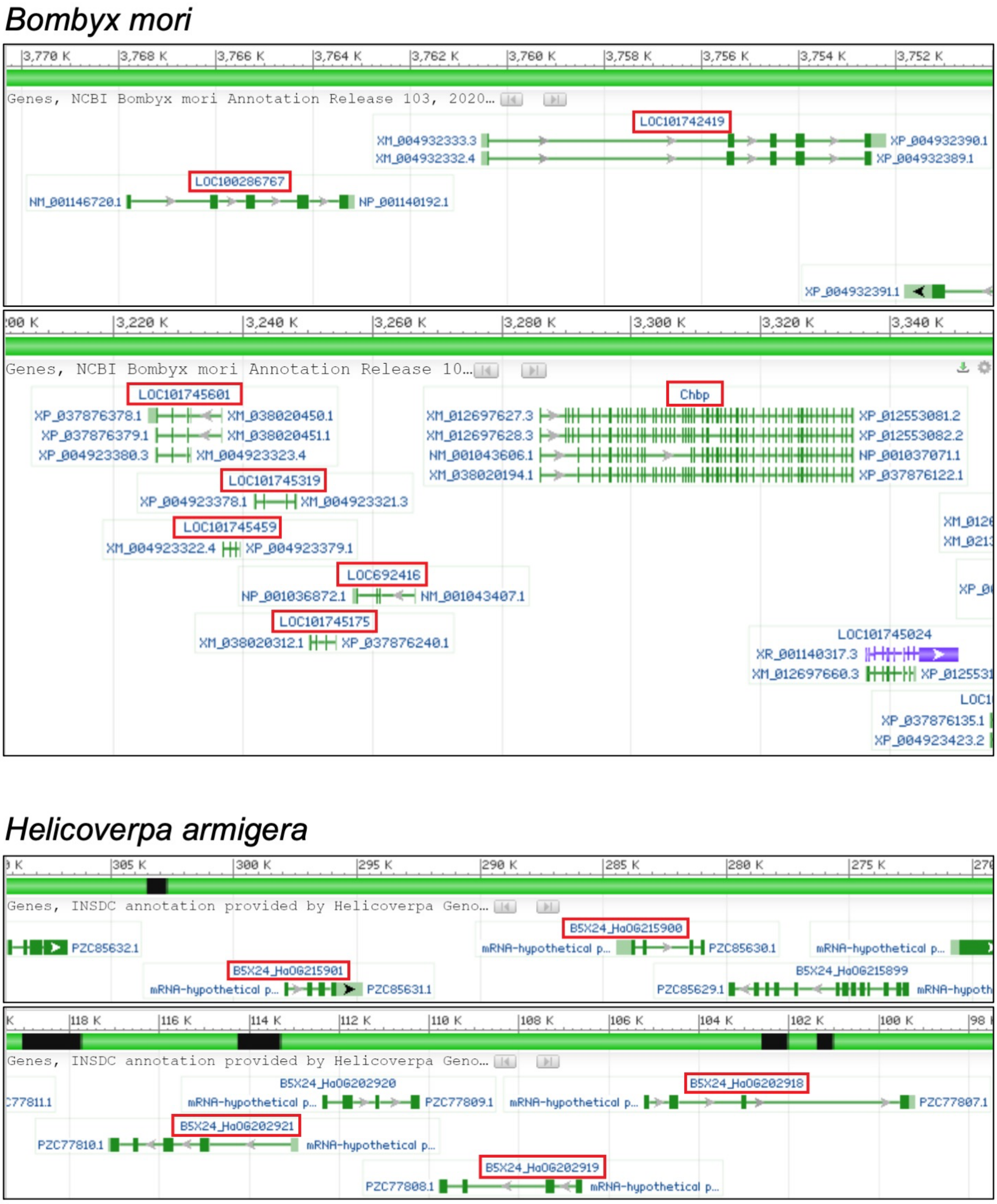

Figure S2. Location of lipocalins in the genomes of *B. mori* and *H. armigera*.
